# Lasting connectivity increase and anxiety reduction via transcranial alternating current stimulation

**DOI:** 10.1093/scan/nsy096

**Published:** 2018-10-30

**Authors:** Kevin J Clancy, Sarah K Baisley, Alejandro Albizu, Nika Kartvelishvili, Mingzhou Ding, Wen Li

**Affiliations:** 1 Department of Psychology, Florida State University, Tallahassee, FL, USA; 2 J. Crayton Pruitt Family Department of Biomedical Engineering, University of Florida, Gainesville, FL, USA

**Keywords:** alpha oscillations, resting state, Granger causality, neuromodulation, sensory affect

## Abstract

Growing evidence of transcranial alternating current stimulation (tACS) modulating intrinsic neural oscillations has spawned interest in applying tACS to treat psychiatric disorders associated with aberrant neural oscillations. The alpha rhythmic activity is known to dominate neural oscillations at the awake, restful state, while attenuated resting-state alpha activity has been implicated in anxious mood. Administering repeated alpha-frequency tACS (α-tACS; at individual peak alpha frequency; 8–12 Hz) over four consecutive days (in the experiment group, sham stimulation in the control group), we demonstrated immediate and lasting (>24 h) increases in resting-state posterior ➔frontal connectivity in the alpha frequency, quantified by Granger causality. Critically, this connectivity enhancement was accompanied by sustained reductions in both anxious arousal and negative perception of sensory stimuli. Resting-state alpha power also increased, albeit only transiently, reversing to the baseline level within 24 h after tACS. Therefore, the lasting enhancement of long-range alpha connectivity due to α-tACS differs from local alpha activity that is nonetheless conserved, highlighting the adaptability of alpha oscillatory networks. In light of increasing recognition of large-scale network dysfunctions as a transdiagnostic pathophysiology of psychiatric disorders, this enduring connectivity plasticity, along with the behavioral improvements, paves the way for tACS applications in clinical interventions of psychiatric ‘oscillopathies’.

## Introduction

Brain oscillations play important roles in various mental activities (Başar *et al.*, 2001; Buzsaki *et al.*, 2013). Aberrations in these oscillations have been observed in a host of neuropsychiatric disorders, potentially underpinning their pathophysiology (i.e. ‘oscillopathies’; Basar, 2013; Buzsaki *et al.*, 2013). Recent advances in transcranial alternating current stimulation (tACS) demonstrate that by applying weak electric fields tuned to the rhythms of endogenous oscillations through the scalp, tACS can directly interact with and thereby modify brain oscillations (Frohlich and McCormick, 2010; Thut *et al.*, 2011; Antal and Paulus, 2013; Herrmann *et al.*, 2013). This non-invasive neuromodulation technology thus holds great promise for therapeutic interventions of oscillopathies (Frohlich, 2015; Abend *et al.*, 2016). However, clinical efficacy of tACS hinges on whether its neuromodulatory effects would persist and induce lasting behavioral changes.

Alpha oscillations (8–12 Hz; posteriorly distributed) are the most prominent rhythmic activity in the awake restful human brain and are thought to be generated in the thalamo-cortical network (Hughes and Crunelli, 2005; Bollimunta *et al.*, 2008; Buzsaki *et al.*, 2013). Alpha oscillations exert largely inhibitory modulation on multiple cognitive processes, including attention and sensory perception (Palva and Palva, 2007; Bollimunta *et al.*, 2008; Foxe and Snyder, 2011; Klimesch, 2012). For example, heightened pre-stimulus alpha power is associated with reduced perceptual sensitivity to the stimuli (Mathewson *et al.*, 2009; Dugue *et al.*, 2011), and attenuated alpha power is coupled with enhanced visual attention (Rajagovindan and Ding, 2011). Furthermore, via long-range, posterior ➔ frontal projections, alpha oscillations mediate inhibitory bottom-up information flow from the sensory cortex to frontal regions to influence global neural activity (Hillebrand *et al.*, 2016; Sadaghiani *et al.*, 2016; Johnson *et al.*, 2017; Tang *et al.*, 2017a). For instance, weak posterior ➔frontal alpha connectivity is associated with strong frontal gamma activity and impaired cognitive control (Clancy *et al.*, 2017; Johnson *et al.*, 2017).

Alpha oscillations are particularly important for inter-regional communication across large-scale resting-state networks (RSNs), especially the default mode network (DMN) and the salience network (SN; Laufs *et al.*, 2003; Mantini *et al.*, 2007; Sadaghiani *et al.*, 2010; Mo *et al.*, 2013; Tang *et al.*, 2017a), which are strongly implicated in emotion- and arousal-related processes (Raichle *et al.*, 2001; Sridharan *et al.*, 2008) and affective disorders (Sripada *et al.*, 2012; Peterson *et al.*, 2014; Alvarez *et al.*, 2015). Furthermore, akin to the reciprocal roles these two RSNs play in regulating arousal (Menon and Uddin, 2010; Young *et al.*, 2017), alpha activity is positively correlated with DMN activity and negatively with SN activity (Sadaghiani *et al.*, 2010; Ros *et al.*, 2013), serving a key function in maintaining a state of restful alertness (Gath *et al.*, 1983; Klimesch, 1999). Relatedly, neurofeedback training targeting alpha activity has been shown to modulate DMN and SN activities (Ros *et al.*, 2013) and reduce anxiety (Hammond, 2005), while meditation training that leads to reduced anxious mood has been linked to augmentation of resting-state alpha activity and reorganization of these RSNs (Cahn and Polich, 2006; Fan *et al.*, 2014; Tang *et al.*, 2017b)*.*

Consistent with growing evidence of DMN and SN dysfunctions in post-traumatic stress disorder (PTSD; Sripada *et al.*, 2012), recent data from our laboratory revealed deficits in resting-state alpha power and posterior ➔frontal alpha connectivity in patients with PTSD (Clancy *et al.*, 2017). Importantly, reduced alpha connectivity correlated with greater hypervigilance, a key hyperarousal symptom of PTSD. These findings thus implicate reduced alpha activity that translates to behavioral expressions of hypervigilance and anxious arousal, thereby presenting a concrete and spatiotemporally specific target for clinical intervention via alpha-frequency tACS (α-tACS). That is, provided alpha enhancement via α-tACS could persist, tACS could achieve stable anxiety reduction and clinically meaningful symptom alleviation.

Empirical and computational models suggest that while tACS induces short-term effects via entrainment of local neuronal firing synchrony, long-term plastic effects would arise from strengthened oscillatory circuits via spike-timing-dependent plasticity (STDP) and long-term potentiation (LTP) at the synapse (Thut *et al.*, 2011; Herrmann *et al.*, 2013; Reato *et al.*, 2013). ‘Plasticity-like’ effects of tACS (primarily, local power enhancement outlasting the period of stimulation) have been consistently demonstrated (Neuling *et al.*, 2013; Vossen *et al.*, 2015; Kasten *et al.*, 2016), but evidence for long-term effects remains limited. A recent α-tACS study tracked alpha power change over 90 min post-stimulation, but in contrast to enduring LTP that typically lasts for hours to days (Abraham, 2003), alpha power enhancement persisted only for 70 min post-stimulation (Kasten *et al.*,  2016).

The acute but transient tACS effects could reflect the labile, early phase of LTP that is susceptible to other synaptic activities such as endogenous, corrective inputs from other neurons and changes in the extracellular environment (Abraham, 2003; Huang *et al.*, 2017). Targeting primarily the cortex (Frohlich and McCormick, 2010; Thut *et al.*, 2011; Herrmann *et al.*, 2013), exogenous oscillatory inputs of most tACS applications rarely reach deep subcortical generators (Neuling *et al.*, 2012). Since the thalamo-cortical circuit operates as a unified generator of alpha oscillations (Hughes and Crunelli, 2005; Bollimunta *et al.*, 2008; Buzsaki *et al.*, 2013), deep thalamic drives can thwart early-phase LTP at the synapses, resetting cortical alpha oscillations after α-tACS. That said, as repeated stimulation can facilitate the induction of late-phase LTP and long-term neural plasticity (Fregni *et al.*, 2006; Reis *et al.*, 2009; Huang *et al.,*^2017^), lasting tACS effects may emerge with multi-session (*vs* single-session reported thus far) tACS protocols.

Besides local cortical power increases, tACS, via the purported synaptic strengthening, is likely to induce inter-cortical connectivity plasticity. Indeed, tACS can intensify rhythmic reverberations across the abundant recurrent inter-cortical connections in the brain, thereby modifying large-scale network dynamics (Clopath *et al.*, 2010; Alagapan *et al.*, 2016; Moisa *et al.*, 2016). This account would hold especially true for α-tACS given the instrumental role of alpha oscillations in long-range interactions (Palva and Palva, 2007; Cabral-Calderin *et al.*, 2016). Importantly, while local cortical alpha oscillations can be restored by thalamic inputs, plasticity in inter-cortical connectivity, presumably relatively independent of thalamic drives, may persist over time and mediate lasting anxiety reduction.

**Fig. 1:**
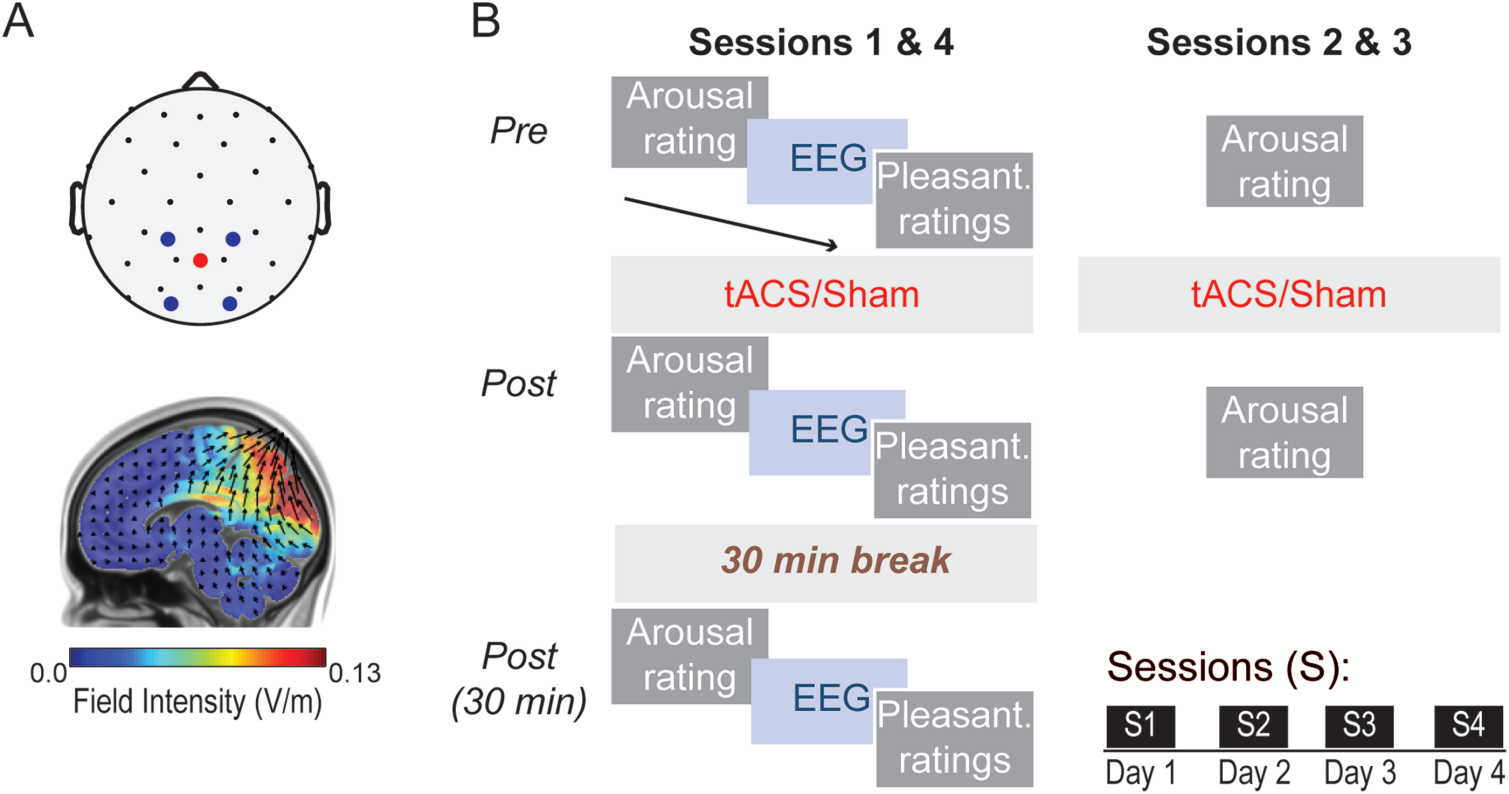
Stimulation setup and experimental protocol. **(A)** Top: tACS montage, with stimulation electrodes placed over occipitoparietal sites. Bottom: Modeled current flow of tACS, demonstrating maximal electrical field intensity over the occipitoparietal cortex. **(B)** Participants completed four sessions of tACS or sham stimulation, each separated by 24 h. tACS was administered for 30 min at each individual’s peak alpha frequency (PAF). Eyes-open resting state EEG data and ratings of anxious arousal and perceived pleasantness of auditory and olfactory stimuli were acquired before, immediately and 30 min after stimulation.

Therefore, administering repeated sessions of high-density (HD) α-tACS targeting the occipitoparietal cortex implicated in PTSD-related alpha deficiency (Clancy *et al.*, 2017; Figure 1), we set out to test two hypotheses. First, α-tACS could generate lasting enhancement of alpha activity, including alpha power and long-range connectivity, particularly posterior ➔frontal projections that dominate resting-state alpha connectivity (Engel *et al.*, 2001; Tang *et al.*, 2007; Sadaghiani *et al.*, 2012; Hillebrand *et al.*, 2016; Wang *et al.*, 2016). Second, these neural changes would be accompanied by lasting anxiety reduction, measured here by anxious arousal and negative perception of sensory stimuli (in the auditory and olfactory modalities given their sensitivity to states of anxious arousal) (Adler *et al.*, 1988; Grillon *et al.*, 1991; Krusemark *et al.*, 2013). Assessments were administered immediately and 30 min after α-tACS on the first and last (fourth) day, except for anxious arousal that was rated daily to track sustained and accumulating effects of α-tACS on this key outcome.

**Fig. 2:**
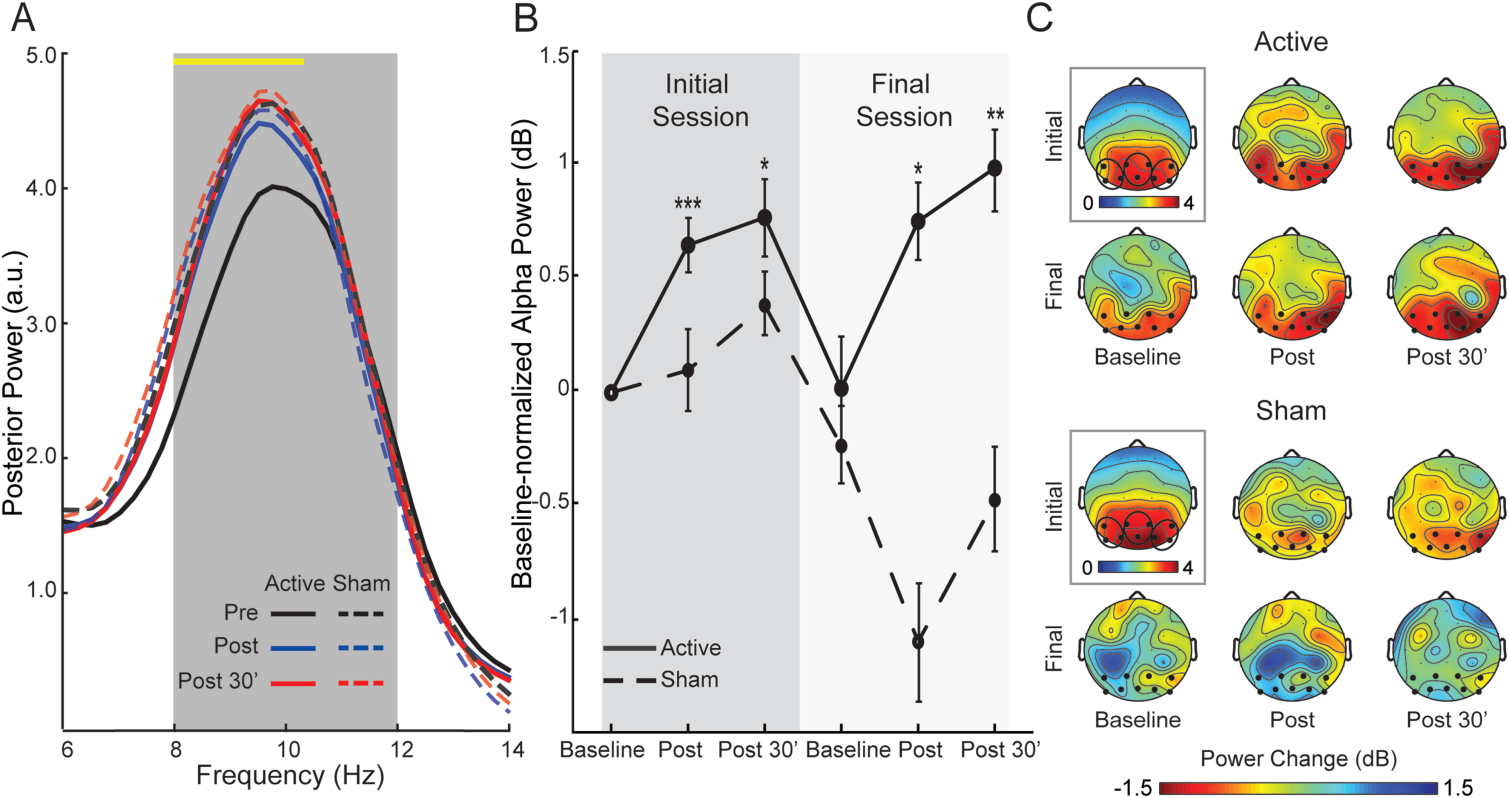
Changes in posterior alpha power. **(A)** Spectra of normalized power (collapsed across occipitoparietal electrodes) for each group at the three time points, averaged across the initial and final sessions. The yellow line at the top of the waveforms indicates frequency bins (0.25 Hz each) showing a significant Time-by-Group interaction. The interaction effect appeared selectively in the lower half of the targeted alpha range (8–10.5 Hz), confirming the specific modulation of alpha power. Note, the Active group demonstrated post-stimulation increases in the alpha band from the baseline while the Sham group demonstrated equivalent alpha power before and after stimulation. **(B)** Alpha power magnitudes, decibel-normalized to the initial baseline, for each group. **(C)** Scalp topographical maps of normalized alpha power at the initial baseline (surrounded by a black box and associated with its own color scale) and decibel-normalized power changes at each assessment thereafter. Electrodes included in occipitoparietal sites are bolded and encircled. Error bars = SEM. ^*^P < 0.05; ^**^P < 0.01; ^***^P < 0.005. All *P-*values are relative to the initial baseline. Note that the groups did not differ in the initial baseline values (*P* > 0.19).

## Methods

### Participants

Thirty-eight healthy volunteers (18 female, 19.7 ± 2.0 years of age) participated in this study after providing written, informed consent approved by the Florida State University Institutional Review Board. No participants reported a history of neurological or psychiatric disorders and all were deemed tACS compatible (e.g. no metal implants, neurological surgery or pregnancy). Participants were randomly assigned to two groups, an Active (*N* = 21) and a Sham control group (*N* = 17). Four participants in the Active group did not provide behavioral ratings and were thus included in the electroencephalogram (EEG) analyses only. Groups did not differ in age or gender distribution (*P* > 0.14)*.* Participants were blind to their group assignment (see tACS procedures below).

### Ratings and questionnaires

#### Pleasantness ratings

Auditory and olfactory stimuli were rated for perceived pleasantness on a visual analog scale presented on a computer monitor (0–100 from most unpleasant to most pleasant; 50 = neutral). Auditory stimuli included a neutral (flat tone) and two negative (screaming and vomiting) sounds, delivered through headphones for one second each. Olfactory stimuli included a neutral (acetophenone; Fisher Scientific, NH, USA) and negative (burning rubber; ScentAir™, NC, USA) odors, delivered through a bottle. All stimuli were presented at three intensities: weak, medium and strong.

#### Subjective units of anxious arousal

Participants rated their current state of anxious arousal using a visual analog scale from 0 (not at all) to 100 (extremely), in accordance with standard ratings of subjective units of distress (Wolpe and Lazarus, 1966).

### tACS

tACS was administered using a Soterix Medical 4 × 1 HD transcranial electrical stimulation system (New York, NY, USA).Stimulation electrodes were placed in a 4 × 1 montage over occipitoparietal sites, with the central electrode receiving electrical current from the four surrounding electrodes (Figure 1A). Stimulation sites were selected to maximally target the occipitoparietal area (cuneus/precuneus) where reduced alpha oscillations in patients with PTSD have been observed (Clancy *et al.*, 2017). Current flow modeling (Soterix Medical HDExplore Software) indicated maximal electric field intensity (0.21 V/m; with a ±2 mA stimulation current) in the right dorsal occipital cortex (peak voxel: 29,−91, 18; Montreal Neurological Institute coordinates; Figure 1A). Electric field intensity did not exceed 0.02 V/m in frontal regions.

Stimulation was administered with eyes open for 30 min using a ±2 mA sinusoidal current oscillating at individual participants’ occipitoparietal PAF. The PAF was identified as the peak frequency within the alpha range (8–12 Hz) with a 0.5 Hz frequency resolution across occipitoparietal electrodes during the initial (session 1) baseline EEG recordings. Individual PAFs ranged from 8–11.5 Hz (*M* = 9.93, s.d. = 0.66 Hz) in the sample, equivalent between the two groups (*P* = 0.853). All participants were familiarized with tACS-induced skin sensations with a brief, 30 s pulse of random noise stimulation. During stimulation, the current ramped up to 2 mA over a span of 10 s. Stimulation was then discontinued in Sham participants and reintroduced during the last 10 s to mitigate awareness of experimental condition. All participants were told that they would receive stimulation, and their lack of awareness of sham stimulation was confirmed during the debriefing at the end of the study and by their responses to the Adverse Effects Questionnaire (Supplementary data).

### Experimental design

The experiment consisted of four sessions, each separated by 24 h (Figure 1B). During the first and last sessions, participants started with a subjective rating of anxious arousal, followed by a 2 min resting, eyes-open EEG recording and then pleasantness ratings. Participants then received 30 min of α-tACS and then repeated a set of arousal ratings, resting EEG recordings and pleasantness ratings. After a 30 min break, participants again repeated a set of arousal ratings, resting EEG recordings and pleasantness ratings. The second and third sessions included α-tACS and pre-/post-stimulation ratings of anxious arousal.

### EEG acquisition and analyses

EEG data were recorded from a 32-channel BrainProducts actiChamp system (Munich, Germany; 1000 Hz sampling rate, 0.05–200 Hz online bandpass filter, referenced to the FCz channel). Electro-oculogram (EOG) was recorded using four electrodes with vertical and horizontal bipolar derivations. EEG/EOG data were downsampled to 250 Hz, high-pass (1 Hz) and notch (60 Hz) filtered and re-referenced to the average of all EEG channels. We applied the ‘Fully Automated Statistical Thresholding for EEG artifact Rejection’ algorithm for artifact detection and rejection (Nolan *et al.*, 2010).

#### Power analyses

EEG oscillation power was computed for individual channels for each epoch (1 s) using the multitaper spectral estimation technique (Mitra and Pesaran, 1999). Alpha (8–12 Hz) power was normalized by the mean power for the global spectrum (1–50 Hz) within each epoch. Alpha power was extracted from occipitoparietal (right, middle and left) electrodes, where they were maximally distributed (Palva and Palva, 2007; Foxeand Snyder, 2011; Klimesch, 2012).

#### Directed alpha-frequency connectivity (Granger causality) analyses

Alpha-frequency Granger causality (GC) analysis (Geweke, 1982; Ding *et al.*, 2006) was performed to assess posterior ➔frontal causal connectivity in the alpha band. Following transformation into reference-free current source density data using the surface Laplacian algorithm (Perrin *et al.*, 1989; Nunez *et al.*, 1997; Wang *et al.*, 2016), EEG data from ipsilateral frontal–posterior pairs were submitted to bivariate autoregressive (AR) modeling, from which GC spectra were derived (Ding *et al.*, 2000, 2006). A model order of 20 (80 ms in time for a sampling rate of 250 Hz) was chosen in a two-step process: (i) Akaike Information Criterion (AIC) and (ii) comparing spectral estimates obtained by the AR model and by the Fourier-based method for data pooled across all subjects (Wang *et al.*, 2016).

### Statistical analyses

Three key effects were directly relevant to our hypotheses: (i) immediate and short-term (30 min) effects based on within-session changes (effect of Time); (ii) long-term effects based on differences between the initial and final sessions (i.e. effects lasting over 24 h from the preceding stimulation; effect of Session); and (iii) improved within-session tACS effectiveness, i.e. whether within-session effects increased from the initial to the final session (interaction of Time-by-Session). As such, we examined interactions of Group with Time and/or Session, in that these time/session effects differed between Active and Sham groups. Wherever appropriate, we started the analysis with an omnibus analysis of variance (ANOVA) and followed significant effects with post hoc tests, which were further controlled for Type I error using the false discovery rate (FDR) criterion.

Occipitoparietal alpha power and feedforward alpha GC at each time point (*t*) were decibel-normalized relative to the initial (*i*), first-session pre-tACS and baseline values [power*_db_* = 10*log10(power*_t_*/power*_i_*); GC*_db_* = 10*log10(GC*_t_*/GC*_i_*)] to control for variations in baseline activity (Cohen, 2014; Vossen *et al.*, 2015; Kasten *et al.*, 2016). Note that the groups did not differ in initial baseline alpha power or GC (*P* > 0.19). Decibel-normalized values were submitted to omnibus repeated-measures ANOVAs (rANOVAs) of Site (power: left, middle, right; GC: left, right hemisphere), Session (initial, final), Time (pre, post, post-30 minutes), and Group (active, sham). Site was included to consider previously reported effects of right-hemisphere laterality of alpha power on directed attention (Corbetta and Shulman, 2011; Rajagovindan and Ding, 2011) and connectivity deficits in PTSD (Clancy *et al.*, 2017).

Pleasantness and anxious arousal ratings at each time point (*t*) were normalized using the difference-over-sum method to the initial (*i*) baseline [(rating*_t_* − rating*_i_*)/(rating*_t_* + rating*_i_*)] to account for variations in baseline ratings (Cohen and Maunsell, 2010). The groups did not differ in initial baseline ratings (*P* > 0.12). Pleasantness ratings were submitted to omnibus rANOVAs of Valence (neutral, threat), Intensity (strong, medium, weak), Session (initial, final), Time (pre, post, post-30 minutes), and Group. Ratings of anxious arousal were submitted to an omnibus rANOVA of Session (initial, second, third, final), Time (pre, post) and Group. As post-30 min anxious arousal ratings were acquired only in the first and last sessions and there was no difference between immediate and 30 min ratings in either sessions (*P* > 0.36), we averaged the immediate and 30 min ratings to reflect post-stimulation ratings for each session. Individual differences in trait anxiety are known to be associated with state anxiety and so can influence anxious arousal ratings (especially over repeated measurements across the 4 days). Therefore, we decided a priori to include scores of the Behavioral Inhibition Scale (BIS; Carver and White, 1994; a measure of trait anxiety) as a covariate in the ANOVA for anxiety ratings (not for other dependent variables).

Finally, a prospective examination of anxiety reduction on subsequent days (Days 2–4) as predicted by Day 1 alpha enhancement was conducted. This analysis was restricted to Day 1 alpha changes as the predictor because EEG was not acquired on Days 2 and 3 in order to ease the burden on participants. As the key outcome measure of the study, anxious arousal ratings were collected each day, which allowed for examination of long-term effects of Day 1 alpha and/or GC enhancement on subsequent anxiety levels. Specifically, we entered baseline shifts in anxious arousal on Days 2–4 (from Day 1) into an rANCOVA with Session (Sessions 2, 3 and 4) as an independent variable and Day 1 alpha activity change (power/GC) as a covariate. Additional correlational analyses on pleasantness ratings are reported in the Supplementary data.

To control for potential experimenter biases and to replicate the anxiety reduction effect, we conducted a double-blind, single-session replication study (on a smaller scale), explained in greater detail in the Supplementary data.

## Results

### tACS induced immediate and short-term increases in occipitoparietal alpha power

An omnibus rANOVA of Site (left, middle, right), Session (initial, final), Time (pre, post, post-30 min) and Group (active, sham) on posterior alpha power revealed a significant interaction of Time and Group (*F_1.99, 71.79_* = 3.35, *P =* 0*.*041, *η*_p_^2^ = 0.09), such that, across occipitoparietal sites and the two sessions, an effect of Time arose in the Active group (*F_1.55, 30.98_* = 7.99, *P =* 0*.*003, *η*_p_^2^ = 0.29) but not the Sham group (*P =* 0*.*485; Figure 2A). Follow-up contrasts within the Active group demonstrated power (collapsed across three posterior sites) increases both immediately (*P =* 0*.*0003) and 30 min (*P =* 0*.*006) after stimulation. A complementary bin-by-bin (0.25 Hz each) examination across the global frequency spectrum (1–50 Hz) revealed that this significant Time-by-Group interaction appeared selectively in the targeted alpha band (8–10.5 Hz; Figure 2A yellow line), confirming the specific modulation of alpha power. However, baselines for the two sessions did not differ in either group (*P* > 0.598), failing to indicate a long-term shift in alpha power (Figure 2B). Finally, there was no Session-by-Time-by-Group interaction (*P* = 0.268) such that the within-session effects did not change from the first to the last session. Overall, the results confirmed that α-tACS produced an immediate and specific increase in alpha power, lasting 30 min post-stimulation.

### tACS induced immediate, short-term and long-term increases in posterior ➔frontal alpha connectivity

**Fig. 3:**
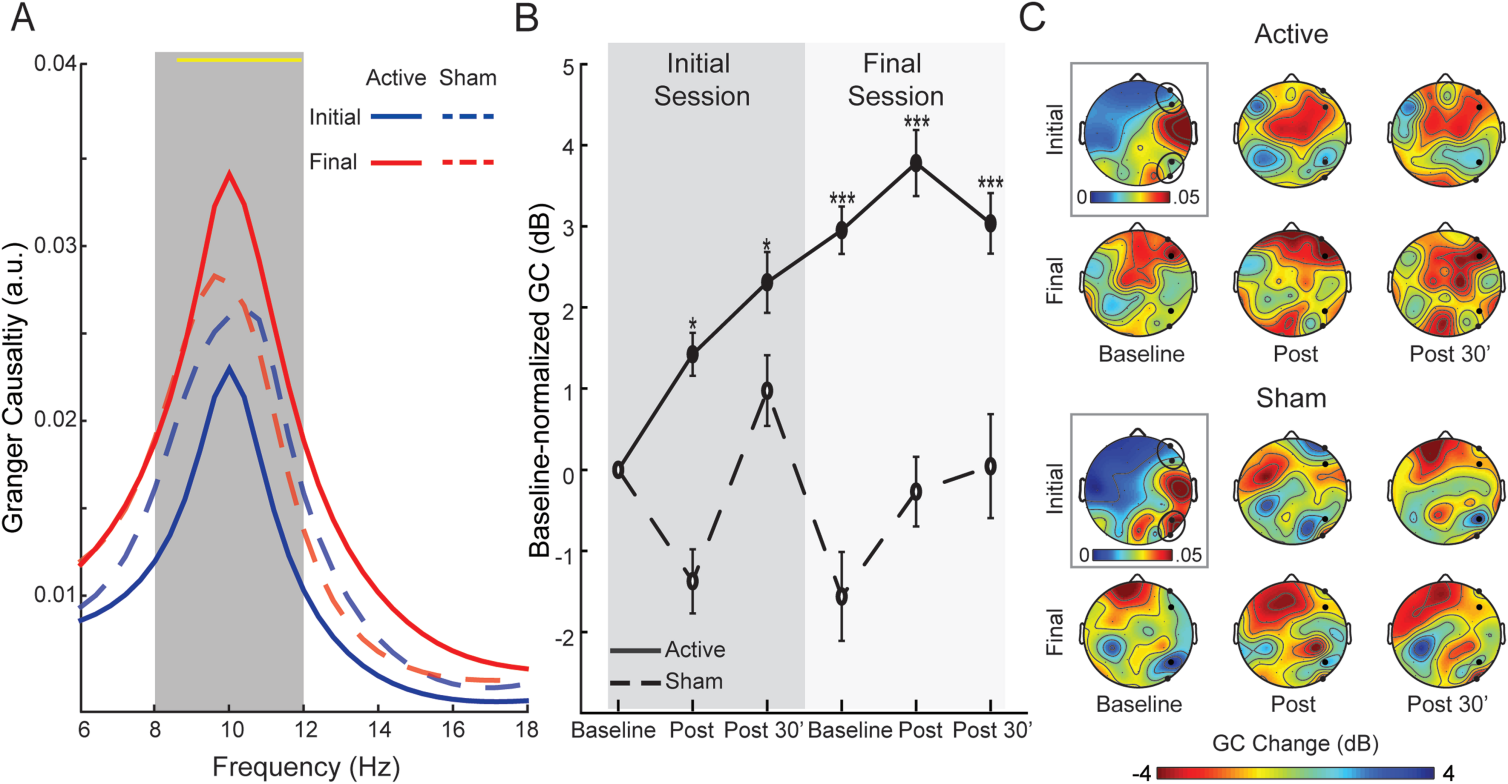
Changes in posterior ➔frontal GC. **(A)** Spectra of right-hemisphere posterior ➔frontal GC for each group (averaged across time for simplicity). The yellow line at the top of the waveforms indicates frequency bins (0.25 Hz each) showing a significant Session-by-Group interaction, which spans almost the entire alpha band (8.25–12 Hz) but no other frequency band, confirming the specific modulation of alpha-frequency GC. **(B)** Magnitudes of right posterior ➔frontal alpha GC, decibel-normalized to initial baseline, for each group. **(C)** Scalp topographical maps of alpha GC sent from right occipitoparietal electrodes at the initial baseline (surrounded by a black box and associated with its own color scale) and decibel-normalized changes at each assessment thereafter. Sending occipitoparietal and receiving frontal electrodes are bolded and encircled. Error bars = SEM. ^*^P < 0.05; ^**^P < 0.01; ^***^P < 0.005. All *P-*values are relative to the initial baseline. Note that the groups did not differ in the initial baseline values (*P* > 0.19).

A similar omnibus rANOVA of Site (right, left hemisphere), Session, Time, and Group on posterior ➔frontal alpha GC showed a significant four-way interaction of Site-by-Session-by-Time-by-Group (*F_1.98, 71.13_* = 4.18, *P* = 0.020, *η*_p_^2^ = 0.10). A follow-up ANOVA (Session-by-Time-by-Group) in the right hemisphere revealed a Session-by-Group interaction (*F_1, 36_* = 7.71, *P =* 0*.*009, *η*_p_^2^ = 0.18; Figure 3A), which was substantiated by a significant increase from the initial to the final session in the Active (*F_1, 20_* = 12.11, *P =* 0*.*002, *η*_p_^2^ = 0.38) but not Sham (*P =* 0*.*487) group. Furthermore, as illustrated in Figure 3B, relative to the initial baseline, GC at all subsequent assessments was augmented in the Active group (*t*’s < −2.28, *P* < 0.05 FDR corrected) but not in the Sham group (*P* > 0.190), reflecting immediate and sustained enhancement of connectivity via tACS. A bin-by-bin (0.25 Hz each) survey across the global frequency spectrum showed that this Session-by-Group interaction spanned almost the entire alpha band (8.25–12 Hz) but no other frequency band, supporting specific modulation of alpha-frequency GC. Importantly, the Active group (but not the Sham group, *P =* 0*.*225) demonstrated a significant baseline shift from the initial to the final session (*F_1, 20_* = 18.46, *P* = 0.0004, *η*_p_^2^ = 0.48), highlighting a long-term effect of tACS. Finally, there was no Session-by-Time-by-Group interaction (*P* = 0.147) such that the within-session effects did not change with repeated stimulation.

A similar follow-up ANOVA in the left hemisphere showed an interaction of Session-by-Time-by-Group (*F_1.99, 71.58_* = 2.84, *P* = 0.065, *η*_p_^2^ = 0.07). However, this effect did not lend support to the notion of increased tACS potency over repeated stimulation. The three-way interaction was substantiated by an interaction of Session-by-Time in the Active (*F_1.99, 39.81_* = 3.13, *P* = 0.055, *η*_p_^2^ = 0.14) but not Sham (*P* = 0.363) group. Specifically, the Active group exhibited an effect of Time in the initial session (*F_1.96, 39.28_* = 3.87, *P* = 0.030, *η*_p_^2^ = 0.16) with a trend of immediate alpha GC increase (*P* = 0.065) and no change 30 min later (*P* = 0.504), in contrast to no effect of Time in the final session (*P* = 0.730). Finally, there was no baseline shift in the Active (*vs* Sham) group (*P* = 0.367), suggesting no lasting effect of stimulation in the left hemisphere.

### tACS reduced anxious arousal

**Fig. 4:**
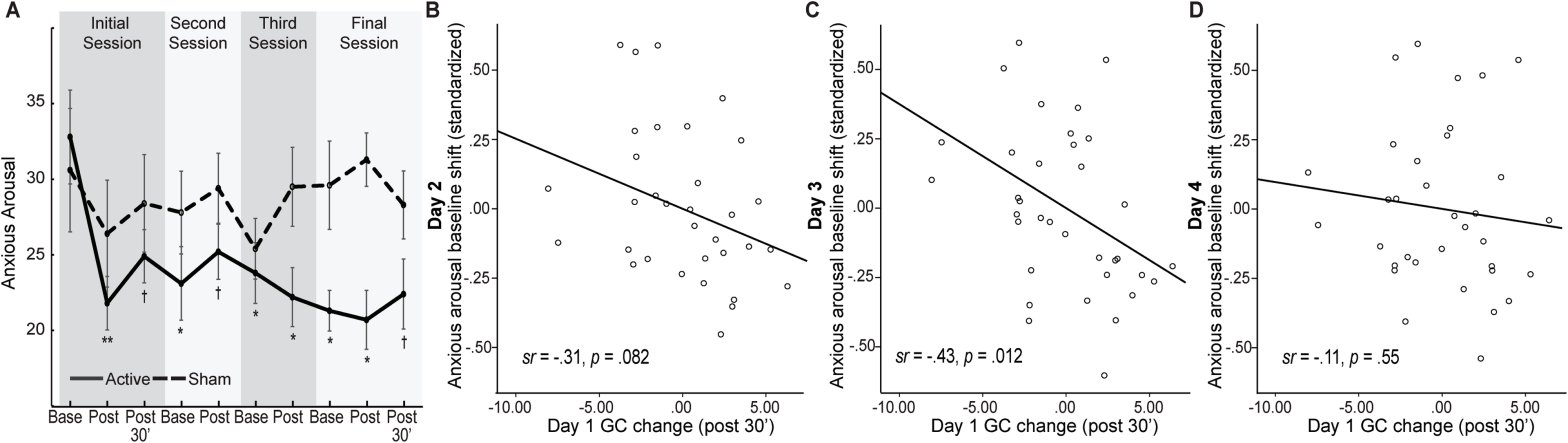
Anxious arousal outcome. **(A)** Anxious arousal ratings for each group at each assessment over the four sessions/days. **(B–D)** Day 1 (initial session) GC change at post-30 min predicted anxious arousal baseline shift (after controlling for individual differences in pre-study trait anxiety/BIS scores) on Day 2 (second session; B) and Day 3 (third session; C) but the association greatly declined on Day 4 (final session; D). Error bars = adjusted standard error (SEE). ^*^P < 0.05; ^**^P < 0.01; ^†^*P* < 0.1. All *P-*values are relative to the initial baseline. The groups did not differ in the initial baseline (*P* = 0.718).

An omnibus rANOVA of Session, Time, and Group on anxious arousal ratings revealed a main effect of Group (*F_1, 32_* = 4.64, *P* = 0.039, *η*_p_^2^ = 0.13); following the initial baseline, the two groups diverged in anxious arousal ratings, with the Active group showing consistently lowered ratings relative to initial baseline (*P* < 0.05 FDR corrected), while no change was observed in the Sham group (*P* > 0.317; Figure 4A). Importantly, the Active group, but not Sham (*P* = 0.607) group, demonstrated sustained baseline shifts across sessions (*F_2.60, 41.62_ =* 4.76, *P =* 0*.*008, *η*_p_^2^ = 0.23), such that, relative to the initial baseline on Day 1, the anxious arousal baseline was consistently lowered on Day 2 (*P* = 0.003), Day 3 (*P* = 0.021) and Day 4 (*P* = 0.005; Figure 4A). Therefore, tACS led to a consistent and lasting reduction in anxious arousal.

Next, to test whether alpha GC enhancement could underpin sustained anxiety reduction, we examined whether the right-hemisphere GC increase on Day 1 (post-30 min) could predict anxiety baseline shifts on the following days (relative to the Day 1 baseline). We observed a marginal effect of GC (*F*_1,32_ = 3.55, *P* = 0.069, *ηp^2^* = 0.10), which was further qualified by a trending Session-by-GC interaction (*F*_1.93, 62.89_ = 2.80, *P* = 0.071, *ηp^2^* = 0.08), suggesting a temporal progression in the association between GC and anxious arousal. Follow-up regression tests for each session revealed that Day 1 GC enhancement predicted reduction in baseline anxious arousal marginally on Day 2 (*sr* = −0.27, *P* = 0.082) and significantly on Day 3 (*sr* = −0.41, *P* = 0.012, *P* < 0.05 FDR corrected), but not on Day 4 (*sr* = −0.10, *P* = 0.539; Figure 4B–D)*.*

### tACS increased perceived pleasantness of sensory stimuli

#### Auditory stimuli

**Fig. 5:**
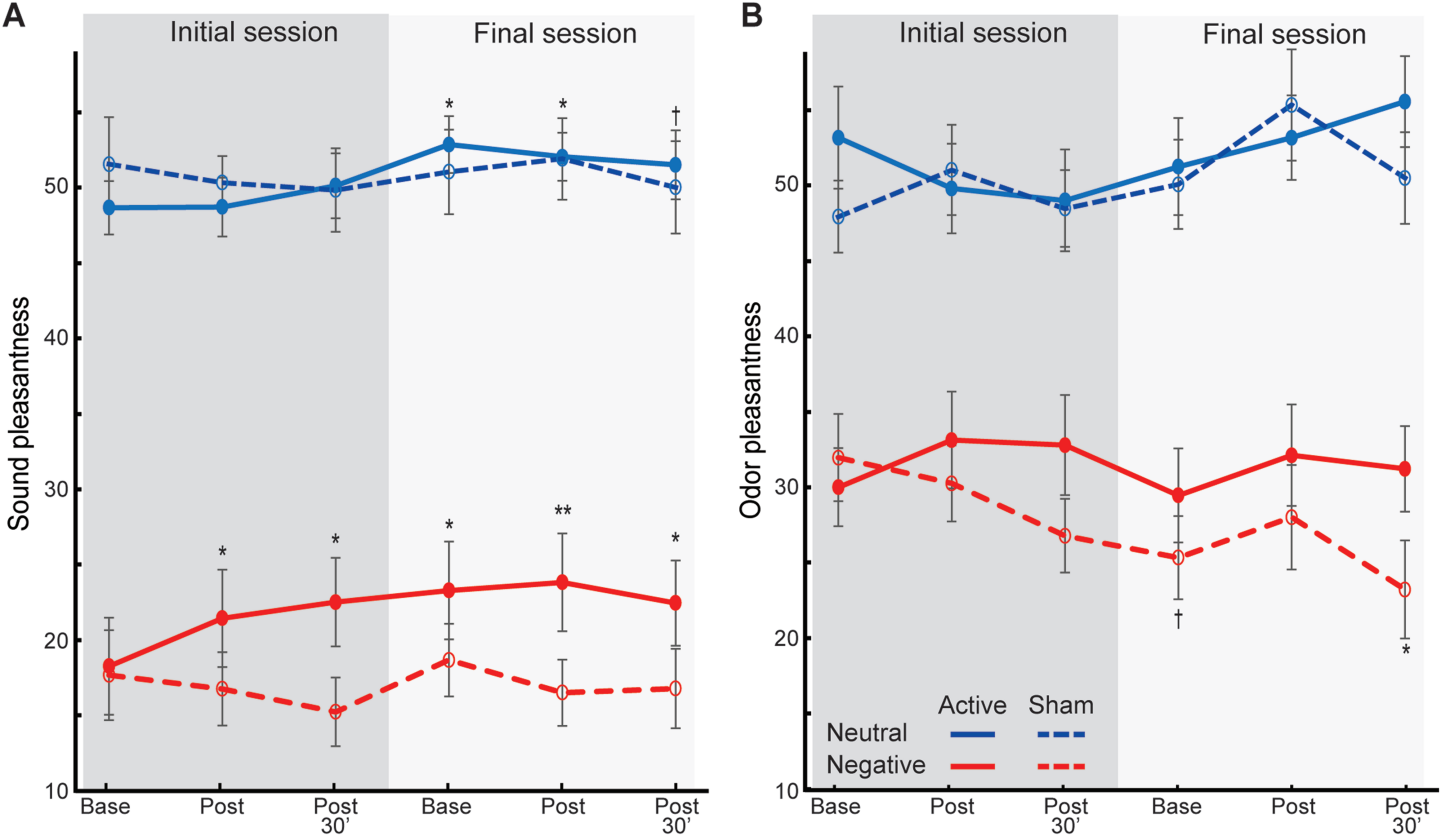
Pleasantness ratings of neutral and negative. **(A)** sounds and **(B)** odors. Neutral valence = 50. Error bars = SEM. ^*^P < 0.05; ^**^P < 0.01; ^†^*P* < 0.1. All *P-*values are relative to the initial baseline. The groups did not differ in the initial baseline ratings (*P* > 0.215).

An omnibus rANOVA (Valence, Intensity, Session, Time, and Group) on pleasantness ratings of auditory stimuli revealed a main effect of Group (Active > Sham; *F_1, 32_* = 4.19, *P =* 0*.*049, *η*_p_^2^ = 0.12), which was further characterized by a Session-by-Time-by-Group interaction (*F_1.57, 50.17_* = 3.84, *P =* 0*.*038, *η*_p_^2^ = 0.11; Figure 5A). However, this effect did not clearly support increased potency of tACS on affective perception. Follow-up tests for each session showed an interaction of Time-by-Group in the initial (*F_1.70, 54.28_* = 4.10, *P =* 0*.*028, *η*_p_^2^ = 0.11) but not final (*P =* 0*.*707) session. That is, following the initial tACS (but not sham stimulation, *P =* 0*.*644), auditory stimuli were perceived as more pleasant (*F_1.35, 21.59_* = 4.10, *P =* 0*.*045, *η*_p_^2^ = 0.20), immediately (*P* = 0.073) and 30 min (*P* = 0.040) after stimulation. Although no additional increases were seen after the final stimulation, final baseline ratings were significantly higher than initial baseline ratings (*t* = −2.43, *P* = 0.027) and remained elevated post-stimulation (immediately, *P* = 0.053; 30 min*, P =* 0.083), suggesting sustained increases in pleasantness at the final session. No such between- or within-session changes were seen in the Sham group (*P >* 0.572). Therefore, sound pleasantness ratings paralleled anxious arousal ratings, increasing after the initial tACS and persisting through the last assessment on Day 4.

#### Olfactory stimuli

A similar omnibus rANOVA on pleasantness ratings of olfactory stimuli revealed an interaction of Valence-by-Group (*F_1, 32_* = 8.51, *P =* 0*.*006, *η*_p_^2^ = 0.21), which was further qualified by a Valence-by-Time-by-Group interaction (*F_1.75, 55.95_* = 5.20, *P =* 0*.*011, *η*_p_^2^ = 0.14; Figure 5B). Follow-up ANOVAs revealed a Time-by-Group interaction for negative (*F_1.82, 58.10_* = 4.60, *P =* 0*.*017, *η*_p_^2^ = 0.13) but not neutral (*P =* 0*.*230) odor ratings. That is, the Active group showed trending increases in pleasantness of negative odors (*F*_1.88, 30.14_ = 3.15, *P* = 0.060, *ηp^2^* = 0.16), both immediately (*P* = 0.050) and, marginally, 30 min (*P* = 0.066) after stimulation, paralleled by opposing marginal decreases in pleasantness in the Sham group (*F*_1.72, 27.56_ = 3.32, *P* = 0.058, *ηp^2^* = 0.17). There were no Session-by-Time-by-Group interactions or baseline shifts (*P* > 0.202) to indicate between-session or long-term effects.

## Discussion

Combining multi-session α-tACS and multi-wave assessment in an experimental design, we demonstrated immediate and lasting neural and behavioral effects of tACS. α-tACS not only enhanced posterior ➔frontal alpha-frequency connectivity (in the right hemisphere) immediately and 30 min post-stimulation but also shifted the connectivity baseline 24 h after the preceding stimulation, indicating long-term plasticity. In parallel, anxious arousal decreased and perceived pleasantness of auditory stimuli increased both acutely (immediately and 30 min) and 24 h after α-tACS. By contrast, local posterior alpha power showed transient (immediately and 30 min post-stimulation) increases only, as did perceived pleasantness of negative olfactory stimuli. That repeated stimulation failed to induce lasting alpha power enhancement underscores the conservation of local alpha oscillations, contrasting the long-range alpha connectivity that is adaptive to long-term modulation by tACS. Our double-blind, single-session replication study with random noise stimulation as the control condition not only replicated the effect of anxiety reduction but also ruled-out potential confounds of experimenter biases and random electrical stimulation (Supplementary data).

The acute (immediate and 30 min) enhancement of local alpha power was evident in both initial and final sessions, echoing previous reports of reliable α-tACS after-effects (Herrmann *et al.*, 2013; Neuling *et al.*, 2013; Vossen *et al.*, 2015). Furthermore, bin-by-bin examination across the entire frequency spectrum confirmed the specific modulation in the alpha band. In addition, the effect appeared largely in the lower alpha band (8–10.5 Hz), in line with prior findings of leftward shifting of alpha peaks by α-tACS (Kasten *et al.*, 2016) and a general trend of higher alpha power being associated with lower alpha peaks (Smit *et al.*, 2006). Transient alpha power enhancement following a single tACS session as previously reported (Kasten *et al.*, 2016) could be attributed to the insufficient stimulation dosage to induce lasting, late LTP to support long-term plasticity. That our protocol of multiple stimulation sessions still failed to generate lasting power increases refuted the account of insufficient dosage, especially in light of lasting alpha connectivity enhancement indicating that the current dosage was sufficient for long-term plasticity. Instead, the transient alpha power increase likely reflected neural entrainment that temporally synchronized occipitoparietal cortical oscillations to exogenous stimulation (Reato *et al.*, 2013; Helfrich *et al.*, 2014). Therefore, as tACS ceased, the highly integrated thalamo-cortical alpha pacemaker (Hughes and Crunelli, 2005; Bazanova and Vernon, 2014) might allow thalamic inputs to restore cortical alpha oscillations to endogenous levels. Overall, the restoration of alpha power, despite repeated stimulation, accentuated the resistance of cortical alpha oscillators to long-term changes. This intrinsic regulation of resting-state alpha oscillations in healthy participants thus implicated a conserved, closed-loop (potentially encapsulated) system of alpha oscillators, critical for preserving neural homeostasis and mental stability (Sterling, 2014).

By contrast, alpha-frequency posterior ➔frontal connectivity exhibited both immediate and lasting changes, representing some of the first empirical evidence for long-term neural effects of tACS. The immediate effects replicated a recent report of immediate increases in resting-state connectivity between parietal and frontal cortices following α-tACS (Cabral-Calderin *et al.*, 2016). The lasting enhancement in alpha connectivity, in contrast to alpha power, ruled out the possibility that augmented alpha connectivity merely resulted from a potentiated posterior alpha sender (via enhanced alpha synchrony in the occipitoparietal cortex) or a higher signal-to-noise ratio of scalp EEG at the assessment on Day 4. Rather, the evidence implicated mechanisms beyond local neuronal synchrony, in favor of the interpretation of improved efficiency in alpha projections across large-scale networks, such that with alpha power at the sender remaining constant, directed transmission of alpha oscillations to the distant receiver was intensified.

This improved efficiency in posterior ➔frontal alpha transmission was likely mediated by synaptic plasticity induced by tACS, via mechanisms such as STDP and LTP (Frohlich and McCormick, 2010; Reato *et al.*, 2010; Zaehle *et al.*, 2010; Polanía *et al.*, 2012; Vossen *et al.*, 2015), which is known to be conducive to enduring Hebbian synaptic plasticity and lasting increases in cortico-cortical oscillatory connectivity (Clopath *et al.*, 2010; Siegel *et al.*, 2012). Since such inter-cortical connectivity (driven by cortical alpha generators; Rockland and Virga, 1989; Bollimunta *et al.*, 2008) operates outside the relatively encapsulated thalamo-cortical loop that constrains local cortical oscillations, its plasticity can outlast the effect of local alpha entrainment. This account concurs with the notion that tACS directly modifies interactions across proximal and distant neurons to the extent that the dynamics of macroscopic, global networks are altered (Frohlich and McCormick, 2010; Reato *et al.*, 2010). Here, given the role of alpha oscillations in long-range interactions, α-tACS would generate pronounced connectivity plasticity in long-range pathways. In particular, the posterior ➔frontal alpha connectivity exhibited maximal gain, akin to its dominance in resting long-range interactions and, critically, tACS stimulation at the posterior site. Connectivity in the opposite direction (frontal ➔posterior) failed to show acute or delayed effects (*P* > 0.16). This highlights the importance of computing directional measures of connectivity (e.g. GC) such that directional effects would not be obscured by non-directional measures of connectivity. In addition, to the extent that GC can be influenced by signal power, the fact that GC increases persisted through Day 4 when power had reversed to the baseline largely mitigated this concern.

Paralleling alpha posterior ➔frontal connectivity increases, anxious arousal decreased both immediately and 24 h after tACS, which persisted across the 4 day period, highlighting reliable and robust effects on anxiety. These anxiety effects outlasted local alpha power enhancement and so were likely underpinned by strengthened posterior ➔frontal alpha connectivity, which presumably modulated large-scale RSNs, especially the DMN and SN that are closely linked to alpha oscillations (Laufs *et al.*, 2003; Buzsáki and Draguhn, 2004; Mantini *et al.*, 2007; Sadaghiani *et al.*, 2010; Mo *et al.*, 2013; Zhan *et al.*, 2014; Tang *et al.*, 2017a) and play critical roles in emotion and arousal (Raichle *et al.*, 2001; Sridharan *et al.*, 2008). The non-clinical nature, and relatively small size (for correlational effects), of the current sample may have occluded the full observation of previously demonstrated direct links between alpha connectivity and hyperarousal in a largely clinical sample (Clancy *et al.*, 2017). However, the fact that neural effects preceded lasting anxiety changes underscores neural plasticity that develops and consolidates over time, giving rise to enduring amelioration of anxiety and related behavioral metrics. Nevertheless, this alpha connectivity enhancement failed to predict anxiety reduction on Day 4, suggesting that neural effects of a single tACS would not sustain prolonged behavioral changes and that multiple sessions would be needed for enduring behavioral effects. Future research with daily resting-state EEG recordings and follow-up assessments (e.g. at 1 week post-treatment) would delineate detailed temporal profiles of neural changes and related behavioral impacts*.*

Anxiety is often associated with negative stimulus perception (Krusemark and Li, 2011; Forscher and Li, 2012; Krusemark and Li, 2012). Auditory processing is particularly sensitive to states of anxious arousal (Grillon *et al.*, 1991; Braff *et al.*, 2001). In addition, alpha oscillations are known to modulate sensory processing and perception (Palva and Palva, 2007; Foxe and Snyder, 2011; Klimesch *et al.*, 2011; Lou *et al.*, 2014) such that altered alpha activity via α-tACS could further mitigate aversive responses to auditory stimuli by modifying auditory processing. Indeed, immediate and lasting increases in perceived pleasantness of sounds (especially negative sounds) coincided with anxious arousal reduction and alpha connectivity enhancement, providing additional support to our hypotheses. Interestingly, perceived pleasantness of negative odors increased in a transient manner while lasting increases emerged in the auditory domain*.* Unlike the auditory system that is neocortical and has direct and close connections with the targeted visual cortex (Falchier *et al.*, 2002; Kayser *et al.*, 2008; Novak *et al.*, 2015), the olfactory system is largely subcortical or paleocortical without direct connections with visual cortex (Carmichael *et al.*, 1994; Haberly, 2001; Krusemark *et al.*, 2013). As such, olfactory perception may be relatively impervious to alpha purtabations with the transient changes (for negative odors) presumably reflecting brief adaptation to internal state changes because of tACS, as a phenomenon of olfactory alliesthesia (Krusemark *et al.*, 2013).

Finally, we explored the possibility of intensified potency of tACS effects over repeated sessions. While there were hints of greater within-session effects on Day 4 relative to Day 1 (Figures 2 and 3), three-way interactions of Session, Time, and Group failed to support this possibility. We surmise that these null effects implicated a biological ceiling among healthy individuals, especially considering the already elevated baselines (for GC connectivity) on Day 4. Similarly, in contrast to clinical patients in a clinical trial, our participants in this proof-of-concept study had normal levels of baseline anxious arousal (mean scores 32/100), leaving only modest room for anxiety reduction. Nonetheless, anxiety reduced by 34% after tACS on Day 1 and maintained throughout the study, resisting the natural regression to the baseline (as seen in the Sham group). That said, the possibility of tACS potency intensification among patients with reduced alpha activity and greater anxious arousal warrants investigation through double-blind clinical trials, which could provide useful dosage guidance in clinical applications.

Increasing recognition of aberrant oscillatory dynamics in the pathology of various neuropsychiatric disorders has spawned strong interest in directly modulating endogenous oscillations (Schnitzler and Gross, 2005; Uhlhaas and Singer, 2012; Basar, 2013). Meanwhile, network views of psychopathology have gained rapid traction in recent years, advancing conceptualization of mental disorders from local neural aberrations to large-scale circuit anomalies (Kaiser *et al.*, 2015; Clancy *et al.*, 2017; McTeague *et al.*, 2017). In fact, while our laboratory demonstrated both reduced posterior alpha power and posterior ➔frontal alpha connectivity in PTSD, we found that only the latter was directly coupled with symptom severity (Clancy *et al.*, 2017). Therefore, the ability of tACS to induce lasting plasticity in long-range connectivity makes this neuromodulatory technique especially advantageous for treating disorders rooted in pathological oscillatory networks. While meditation and neurofeedback can modulate RSNs and reduce anxiety and arousal, along with alpha power enhancement (Hammond, 2005; Cahn and Polich, 2006; Ros *et al.*, 2013; Fan *et al.*, 2014; Tang *et al.*, 2017b), their long-term impact on alpha-frequency connectivity remain unclear. In this regard, we call for attention to oscillatory connectivity modification and network adaptation in future investigation of all forms of neuromodulation.

## Funding

This research was supported by the National Institute of Mental Health (R01MH093413 to W.L.) and (R21MH112206 to M.D.) and the FSU Chemical Senses Training Grant Award (T32DC000044 to K.C.) from the National Institutes of Health/National Institute on Deafness and Other Communication Disorders.

## Supplementary Material

Supplementary Data

## References

[ref1] Abend R., Jalon I., Gurevitch G., et al. (2016). Modulation of fear extinction processes using transcranial electrical stimulation. Translational Psychiatry, 6(10), e913. 27727241 10.1038/tp.2016.197 PMC5315554

[ref2] Abraham W.C. (2003). How long will long-term potentiation last? Philosophical Transactions of the Royal Society of London. Series B, Biological Sciences, 358(1432), 735–44. 12740120 10.1098/rstb.2002.1222 PMC1693170

[ref3] Adler L.E., Pang K., Gerhardt G., Rose G.M. (1988). Modulation of the gating of auditory evoked potentials by norepinephrine: pharmacological evidence obtained using a selective neurotoxin. Biological Psychiatry, 24(2), 179–90. 3390498 10.1016/0006-3223(88)90273-9

[ref4] Alagapan S., Schmidt S.L., Lefebvre J., Hadar E., Shin H.W., Fröhlich F. (2016). Modulation of cortical oscillations by low-frequency direct cortical stimulation is state-dependent. PLoS Biology, 14(3), e1002424. 27023427 10.1371/journal.pbio.1002424 PMC4811434

[ref5] Alvarez R.P., Kirlic N., Misaki M., et al. (2015). Increased anterior insula activity in anxious individuals is linked to diminished perceived control. Translational Psychiatry, 5, e591. 26125154 10.1038/tp.2015.84 PMC4490294

[ref6] Antal A., Paulus W. (2013). Transcranial alternating current stimulation (tACS). Frontiers in Human Neuroscience, 7, 317. 23825454 10.3389/fnhum.2013.00317 PMC3695369

[ref7] Basar E. (2013). Brain oscillations in neuropsychiatric disease. Dialogues in Clinical Neuroscience, 15(3), 291–300. 24174901 10.31887/DCNS.2013.15.3/ebasar PMC3811101

[ref8] Başar E., Başar-Eroglu C., Karakaş S., Schürmann M. (2001). Gamma, alpha, delta, and theta oscillations govern cognitive processes. International Journal of Psychophysiology, 39(2), 241–8. 11163901 10.1016/s0167-8760(00)00145-8

[ref9] Bazanova O.M., Vernon D. (2014). Interpreting EEG alpha activity. Neuroscience and Biobehavioral Reviews, 44, 94–110. 23701947 10.1016/j.neubiorev.2013.05.007

[ref10] Bollimunta A., Chen Y., Schroeder C.E., Ding M. (2008). Neuronal mechanisms of cortical alpha oscillations in awake-behaving macaques. The Journal of Neuroscience, 28(40), 9976–88. 18829955 10.1523/JNEUROSCI.2699-08.2008 PMC2692971

[ref11] Braff D.L., Geyer M.A., Swerdlow N.R. (2001). Human studies of prepulse inhibition of startle: normal subjects, patient groups, and pharmacological studies. Psychopharmacology, 156(2–3), 234–58. 11549226 10.1007/s002130100810

[ref13] Buzsáki G., Draguhn A. (2004). Neuronal oscillations in cortical networks. Science, 304(5679), 1926–9. 15218136 10.1126/science.1099745

[ref14] Buzsáki G., Logothetis N., Singer W. (2013). Scaling brain size, keeping timing: evolutionary preservation of brain rhythms. Neuron, 80(3), 751–64. 24183025 10.1016/j.neuron.2013.10.002 PMC4009705

[ref15] Cabral-Calderin Y., Williams K.A., Opitz A., Dechent P., Wilke M. (2016). Transcranial alternating current stimulation modulates spontaneous low frequency fluctuations as measured with fMRI. NeuroImage, 141, 88–107. 27393419 10.1016/j.neuroimage.2016.07.005

[ref16] Cahn B.R., Polich J. (2006). Meditation states and traits: EEG, ERP, and neuroimaging studies. Psychological Bulletin, 132(2), 180–211. 16536641 10.1037/0033-2909.132.2.180

[ref17] Carmichael S.T., Clugnet M.C., Price J.L. (1994). Central olfactory connections in the macaque monkey. The Journal of Comparative Neurology, 346(3), 403–34. 7527806 10.1002/cne.903460306

[ref18] Carver C.S., White T.L. (1994). Behavioral inhibition, behavioral activation, and affective responses to impending reward and punishment: the BIS/BAS scales. Journal of Personality and Social Psychology, 67(2), 319–33.

[ref19] Clancy K., Ding M., Bernat E., Schmidt N.B., Li W. (2017). Restless ‘rest’: intrinsic sensory hyperactivity and disinhibition in post-traumatic stress disorder. Brain, 140(7), 2041–50. 28582479 10.1093/brain/awx116 PMC6059177

[ref20] Clopath C., Büsing L., Vasilaki E., Gerstner W. (2010). Connectivity reflects coding: a model of voltage-based STDP with homeostasis. Nature Neuroscience, 13(3), 344–52. 20098420 10.1038/nn.2479

[ref21] Cohen M.R., Maunsell J.H. (2010). A neuronal population measure of attention predicts behavioral performance on individual trials. The Journal of Neuroscience, 30(45), 15241–53. 21068329 10.1523/JNEUROSCI.2171-10.2010 PMC3045704

[ref22] Cohen M.X. (2014). *Analyzing Neural Time Series Data: Theory and Practice*. London, England: MIT Press.

[ref23] Corbetta M., Shulman G.L. (2011). Spatial neglect and attention networks. Annual Review of Neuroscience, 34, 569–99. 10.1146/annurev-neuro-061010-113731 PMC3790661 21692662

[ref24] Ding M., Bressler S.L., Yang W., Liang H. (2000). Short-window spectral analysis of cortical event-related potentials by adaptive multivariate autoregressive modeling: data preprocessing, model validation, and variability assessment. Biological Cybernetics, 83(1), 35–45. 10933236 10.1007/s004229900137

[ref25] Ding M., Chen Y., Bressler S.L. (2006). Granger causality: basic theory and application to neuroscience In: *Handbook of Time Series Analysis*, Weinheim: Wiley-VCH Verlag GmbH & Co. KGaA.

[ref26] Dugué L., Marque P., VanRullen R. (2011). The phase of ongoing oscillations mediates the causal relation between brain excitation and visual perception. The Journal of Neuroscience, 31(33), 11889–93. 21849549 10.1523/JNEUROSCI.1161-11.2011 PMC6623205

[ref27] Engel A.K., Fries P., Singer W. (2001). Dynamic predictions: oscillations and synchrony in top-down processing. Nature Reviews. Neuroscience, 2(10), 704–16. 11584308 10.1038/35094565

[ref28] Falchier A., Clavagnier S., Barone P., Kennedy H. (2002). Anatomical evidence of multimodal integration in primate striate cortex. The Journal of Neuroscience, 22(13), 5749–59. 12097528 10.1523/JNEUROSCI.22-13-05749.2002 PMC6758216

[ref29] Fan Y., Tang Y.Y., Tang R., Posner M.I. (2014). Short term integrative meditation improves resting alpha activity and stroop performance. Applied Psychophysiology and Biofeedback, 39(3–4), 213–7. 25253652 10.1007/s10484-014-9258-5

[ref30] Forscher E.C., Li W. (2012). Hemispheric asymmetry and visuo-olfactory integration in perceiving subthreshold (micro) fearful expressions. The Journal of Neuroscience, 32(6), 2159–65. 22323728 10.1523/JNEUROSCI.5094-11.2012 PMC3568979

[ref31] Foxe J.J., Snyder A.C. (2011). The role of alpha-band brain oscillations as a sensory suppression mechanism during selective attention. Frontiers in Psychology, 2, 154. 21779269 10.3389/fpsyg.2011.00154 PMC3132683

[ref32] Fregni F., Otachi P.T., Do Valle A., et al. (2006). A randomized clinical trial of repetitive transcranial magnetic stimulation in patients with refractory epilepsy. Annals of Neurology, 60(4), 447–55. 17068786 10.1002/ana.20950

[ref33] Fröhlich F. (2015). Experiments and models of cortical oscillations as a target for noninvasive brain stimulation. Progress in Brain Research, 222, 41–73. 26541376 10.1016/bs.pbr.2015.07.025

[ref34] Fröhlich F., McCormick D.A. (2010). Endogenous electric fields may guide neocortical network activity. Neuron, 67(1), 129–43. 20624597 10.1016/j.neuron.2010.06.005 PMC3139922

[ref35] Gath I., Lehmann D., Bar-On E. (1983). Fuzzy clustering of EEG signal and vigilance performance. The International Journal of Neuroscience, 20(3–4), 303–12. 6668126 10.3109/00207458308986584

[ref36] Geweke J. (1982). Measurement of linear dependence and feedback between multiple time series. Journal of the American Statistical Association, 77(378), 304–13.

[ref37] Grillon C., Ameli R., Woods S.W., Merikangas K., Davis M. (1991). Fear-potentiated startle in humans: effects of anticipatory anxiety on the acoustic blink reflex. Psychophysiology, 28(5), 588–95. 1758934 10.1111/j.1469-8986.1991.tb01999.x

[ref38] Haberly L.B. (2001). Parallel-distributed processing in olfactory cortex: new insights from morphological and physiological analysis of neuronal circuitry. Chemical Senses, 26(5), 551–76. 11418502 10.1093/chemse/26.5.551

[ref39] Hammond D.C. (2005). Neurofeedback with anxiety and affective disorders. Child and Adolescent Psychiatric Clinics of North America, 14(1), 105–23 vii. 15564054 10.1016/j.chc.2004.07.008

[ref40] Helfrich R.F., Schneider T.R., Rach S., Trautmann-Lengsfeld S.A., Engel A.K., Herrmann C.S. (2014). Entrainment of brain oscillations by transcranial alternating current stimulation. Current Biology, 24(3), 333–9. 24461998 10.1016/j.cub.2013.12.041

[ref41] Herrmann C.S., Rach S., Neuling T., Strüber D. (2013). Transcranial alternating current stimulation: a review of the underlying mechanisms and modulation of cognitive processes. Frontiers in Human Neuroscience, 7, 279. 23785325 10.3389/fnhum.2013.00279 PMC3682121

[ref42] Hillebrand A., Tewarie P., Dellen E. van, et al. (2016). Direction of information flow in large-scale resting-state networks is frequency-dependent. Proceedings of the National Academy of Sciences of the United States of America, 113(14), 3867–72. 27001844 10.1073/pnas.1515657113 PMC4833227

[ref43] Huang Y.Z., Lu M.K., Antal A., et al. (2017). Plasticity induced by non-invasive transcranial brain stimulation: a position paper. *Clinical Neurophysiology*, 128(11), 2318–2329. 29040922 10.1016/j.clinph.2017.09.007

[ref44] Hughes S.W., Crunelli V. (2005). Thalamic mechanisms of EEG alpha rhythms and their pathological implications. The Neuroscientist, 11(4), 357–72. 16061522 10.1177/1073858405277450

[ref45] Johnson E.L., Dewar C.D., Solbakk A.K., Endestad T., Meling T.R., Knight R.T. (2017). Bidirectional frontoparietal oscillatory systems support working memory. Current Biology, 27(12), 1829–35. 28602658 10.1016/j.cub.2017.05.046 PMC5546232

[ref46] Kaiser R.H., Andrews-Hanna J.R., Wager T.D., Pizzagalli D.A. (2015). Large-scale network dysfunction in major depressive disorder: a meta-analysis of resting-state functional connectivity. JAMA Psychiatry, 72(6), 603–11. 25785575 10.1001/jamapsychiatry.2015.0071 PMC4456260

[ref47] Kasten F.H., Dowsett J., Herrmann C.S. (2016). Sustained aftereffect of α-tACS lasts up to 70 min after stimulation. Frontiers in Human Neuroscience, 10, 245. 27252642 10.3389/fnhum.2016.00245 PMC4879138

[ref48] Kayser C., Petkov C.I., Logothetis N.K. (2008). Visual modulation of neurons in auditory cortex. Cerebral Cortex, 18(7),1560–74. 18180245 10.1093/cercor/bhm187

[ref49] Klimesch W. (1999). EEG alpha and theta oscillations reflect cognitive and memory performance: a review and analysis. Brain Research. Brain Research Reviews, 29(2–3), 169–95. 10209231 10.1016/s0165-0173(98)00056-3

[ref50] Klimesch W. (2012). α-band oscillations, attention, and controlled access to stored information. Trends in Cognitive Sciences, 16(12), 606–17. 23141428 10.1016/j.tics.2012.10.007 PMC3507158

[ref51] Klimesch W., Fellinger R., Freunberger R. (2011). Alpha oscillations and early stages of visual encoding. Frontiers in Psychology, 2, 118. 21687470 10.3389/fpsyg.2011.00118 PMC3108577

[ref52] Krusemark E.A., Li W. (2011). Do all threats work the same way? Divergent effects of fear and disgust on sensory perception and attention. The Journal of Neuroscience, 31(9), 3429–34. 21368054 10.1523/JNEUROSCI.4394-10.2011 PMC3077897

[ref53] Krusemark E.A., Li W. (2012). Enhanced olfactory sensory perception of threat in anxiety: an event-related fMRI study. Chemosensory Perception, 5(1), 37–45. 22866182 10.1007/s12078-011-9111-7 PMC3410736

[ref54] Krusemark E.A., Novak L.R., Gitelman D.R., Li W. (2013). When the sense of smell meets emotion: anxiety-state-dependent olfactory processing and neural circuitry adaptation. The Journal of Neuroscience, 33(39), 15324–32. 24068799 10.1523/JNEUROSCI.1835-13.2013 PMC3782615

[ref55] Laufs H., Krakow K., Sterzer P., et al. (2003). Electroencephalographic signatures of attentional and cognitive default modes in spontaneous brain activity fluctuations at rest. Proceedings of the National Academy of Sciences of the United States of America, 100(19), 11053–8. 12958209 10.1073/pnas.1831638100 PMC196925

[ref56] Lou B., Li Y., Philiastides M.G., Sajda P. (2014). Prestimulus alpha power predicts fidelity of sensory encoding in perceptual decision making. NeuroImage, 87, 242–51. 24185020 10.1016/j.neuroimage.2013.10.041 PMC3946902

[ref57] Mantini D., Perrucci M.G., Del Gratta C., Romani G.L., Corbetta M. (2007). Electrophysiological signatures of resting state networks in the human brain. Proceedings of the National Academy of Sciences of the United States of America, 104(32), 13170–5. 17670949 10.1073/pnas.0700668104 PMC1941820

[ref58] Mathewson K.E., Gratton G., Fabiani M., Beck D.M., Ro T. (2009). To see or not to see: prestimulus alpha phase predicts visual awareness. The Journal of Neuroscience, 29(9), 2725–32. 19261866 10.1523/JNEUROSCI.3963-08.2009 PMC2724892

[ref59] McTeague L.M., Huemer J., Carreon D.M., Jiang Y., Eickhoff S.B., Etkin A. (2017). Identification of common neural circuit disruptions in cognitive control across psychiatric disorders. The American Journal of Psychiatry, 174(7), 676–85. 28320224 10.1176/appi.ajp.2017.16040400 PMC5543416

[ref60] Menon V., Uddin L.Q. (2010). Saliency, switching, attention and control: a network model of insula function. Brain Structure & Function, 214(5–6), 655–67. 20512370 10.1007/s00429-010-0262-0 PMC2899886

[ref61] Mitra P.P., Pesaran B. (1999). Analysis of dynamic brain imaging data. Biophysical Journal, 76(2), 691–708. 9929474 10.1016/S0006-3495(99)77236-X PMC1300074

[ref62] Mo J., Liu Y., Huang H., Ding M. (2013). Coupling between visual alpha oscillations and default mode activity. NeuroImage, 68, 112–8. 23228510 10.1016/j.neuroimage.2012.11.058 PMC3557590

[ref63] Moisa M., Polania R., Grueschow M., Ruff C.C. (2016). Brain network mechanisms underlying motor enhancement by transcranial entrainment of gamma oscillations. The Journal of Neuroscience, 36(47), 12053–65. 27881788 10.1523/JNEUROSCI.2044-16.2016 PMC6604912

[ref64] Neuling T., Rach S., Herrmann C.S. (2013). Orchestrating neuronal networks: sustained after-effects of transcranial alternating current stimulation depend upon brain states. Frontiers in Human Neuroscience, 7, 161. 23641206 10.3389/fnhum.2013.00161 PMC3639376

[ref65] Neuling T., Wagner S., Wolters C.H., Zaehle T., Herrmann C.S. (2012). Finite-element model predicts current density distribution for clinical applications of tDCS and tACS. Frontiers in Psychiatry, 3, 83. 23015792 10.3389/fpsyt.2012.00083 PMC3449241

[ref66] Nolan H., Whelan R., Reilly R.B. (2010). FASTER: Fully Automated Statistical Thresholding for EEG artifact Rejection. Journal of Neuroscience Methods, 192(1), 152–62. 20654646 10.1016/j.jneumeth.2010.07.015

[ref67] Novak L.R., Gitelman D.R., Schuyler B., Li W. (2015). Olfactory-visual integration facilitates perception of subthreshold negative emotion. Neuropsychologia, 77, 288–97. 26359718 10.1016/j.neuropsychologia.2015.09.005 PMC4699288

[ref68] Nunez P.L., Srinivasan R., Westdorp A.F., et al. (1997). EEG coherency. I: statistics, reference electrode, volume conduction, Laplacians, cortical imaging, and interpretation at multiple scales. Electroencephalography and Clinical Neurophysiology, 103(5), 499–515. 9402881 10.1016/s0013-4694(97)00066-7

[ref69] Palva S., Palva J.M. (2007). New vistas for alpha-frequency band oscillations. Trends in Neurosciences, 30(4), 150–8. 17307258 10.1016/j.tins.2007.02.001

[ref70] Perrin F., Pernier J., Bertrand O., Echallier J.F. (1989). Spherical splines for scalp potential and current density mapping. Electroencephalography and Clinical Neurophysiology, 72(2), 184–7. 2464490 10.1016/0013-4694(89)90180-6

[ref71] Peterson A., Thome J., Frewen P., Lanius R.A. (2014). Resting-state neuroimaging studies: a new way of identifying differences and similarities among the anxiety disorders? Canadian Journal of Psychiatry, 59(6), 294–300. 25007403 10.1177/070674371405900602 PMC4079145

[ref72] Polanía R., Nitsche M.A., Korman C., Batsikadze G., Paulus W. (2012). The importance of timing in segregated theta phase-coupling for cognitive performance. Current Biology, 22(14), 1314–8. 22683259 10.1016/j.cub.2012.05.021

[ref73] Raichle M.E., MacLeod A.M., Snyder A.Z., Powers W.J., Gusnard D.A., Shulman G.L. (2001). A default mode of brain function. Proceedings of the National Academy of Sciences of the United States of America, 98(2), 676–82. 11209064 10.1073/pnas.98.2.676 PMC14647

[ref74] Rajagovindan R., Ding M. (2011). From prestimulus alpha oscillation to visual-evoked response: an inverted-U function and its attentional modulation. Journal of Cognitive Neuroscience, 23(6), 1379–94. 20459310 10.1162/jocn.2010.21478

[ref75] Reato D., Rahman A., Bikson M., Parra L.C. (2010). Low-intensity electrical stimulation affects network dynamics by modulating population rate and spike timing. The Journal of Neuroscience, 30(45), 15067–79. 21068312 10.1523/JNEUROSCI.2059-10.2010 PMC3500391

[ref76] Reato D., Rahman A., Bikson M., Parra L.C. (2013). Effects of weak transcranial alternating current stimulation on brain activity—a review of known mechanisms from animal studies. Frontiers in Human Neuroscience, 7, 687. 24167483 10.3389/fnhum.2013.00687 PMC3805939

[ref77] Reis J., Schambra H.M., Cohen L.G., et al. (2009). Noninvasive cortical stimulation enhances motor skill acquisition over multiple days through an effect on consolidation. Proceedings of the National Academy of Sciences of the United States of America, 106(5), 1590–5. 19164589 10.1073/pnas.0805413106 PMC2635787

[ref78] Rockland K.S., Virga A. (1989). Terminal arbors of individual ‘feedback’ axons projecting from area V2 to V1 in the macaque monkey: a study using immunohistochemistry of anterogradely transported *Phaseolus vulgaris*-leucoagglutinin. The Journal of Comparative Neurology, 285(1), 54–72. 2754047 10.1002/cne.902850106

[ref79] Ros T., Théberge J., Frewen P.A., et al. (2013). Mind over chatter: plastic up-regulation of the fMRI salience network directly after EEG neurofeedback. NeuroImage, 65, 324–35. 23022326 10.1016/j.neuroimage.2012.09.046 PMC5051955

[ref80] Sadaghiani L., Gleeson H.B., Youde S., Waddington R.J., Lynch C.D., Sloan A.J. (2016). Growth factor liberation and DPSC response following dentine conditioning. Journal of Dental Research, 95(11), 1298–307. 27307049 10.1177/0022034516653568

[ref81] Sadaghiani S., Scheeringa R., Lehongre K., Morillon B., Giraud A.L., Kleinschmidt A. (2010). Intrinsic connectivity networks, alpha oscillations, and tonic alertness: a simultaneous electroencephalography/functional magnetic resonance imaging study. The Journal of Neuroscience, 30(30), 10243–50. 20668207 10.1523/JNEUROSCI.1004-10.2010 PMC6633365

[ref82] Sadaghiani S., Scheeringa R., Lehongre K., et al. (2012). Alpha-band phase synchrony is related to activity in the fronto-parietal adaptive control network. The Journal of Neuroscience, 32(41), 14305–10. 23055501 10.1523/JNEUROSCI.1358-12.2012 PMC4057938

[ref83] Schnitzler A., Gross J. (2005). Normal and pathological oscillatory communication in the brain. Nature Reviews. Neuroscience, 6(4), 285–96. 15803160 10.1038/nrn1650

[ref84] Siegel M., Donner T.H., Engel A.K. (2012). Spectral fingerprints of large-scale neuronal interactions. Nature Reviews. Neuroscience, 13(2), 121–34. 22233726 10.1038/nrn3137

[ref85] Smit C.M., Wright M.J., Hansell N.K., Geffen G.M., Martin N.G. (2006). Genetic variation of individual alpha frequency (IAF) and alpha power in a large adolescent twin sample. International Journal of Psychophysiology, 61(2), 235–43. 16338015 10.1016/j.ijpsycho.2005.10.004

[ref86] Sridharan D., Levitin D.J., Menon V. (2008). A critical role for the right fronto-insular cortex in switching between central-executive and default-mode networks. Proceedings of the National Academy of Sciences of the United States of America, 105(34), 12569–74. 18723676 10.1073/pnas.0800005105 PMC2527952

[ref87] Sripada R.K., King A.P., Welsh R.C., et al. (2012). Neural dysregulation in posttraumatic stress disorder: evidence for disrupted equilibrium between salience and default mode brain networks. Psychosomatic Medicine, 74(9), 904–11. 23115342 10.1097/PSY.0b013e318273bf33 PMC3498527

[ref88] Sterling P. (2014). Homeostasis *vs* allostasis: implications for brain function and mental disorders. JAMA Psychiatry, 71(10), 1192–3. 25103620 10.1001/jamapsychiatry.2014.1043

[ref89] Tang A. C., Sutherland M. T., Sun P., et al. (2007). Top-down versus bottom-up processing in the human brain: distinct directional influences revealed by integrating SOBI and Granger causality. In: Davies M.E., James C.J., Abdallah S.A., Plumbey M.D., editors. *Independent Component Analysis and Signal Separation*, 7th International Conference, ICA 2007, London, UK, 9 to 12 September 2007, Berlin, Heidelberg: Springer Berlin Heidelberg.

[ref90] Tang W., Liu H., Douw L., et al. (2017a). Dynamic connectivity modulates local activity in the core regions of the default-mode network. Proceedings of the National Academy of Sciences of the United States of America, 114(36), 9713–8. 28827337 10.1073/pnas.1702027114 PMC5594646

[ref91] Tang Y.Y., Tang Y., Tang R., Lewis-Peacock J.A. (2017b). Brief mental training reorganizes large-scale brain networks. Frontiers in Systems Neuroscience, 11, 6. 28293180 10.3389/fnsys.2017.00006 PMC5328965

[ref92] Thut G., Schyns P.G., Gross J. (2011). Entrainment of perceptually relevant brain oscillations by non-invasive rhythmic stimulation of the human brain. Frontiers in Psychology, 2, 170. 21811485 10.3389/fpsyg.2011.00170 PMC3142861

[ref93] Uhlhaas P.J., Singer W. (2012). Neuronal dynamics and neuropsychiatric disorders: toward a translational paradigm for dysfunctional large-scale networks. Neuron, 75(6), 963–80. 22998866 10.1016/j.neuron.2012.09.004

[ref94] Vossen A., Gross J., Thut G. (2015). Alpha power increase after transcranial alternating current stimulation at alpha frequency (alpha-tACS) reflects plastic changes rather than entrainment. Brain Stimulation, 8(3), 499–508. 25648377 10.1016/j.brs.2014.12.004 PMC4464304

[ref95] Wang C., Rajagovindan R., Han S.M., Ding M. (2016). Top-down control of visual alpha oscillations: sources of control signals and their mechanisms of action. Frontiers in Human Neuroscience, 10, 15. 26834601 10.3389/fnhum.2016.00015 PMC4718979

[ref96] Wolpe J., Lazarus A.A. (1966). Behaviour Therapy Techniques; A Guide to the Treatment of Neuroses, New York: Pergamon.

[ref97] Young C.B., Raz G., Everaerd D., et al. (2017). Dynamic shifts in large-scale brain network balance as a function of arousal. The Journal of Neuroscience, 37(2), 281–90. 28077708 10.1523/JNEUROSCI.1759-16.2016 PMC6596574

[ref98] Zaehle T., Rach S., Herrmann C.S. (2010). Transcranial alternating current stimulation enhances individual alpha activity in human EEG. PLoS One, 5(11), e13766. 21072168 10.1371/journal.pone.0013766 PMC2967471

[ref99] Zhan Z., Xu L., Zuo T., et al. (2014). The contribution of different frequency bands of fMRI data to the correlation with EEG alpha rhythm. Brain Research, 1543, 235–43. 24275197 10.1016/j.brainres.2013.11.016

