# Supplementary material for: Lasting connectivity increase and anxiety reduction via transcranial alternating current stimulation
Source: Soc Cogn Affect Neurosci. 2018 Oct 30;13(12):1305–16. doi: 10.1093/scan/nsy096 (PMC6277743; doi:10.1093/scan/nsy096)
Supplement: Supplementary Data [file nsy096_supp.zip › scan-18-051-File001.docx]

**Re: Revised manuscript (SCAN-18-051.R1) — “**Lasting connectivity increase and anxiety reduction via transcranial alternating current stimulation” **by Clancy et al.**

Dear Drs. Satpute and Lieberman,

Thank you for your email on 4th September 2018 regarding our manuscript. We are very happy to hear that all reviewers are favorable toward the research we are presenting. As you advised in your letter, we have responded to each of the points that were made. Modifications are highlighted in the text and reproduced here in corresponding responses (*italicized and highlighted in gray*).

In response to a specific issue you raised in the letter, we have provided additional clarification on the statistical analyses in the replication study. The *marginally* significant interaction effect the Reviewer raised was based on the two-tailed p value (*p* = .056). However, as we indicated in the manuscript, one-tailed tests were applied in the replication study given the nature of replication (and the hypothesized direction of the effect), and so the one-tailed p value (*p* = .028) should have been reported. We also note that the effect size in the replication study was quite large (*η*_p_² = .22), consistent with the effect size reported in the main findings (*η*_p_² = .23). In sum, the effect of tACS was statistically significant, and the replication can be deemed as successful.

We are grateful for your expertly handling of the manuscript. We are confident that the manuscript has been substantially strengthened as a result and should now be suitable for publication in *Social Cognitive and Affective Neuroscience.*

With best regards,

Kevin and Wen

**Responses to Reviewer #2**

We appreciate that the Reviewer sees the paper as “well written” with “an elegant multi-session design”. The Reviewer has made additional comments and suggestions, which we have carefully addressed in this second resubmission. We are very grateful for the further improvement in the manuscript as a result. Our responses are detailed below (Reviewer’s original comments appear in italics and quotations). Modifications are highlighted in the text and reproduced here in corresponding responses (*italicized and highlighted in gray*).

***Major issues:***

1. *“Replication study: while I appreciate the effort to partially replicate the current results, to me this additional study instilled moderate confidence in some of the findings (e.g. the reduction in anxious arousal due to alpha tACS), but left more questions open than answered.:*

*1a. Is the p value of Time x Group interaction on anxious arousal (p=.056) one or two tailed? Although p values are somewhat arbitrary, it seems misleading to report this as “significant”, rather than a trending pattern that resembled the prior results. Because the sample is relatively small (n=18), it is hard to know for sure if the p=.056 merely reflects a lack of power.”*

The p value (*p* = .056) was two-tailed. As we stated that one-tailed tests were used for the replication study given the hypothesized direction of effects, we should have reported the one-tailed (*p* = .028). According to the one-tailed p value, this interaction effect was statistically significant and indeed replicated our initial finding. We regret this oversight and have now presented the one-tailed *p* value in the resubmission. We also note that the effect size was quite large (*η*_p_² = .22), consistent with the effect size reported in the main findings (*η*_p_² = .23).

“ *1b. Further, where are the relevant statistics for Supplemental Figures 1 & 2, depicting tACS-dependent changes on alpha power and GC posterior frontal alpha connectivity, respectively?”*

In response to a request made by another Reviewer for us to present “all raw data”, we included Supplemental Figures 1 & 2, which were simply the expanded versions of Figs. 2A and 3A. As such, statistics related to these figures are already reported in the main text. We have included this explanation in the supplemental figure captions to improve clarity (pp. 38-39), as reproduced below.

p. 38: ***Figure S1.*** *Raw power spectra from occipitoparietal electrodes for all time points (pre-, post-, post-30 minutes stimulation) for the A) initial and B) final sessions from the main* *experiment.* *This figure represents an expansion of Figure 2A in the main text, which was collapsed across sessions to demonstrate the significant Time-by-Condition interaction.*

p. 39: ***Figure S2.*** *Raw spectra of right-hemisphere posterior*🡪*frontal Granger causality for each group for all time points (pre-, post-, post-30 minutes stimulation) for the A) initial and B) final sessions from the main experiment.* *This figure represents an expansion of Figure 3A in the main text, which was collapsed across time to demonstrate the significant Session-by-Condition interaction.*

*“In essence, to me, the results reported in the original paper, featuring a large sample size, and an elegant multi-session design, were more convincing than what is now additionally reported with the “replication sample”.”*

We thank the Reviewer for the appreciation of the main study. The double-blind replication study was conducted primarily at the request of another Reviewer to rule out experimenter bias in the demonstrated reductions of anxious arousal. Given the limited time and resources at the time, we conducted the replication with the minimal sample size required by our power analyses (power = .83 with n = 9 and Cohen’s d = .96—effect size of the main study; one-tailed t-test). We hope that with the clarification of the significant interaction effect (*p* = .028 one tailed), the Reviewer would consider the replication informative and meaningful. Furthermore, in the revision, we have toned down the replication study by describing it as a replication study “on a smaller scale” (pp. 12 & 31).

p. 12: *To control for potential experimenter biases and to replicate the anxiety reduction effect, we conducted a double-blind, single-session replication study (on a smaller scale), explained in greater detail in the Supplementary Materials.*

p. 31: *To rule out potential experimenter biases, we conducted a double-blind, single-session replication study on a smaller scale.*

1. *Clarifying the relationship (if any) between metrics: alpha power changes & GC posterior frontal alpha connectivity

   Although the authors speculate that the transient changes in alpha power cannot account for the current pattern of behavioral results, it is still possible that those transient changes would propel (give rise to) the tonic-level changes observed in GC posterior frontal alpha connectivity. Thus, the correlations between those metrics should be reported (e.g., in a Supplemental table)—at least for Delta Post-Pre Day 1 (alpha power) Day 4 (baseline) frontal alpha connectivity.*

The Reviewer raised an interesting point. Following the suggestion, we performed supplemental analyses of the relationship between changes in alpha power and GC following stimulation, especially concerning whether one index would predict the other from Day 1 to Day 4. Day 1 alpha power increases did not predict GC changes on Day 4 (*r*’s < .05, *p*’s > .788). In comparison, Day 1 increases in GC marginally predicted Day 4 increases in power (*r* = .31, *p* = .055) although not the baseline shift in power (*r* = .19, *p* = .249), which could be related to the fact that Day 4 baseline power had returned to the Day 1 baseline. Together, these results suggest that alpha connectivity enhancement was unlikely driven by alpha power augmentation, in keeping with the notion that tACS can take effect via cortico-cortical oscillatory reverberation and synaptic plasticity (beyond local neural entrainment). These correlations are now reported in the Supplemental Analyses (pp. 31-32) and Supplemental Table 2 (p. 34).

*pp. 31-32:* *We performed supplemental analyses of the relationship between changes in alpha power and GC following stimulation (collapsed across the two post-stimulation sessions), especially concerning whether one index would predict the other from Day 1 to Day 4. Day 1 alpha power increases did not predict GC changes on Day 4 (r’s < .05, p’s > .788). In comparison, Day 1 increases in GC marginally predicted Day 4 increases in power (r = .31, p = .055) although not the baseline shift (r = .19, p = .249), which could be related to the fact that Day 4 baseline power had returned to the Day 1 baseline. Together, these results suggest that alpha connectivity enhancement was unlikely driven by alpha power augmentation, in keeping with the notion that tACS takes effect via cortico-cortical oscillatory reverberation and synaptic plasticity (Alagapan et al., 2016).*

***Supplemental Table 2.*** *Correlations between Day 1 concurrent and Day 4 changes in alpha power and connectivity*

|  | Day 1 Post-stim rGC | Day 1 Post-stim alpha | Day 4 Baseline rGC | Day 4 Post-stim rGC | Day 4 Baseline power | Day 4 Post-stim power |
| --- | --- | --- | --- | --- | --- | --- |
| Day 1 Post-stim rGC |  | .29 | .51*** | .68*** | .19 | .31^†^ |
| Day 1 Post-stim power |  |  | -.03 | -.05 | .20 | .50*** |

*rGC = right-hemisphere bottom-up alpha Granger causality. Power = alpha power. ***p < .001; † p < .1.*

1. *Clarifying the relationship between metrics: anxious arousal & and ratings, and their association with alpha power changes & GC posterior frontal alpha connectivity:  Somewhat relatedly to the point above (i.e., pertaining to the thorough reporting of dependent variables, which should be in a Supplemental Table, at a minimum), I didn’t easily find in the paper what the relationships are between:*

*3a. The change in Delta (double difference score)* *Day 4 (post – pre) – Day1 (post – pre) for GC and the same (Day 4-Day1, post-pre) double difference score for Anxious arousal and Pleasantness Ratings (especially relevant for negative sounds, which shows a similar pattern to the one seen in Anxious arousal and GC changes). In other words, this contrast would better encapsulate the longitudinal nature of the study. (I realized that the authors answered a related question in response to my prior review, but this should be in the paper/even if in Supplementary material.)*

Following the Reviewer’s suggestion, we have now conducted such correlational analyses. We indeed observed a significant correlation between double-differences in GC and perceived pleasantness of negative sounds (changes in Delta; *r* = .38, *p* = .028). However, we did not see associations in double-differences between GC and anxiety (*p* = .544) or perceived pleasantness of neutral sounds (*p* = .477). No such effects were seen with changes in the increased pleasantness of neutral or negative odors (*p*’s > .103). As reported in the manuscript, the effect of Day 1 GC changes influenced anxiety on Days 2 and 3 but did not on Day 4. The overall lack of correlation in differences between Days 4 and 1 in GC and anxiety (and neutral sound ratings) is consistent with that finding (i.e., long-term effects lasted up to 2 days).

According to the Reviewer’s suggestion, we have summarized these correlations in Supplemental Table 3 and the Supplementary text (p. 32).

p. 32. *As suggested by one of the reviewers, we further examined correlations in double differences [Day 4 (post – pre) – Day1 (post – pre)] between alpha activity and behavior. We observed a significant correlation between double-differences in GC and perceived pleasantness of negative sounds (r = .38, p = .028) but not in double-differences between GC and anxiety (p’s > .54) or perceived pleasantness of neutral sounds (p = .48). No such effects were seen with double-differences in pleasantness of neutral or negative odors (p’s > .10; Supplemental Table 3), either. As reported in the main text, the direct effect of Day 1 GC changes on behavior (i.e., anxiety) was observed on Days 2 & 3 but not on Day 4. The overall lack of correlation between Days 1 and 4 is consistent with the duration (up to 2 days) of long-term effects of tACS.*

*3b. Lastly and likewise, it is possible that I am missing, but the associations between change (delta) in alpha power as well as delta GC with delta pleasantness ratings should be more clearly reported (for Day 1 and Day 4 and possibly their delta, if fruitful—even if only in a Supplementary table, for archival purposes).*

We included a brief summary statement of no significant associations between neural (alpha power or GC) changes and concurrent (but not prospective) behavioral metrics of anxiety/affective ratings (p. 20). As we posited in the Discussion, we tend to think that this direct brain-behavior association would take time to develop as opposed to occur immediately (p. 20: “*the fact that neural effects preceded lasting anxiety changes underscores neural plasticity that develops and consolidates over time, giving rise to enduring amelioration of anxiety*”). Following Reviewer’s suggestion, we have now presented these correlation values in the Sup Tables 4 (auditory stimuli) and 5 (olfactory stimuli) for archival purposes, as reproduced below.

*p. 12: Additional correlational analyses on pleasantness ratings are reported in the Supplementary Material.*

*p. 33:* *In the interest of thoroughness, we evaluated whether changes in alpha power or connectivity were related to changes in perceived pleasantness of sensory stimuli. No such concurrent associations emerged (p’s > .211; Supplemental Tables 4 and 5).*

1. *Page 29 (text): Figure 2A, as currently plotted, does not appear to at all depict a significant Time * Group interaction. (Figure 2B does)—i.e., the lines (even without any confidence interval) between the (groups for “post”) fully overlap. What I am missing? Upon looking at it now for the third time, I see what I was missing: the active group started out lower on alpha power, and increased afterwards (whereas the sham stayed the same). While I appreciate the transparency, and seeing the baseline data, I can imagine that future readers will be equally confused. A note in the figure or text more clearly narrating this pattern of results will aid readability.*

The Reviewer raised an excellent suggestion, and a note is now included in the figure legend and reproduced below.

p. 23: *Note, the Active group demonstrated post-stimulation increases in the alpha band from the baseline while the Sham group demonstrated equivalent alpha power before and after stimulation.*

***Minor Issues:***

1. *Why do the co-authors covary BIS if there were no group differences on that metric, and does not covarying for it change the significance level of the results?*


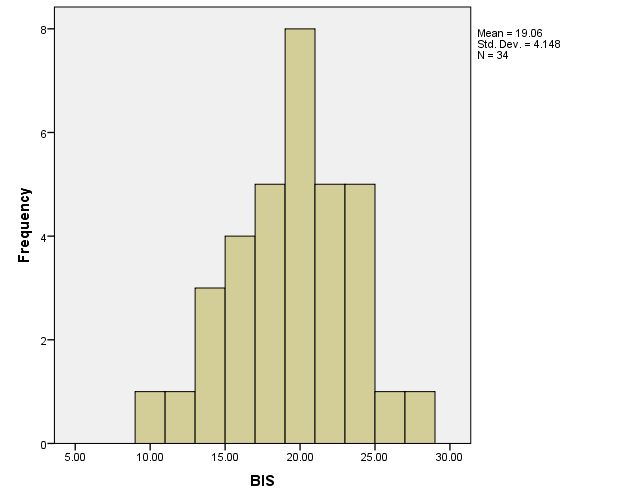
As is well known, BIS reflects trait anxiety, which varies substantially across the population and covaries strongly with state anxiety. Therefore, this variable can influence anxiety ratings and needs to be controlled for *a priori*. As such, we decided *a priori* to include this measure as a covariate in the ANOVA for anxiety ratings (but not for other dependent variables). In our sample, BIS scores ranged widely (10-27 out of a total range of 7-28; see inserted histogram here) and correlated considerably with anxiety ratings (averaged across 4 days; *r* = .22, *p* = .21). The lack of significant group difference in BIS scores here (*t* = -1.25, *p* = .22) is reassuring. However, these BIS differences could still contribute to inter-subject variance in anxiety ratings (which, as the Reviewer has noted below, was substantial), especially considering the repeated measures over the four days, and confound the analyses.

*“does not covarying for it change the significance level of the results?”*

It did only for the ANOVA in the main study, which nonetheless did not affect our interpretation. That is, in the main study, with and without BIS scores, the ANOVA yielded a main effect of tACS at *p* = .039 and *p* = .18, respectively (two-tailed). However, follow-up contrasts (pre-tACS baseline vs. post-tACS anxiety scores) were based on simple t-tests, which were all significant following correction for multiple comparisons (*p*’s < .05 FDR corrected), in support of the effect of tACS in reducing anxious arousal. For the replication study, the inclusion of BIS scores resulted in virtually the same effect of tACS (with and without BIS scores, *p* = .058 and .046, two-tailed, respectively).

In the revised manuscript, we have described the rationale for BIS inclusion more clearly (p. 12), reproduced below.

*p. 12* *Individual differences in trait anxiety are known to be associated with state anxiety and so can influence anxious arousal ratings (especially over repeated measurements across the four days). Therefore, we decided a priori to include scores of the Behavioral Inhibition Scale (BIS, Carver & White, 1994; a measure of trait anxiety) as a covariate in the ANOVA for anxiety ratings (not for other dependent variables).*

1. *What is the presumed (thalamic-independent) source of cortico-cortical alpha oscillations? (Some e.g. Saalman would theorize that the thalamus would coordinate such cortico-cortico interactions)*

The reviewer made a valid point that thalamus plays a key role in synchronizing cortico-cortical alpha interactions. However, direct cortico-cortical alpha interactions (i.e., driven by cortical sources of alpha; Bollimunta et al., 2008) also exist as shown in classic depth recording data (Rockland & Virga, 1989) as well as our previous work (Mo et al., 2011). We have included these details in the revised Discussion (p. 19).

p. 19 *Since such inter-cortical connectivity (especially driven by cortical alpha generators; Bollimunta et al., 2008; Rockland & Virga, 1989) operates outside the relatively encapsulated thalamo-cortical loop…*

1. *Figure 3A: why are the error bars so large for a within-subjects design? Please specify which error bars were used (ideally Lofus and Masson 1994 or a related method: e.g. Cousineau-Morey).*

**
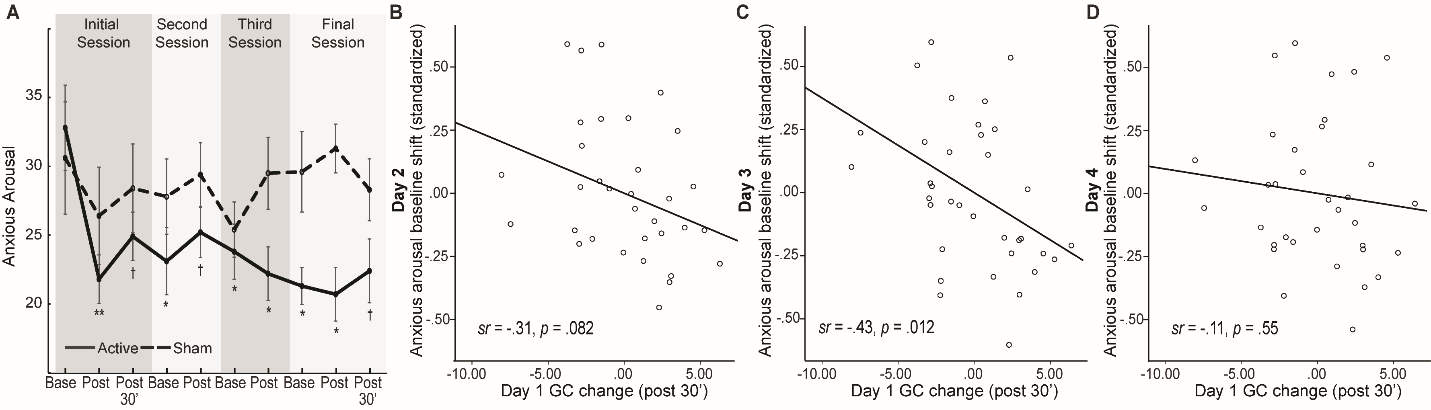
**We suspect the Reviewer was referring to the anxious arousal ratings in Figure 4A, as Figure 3A depicts the raw connectivity spectra without error bars. As mentioned above, indeed, there was substantial inter-subject variability in anxious arousal ratings, which is not unusual for a healthy, unselected sample. Our error bars reflect this raw variance (i.e. S.E.M). Per the Reviewer’s recommendation, we have now adopted the mean adjusted standard error (S.E.E.) as proposed by Lofus and Masson (1994). Figure 4A is reproduced here.

1. *Did the authors ever examine whether alpha power shifts/GC in participants’ actual alpha peak (which they specifically targeted) reflected the presently-reported results? One would assume that those effects would be even more robust than examining the entire alpha range (i.e., since different participants had different alpha values stimulated).*

We had not examined changes at the peak frequency specifically, but rather, applied the *a priori* window of alpha (8-12 Hz) in our analyses (akin to previous tACS studies). However, as the Reviewer suggested, analyses at the peak alpha frequency could be very useful. Surprisingly, we saw little change in the effects: at the peak frequency, effects on alpha power (Time-by-Condition: *F* = 4.18, *p* = .019; *η*_p_*²* = .10) and GC (Session-by-Condition: *F* = 6.96, *p* = .012; *η*_p_*²* = .16) were comparable to the original results for power (*F_1.99, 71.79_* = 3.35, *p = .*041, *η*_p_² = .09) and GC (*F_1, 36_* = 7.71, *p = .*009, *η*_p_² = .18). We surmise that tACS effects very likely spread to neighboring frequencies within the alpha band.

1. *Do the authors have an intuition of why the present results were particularly robust on the right? Are hemispheric interactions significant (i.e., are the main results reported in the study significantly stronger in the right compared to left, or just numerically stronger)?*

The hemispheric interaction was significant, as demonstrated by a significant four-way Hemisphere-by-Session-by-Time-by-Group interaction (p. 13). Right-hemispheric alpha laterality has been observed previously, e.g., greater right-hemisphere alpha modulation of directed attention (Rajagovindan and Ding, 2011, Corbetta and Shulman 2011) and right-hemisphere resting-state connectivity deficits in PTSD (Clancy et al. 2017), which had motivated the inclusion of hemisphere as a factor in the ANOVA. Our intuition for the laterality effect here aligns with the intriguing findings by Medvedev (2014 Neuroimage): at the resting state, the right (vs. left) hemisphere exhibited greater ipsilateral connectivity; also, there was greater right🡪left hemisphere than left🡪right hemisphere connectivity (Granger Causality). While highly tentative, our hunch is that the right (vs. left) hemisphere is especially engaged (at the resting state) in maintaining communication across long-range neural circuits and would thus respond more to tACS-induced circuit perturbation.

1. *There appear to be typos on the Supplemental Material: SD = 0 is reported on P. 62 Lins 26 & 33.*

As confusing as it appears, the SD is indeed 0 as all Active participants reported a “1”, or “None”, for the presence of scalp burning or the attribution of scalp burning to stimulation. We’ve added a note to prevent such confusion (p. 30)

p. 30: *Active* *participants uniformly indicated no scalp burning;*

p. 30:  *Active participants uniformly denied scalp burning as a result of stimulation*

1. *Resonating with a prior major comment, the Supplemental figures 1 & 2 for GC and alpha power are not very helpful to evaluate the results in this replication sample (particularly when devoid of the relevant statistics). If keeping them, it would be easier to evaluate those results by plotting baseline-corrected data with error bars.*

As mentioned above, these figures were included in response to another Reviewer’s request to present “all raw data” of the main experiment, and so we presented them in an unaltered form. Therefore, these figures do not reflect the replication data but rather merely an expansion of the data from the main experiment presented in Figs. 2A and 3A. As mentioned above, we have now included a note in the figure captions to explicate that fact.

1. *I found a few of the phrasings awkward and am noting them below:*

*(Present in multiple locations): The word “deficient” to describe oscillatory power or GC sounds strange to me. Why not use a more descriptive word such as diminished or reduced?*

The word “deficient” was initially used to highlight the alpha pathology (i.e. “oscillopathy”) in patients (e.g. PTSD) in comparison to healthy controls. The Reviewer is absolutely right that “diminished” or “reduced” would be a better choice given the non-clinical sample in the present study. We have thus changed the word to “reduced.”

*Abstract*

*P. 18 L. 13: “contrasts” ? (unclear what this word means in this context)*.

We have now changed it to “differs from.”

*P. 18 L.  20: “advocates”   this feels too strong, the authors probably mean something like “paves the way for”*

Again, we appreciate the Reviewer’s suggestion, which is now implemented.

**Response to Reviewer # 3**

We thank the Reviewer for the appreciation of our revisions. Additionally, the reviewer raises an excellent point regarding the language used to report our findings of changes in pleasantness ratings. Indeed, our new analyses yielded no significant differences between the two modalities. As such, we have modified our language to dampen the suggested comparison of the two modalities, reproduced below (p. 21).

p.21:  *Interestingly, perceived pleasantness of negative odors increased in a transient manner, while lasting increases emerged in auditory stimuli.*
