# Supplementary material for: Lasting connectivity increase and anxiety reduction via transcranial alternating current stimulation
Source: Soc Cogn Affect Neurosci. 2018 Oct 30;13(12):1305–16. doi: 10.1093/scan/nsy096 (PMC6277743; doi:10.1093/scan/nsy096)
Supplement: Supplementary Data [file nsy096_supp.zip › scan-18-051-File008.docx]

**Supplementary Materials**

**SUPPLEMENTARY METHODS**

*Sensory Stimuli*

Intensity levels for presented stimuli are presented below in Supplemental Table 1. All olfactory stimuli were created using 1/3 serial dilutions from a 5% base using mineral oil.

*Behavioral Inhibition Scale (BIS)*

The BIS (Carver and White, 1994) consists of 7 items assessing behavioral avoidance and trait anxiety using a 4 point Likert scale to indicate how much each individual disagrees (1) or agrees (4) with each statement (e.g. “If I think something unpleasant is going to happen I usually get pretty worked up”). This trait anxiety measure was collected at the beginning of the first session and was entered into our statistical analyses as a covariate to control for individual differences in trait anxiety. Note, the groups did not differ on BIS scores (*p* = .22).

*Adverse Effects Questionnaire (AEQ)*

The AEQ (Brunoni *et al.*, 2011) consists of 10 items assessing the experience of common transcranial electrical stimulation symptoms or side effects using a 4 point Likert scale to index the severity of each symptom (1 = absent, 4 = severe) and a 5 point Likert scale to index the likelihood these sensations are related to the stimulation (1 = none, 5 = definite). Across both groups, the most common side-effects experienced were scalp tingling (52.6%) and feelings of sleepiness (47.4%). All participants endorsing scalp tingling attributed it to the stimulation, whereas 83.5% of those endorsing sleepiness thought it was related to the stimulation. Group differences in the experience of tACS-induced symptoms or side effects emerged only in the presence of scalp burning and the ascribed likelihood that sensations of scalp pain and burning could be attributed to the tACS administration. Importantly, compared to the Active group (*M* = 1.00, *SD* = 0.00—Active participants uniformly indicated no scalp burning), the Sham group (*M* = 1.35, *SD* = .77) indicated increased severity of scalp burning, *t*_34_ = -3.13, *p* = .004. Additionally, the Sham group attributed the sensations of scalp pain (*M* = 2.53, *SD* = 1.66) and burning (*M* = 2.00, *SD* = 1.54) to the perceived stimulation more so than the Active group (pain: *M* = 1.47, *SD* = 1.17; burning: *M* = 1.00, *SD* = 0.00—Active participants uniformly denied scalp burning as a result of stimulation), *t*’s < -.220, *p*’s < .033. The groups did not differ in ratings of any other symptoms (*p*’s > .114). Therefore, these results corroborated subject report during debriefing, further confirming that the sham group was not aware of the sham stimulation.

**SUPPLEMENTARY RESULTS**

**Double-blind Replication Study**

To rule out potential experimenter biases, we conducted a double-blind, single-session replication study on a smaller scale. Additionally, we applied random noise stimulation (tRNS; 1-200 Hz) as an active control in this study to remove random electrical stimulation effects. Eighteen participants (n = 9 per group) were recruited for the replication, based on power analyses (power = .81 with n =9 and Cohen’s *d* = .92; one-tailed t-test). Participants completed the same visual analog scale (VAS) anxiety rating immediately before and after 30 minutes of tACS or tRNS. The two groups did not differ in baseline, pre-stimulation anxious arousal (*t* = 1.22, *p* = .239). Given the hypothesized direction of effects (i.e. reductions in anxious arousal), we applied one-tailed tests. Similar to the initial study, BIS scores were included as a covariate to control for individual differences in trait-level anxiety.

An rANOVA of Time (Pre vs. Post) and Group (tACS/Active, tRNS/Control) on anxious arousal revealed a Time x Group interaction *(F_1, 15_* = 4.29*, p = .*028 one tailed*, ηp²* = .22), consistent with the effect size found in the main experiment. Follow-up contrasts within each group revealed a reduction in anxious arousal in the tACS group (*t* = 1.96, *p* = .043 one-tailed), but not the tRNS group (*t* = -1.01, *p* = .172 one-tailed). Therefore, these findings replicated our initial study and ruled out possible confounds of experimenter biases and random stimulation.

**Associations between changes in alpha power and posterior🡪frontal connectivity**

We performed supplemental analyses of the relationship between changes in alpha power and GC following stimulation (collapsed across the two post-stimulation sessions), especially concerning whether one index would predict the other from Day 1 to Day 4. Day 1 alpha power increases did not predict GC changes on Day 4 (r’s < .05, p’s > .788). In comparison, Day 1 increases in GC marginally predicted Day 4 increases in power (r = .31, p = .055) although not the baseline shift (r = .19, p = .249), which could be related to the fact that Day 4 baseline power had returned to the Day 1 baseline. Together, these results suggest that alpha connectivity enhancement was unlikely driven by alpha power augmentation, in keeping with the notion that tACS takes effect via cortico-cortical oscillatory reverberation and synaptic plasticity (Alagapan et al., 2016).

**Changes in connectivity increases were associated with greater increases in perceived pleasantness of negative sounds between sessions**

As suggested by one of the reviewers, we further examined correlations in double differences [Day 4 (post – pre) – Day1 (post – pre)] between alpha activity and behavior. We observed a significant correlation between double-differences in GC and perceived pleasantness of negative sounds (r = .38, p = .028) but not in double-differences between GC and anxiety (p’s > .54) or perceived pleasantness of neutral sounds (p = .48). No such effects were seen with double-differences in pleasantness of neutral or negative odors (p’s > .10; Supplemental Table 3), either. As reported in the main text, the direct effect of Day 1 GC changes on behavior (i.e., anxiety) was observed on Days 2 & 3 but not on Day 4. The overall lack of correlation between Days 1 and 4 is consistent with the duration (up to 2 days) of long-term effects of tACS.

**Increases in connectivity were not related to increases in perceived pleasantness of auditory stimuli, neither prospectively nor concurrently**

In the interest of thoroughness, we evaluated whether changes in alpha power or connectivity were related to changes in perceived pleasantness of sensory stimuli. No such concurrent associations emerged (p’s > .211; Supplemental Tables 4 and 5).

**SUPPLEMENTAL TABLES**

| **Supplemental Table 1.** Intensities of presented sensory stimuli at weak, medium, and strong levels. | | | |
| --- | --- | --- | --- |
|  | Weak | Medium | Strong |
| Sound – neutral | 47 dB | 50 dB | 55 dB |
| Sound – scream | 47 dB | 54 dB | 67 dB |
| Sound – vomit | 50 dB | 58 dB | 67 dB |
| Odor – neutral | .54% | 1.65% | 5% |
| Odor – negative | .54% | 1.65% | 5% |
| Auditory stimuli intensities are presented in decibel readings from headphones, and olfactory stimuli are presented in concentrations of pure base odorant. | | | |

| **Supplemental Table 2.** Correlations between Day 1 concurrent and Day 4 changes in alpha power and connectivity. | | | | | | |
| --- | --- | --- | --- | --- | --- | --- |
|  | Day 1 Post-stim rGC | Day 1 Post-stim alpha | Day 4 Baseline rGC | Day 4 Post-stim rGC | Day 4 Baseline power | Day 4 Post-stim power |
| Day 1 Post-stim rGC |  | .29 | .51*** | .68*** | .19 | .31^†^ |
| Day 1 Post-stim power |  |  | -.03 | -.05 | .20 | .50*** |
| rGC = right-hemisphere bottom-up alpha Granger causality. Power = alpha power. ****p* < .001; † *p* < .1 | | | | | |  |

| **Supplemental Table 3.** Correlations between differences in alpha power and connectivity between Day 4 and Day 1. | | | | | |
| --- | --- | --- | --- | --- | --- |
|  | Anxiety Delta change | Neutral sound Delta change | Negative sound Delta change | Neutral odor Delta change | Negative odor Delta change |
| Power Delta change | -.19 | -.04 | .17 | -.27 | -.29 |
| rGC Delta change | -.11 | .13 | .38* | -.04 | -.05 |
| rGC = right-hemisphere bottom-up alpha Granger causality. Power = alpha power. * *p* < .05. | | | | | |

| **Supplemental Table 4.** Correlations between changes in alpha power and connectivity and changes in pleasantness ratings of auditory stimuli. | | | | | | |
| --- | --- | --- | --- | --- | --- | --- |
|  | Sess. 1 Post-stim Neutral Sound | Sess. 1 Post-stim Negative Sound | Sess. 4 Baseline Neutral Sound | Sess. 4 Baseline Negative sound | Sess. 4 Post-stim Neutral Sound | Sess. 4 Post-stim Negative Sound |
| Sess. 1 Post-stim power | .17 | .24 | -.15 | -.17 | .28 | .19 |
| Sess. 1 Post-stim rGC | .03 | .12 | .17 | -.03 | .18 | .06 |
| Sess. 4 Baseline power | .09 | -.01 | -.06 | -.03 | -.09 | -.01 |
| Sess. 4 Baseline rGC | .07 | .24 | .03 | .17 | .05 | .15 |
| Sess. 4 Post-stim power | .14 | .04 | .13 | -.20 | .11 | .05 |
| Sess. 4 Post-stim rGC | -.02 | -.04 | .08 | -.03 | .17 | -.03 |
| rGC = right-hemisphere bottom-up alpha Granger causality. Power = alpha power. | | | | | | |

| **Supplemental Table 5.** Correlations between changes in alpha power and connectivity and changes in pleasantness ratings of olfactory stimuli. | | | | | | |
| --- | --- | --- | --- | --- | --- | --- |
|  | Sess. 1 Post-stim Neutral odor | Sess. 1 Post-stim Negative odor | Sess. 4 Baseline Neutral odor | Sess. 4 Baseline Negative odor | Sess. 4 Post-stim Neutral odor | Sess. 4 Post-stim Negative odor |
| Sess. 1 Post-stim power | -.15 | -.19 | -.15 | -.15 | -.09 | -.05 |
| Sess. 1 Post-stim rGC | -.22 | .06 | -.21 | -.04 | -.19 | -.01 |
| Sess. 4 Baseline power | -.07 | .17 | -.06 | -.02 | -.09 | -.05 |
| Sess. 4 Baseline rGC | -.24 | .19 | -.17 | .08 | -.21 | .02 |
| Sess. 4 Post-stim power | -.09 | -.01 | -.06 | -.10 | -.18 | -.16 |
| Sess. 4 Post-stim rGC | -.15 | .09 | -.21 | -.13 | -.23 | -.16 |
| rGC = right-hemisphere bottom-up alpha Granger causality. Power = alpha power. | | | | | | |

**SUPPLEMENTAL FIGURES**


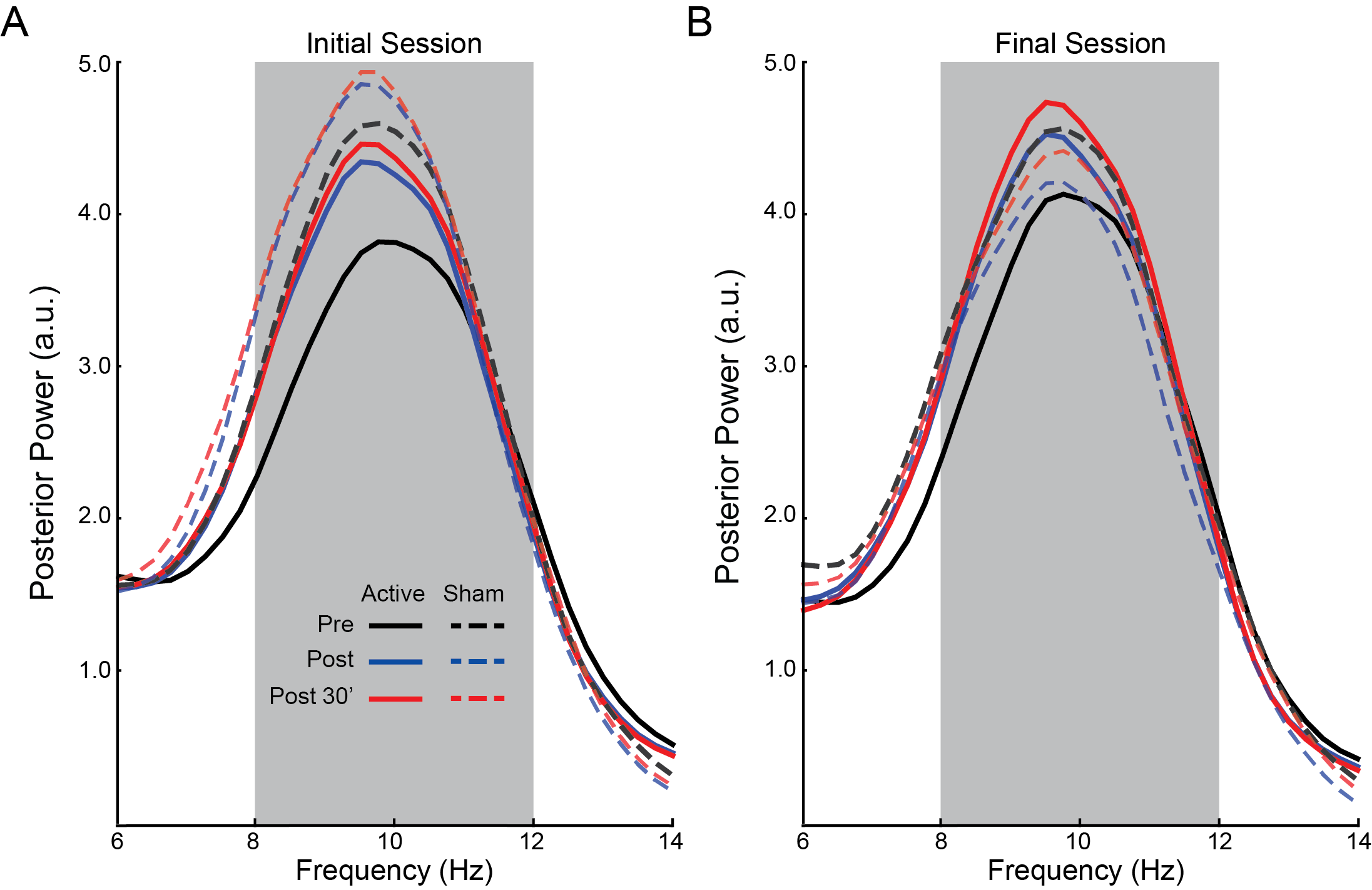


**Figure S1.** Raw power spectra from occipitoparietal electrodes for all time points (pre-, post-, post-30 minutes stimulation) for the A) initial and B) final sessions from the main experiment. This figure represents an expansion of Figure 2A in the main text, which was collapsed across sessions to demonstrate the significant Time-by-Condition interaction.


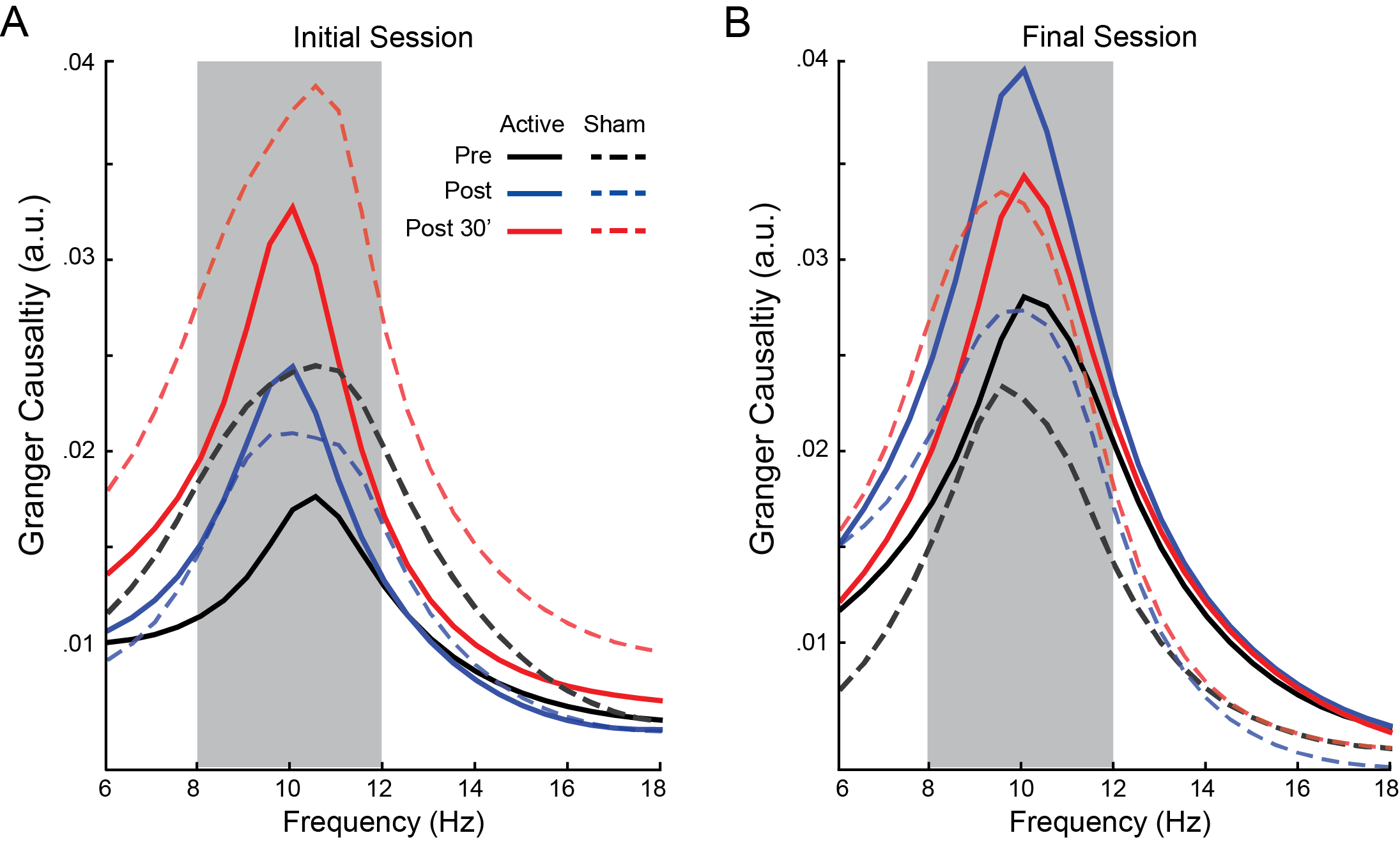


**Figure S2.** Raw spectra of right-hemisphere posterior🡪frontal Granger causality for each group for all time points (pre-, post-, post-30 minutes stimulation) for the A) initial and B) final sessions from the main experiment. This figure represents an expansion of Figure 3A in the main text, which was collapsed across time to demonstrate the significant Session-by-Condition interaction.

**SUPPLEMENTARY REFERENCES**

Brunoni, A. R., Amadera, J., Berbel, B., Volz, M. S., Rizzerio, B. G. & Fregni, F. (2011) 'A systematic review on reporting and assessment of adverse effects associated with transcranial direct current stimulation', *Int J Neuropsychopharmacol*, **14**(8), pp. 1133-1145.

Carver, C. S. & White, T. L. (1994) 'Behavioral-Inhibition, Behavioral Activation, and Affective Responses to Impending Reward and Punishment - the Bis Bas Scales', *Journal of Personality and Social Psychology*, **67**(2), pp. 319-333.
