# Supplementary material for: Lasting connectivity increase and anxiety reduction via transcranial alternating current stimulation
Source: Soc Cogn Affect Neurosci. 2018 Oct 30;13(12):1305–16. doi: 10.1093/scan/nsy096 (PMC6277743; doi:10.1093/scan/nsy096)
Supplement: Supplementary Data [file nsy096_supp.zip › nsy096.pdf]

Lasting connectivity increase and anxiety reduction via transcranial alternating current stimulation

Abbreviated title: **Lasting effects of α-tACS**

Kevin J. Clancy, M.S.[1], Sarah K. Baisley, Ph.D.[1], Alejandro Albizu, B.S.[1], Nika Kartvelishvili, B.A.[1], Mingzhou Ding, Ph.D.[2], & Wen Li, Ph.D.[1]

[1] Department of Psychology, Florida State University, Tallahassee, FL

[2]J Crayton Pruitt Family Department of Biomedical Engineering, University of Florida, Gainesville, FL

Correspondence concerning this article should be addressed to Wen Li (wenli@psy.fsu.edu) and Kevin Clancy (clancy@psy.fsu.edu), Department of Psychology, Florida State University, B342 PDB, 1107 W. Call St., Tallahassee, FL 32303.

Acknowledgement: This research was supported by the National Institute of Mental Health grants R01MH093413 (W.L.) and R21MH112206 (M.D.) and the FSU Chemical Senses Training (CTP) Grant Award T32DC000044 (K.C.) from the National Institutes of Health (NIH/NIDCD).

Competing financial interests: The authors declare no competing financial interests or potential conflicts of interest.

Word Count – Abstract: 200

Word Count – Text (excl. references, tables, figure legends, abstract): 5000

Title Character Count – 100

ABSTRACT

Growing evidence of transcranial alternating current stimulation (tACS) modulating intrinsic neural oscillations has spawned interest in applying tACS to treat psychiatric disorders associated with aberrant neural oscillations. The alpha rhythmic activity is known to dominate neural oscillations at the awake restful state, while attenuated resting-state alpha activity has been implicated in anxious mood. Administering repeated alpha-frequency tACS (at individual peak alpha frequency; 8-12 Hz) over 4 consecutive days (in the experiment group, sham stimulation in the control group), we demonstrated immediate and lasting (> 24 hours) increases in resting-state posterior→frontal connectivity in the alpha frequency, quantified by Granger causality. Critically, this connectivity enhancement was accompanied by sustained reductions in both anxious arousal and negative perception of sensory stimuli. Resting-state alpha power also increased, albeit only transiently, reversing to the baseline level within 24 hours after tACS. Therefore, the lasting enhancement of long-range alpha connectivity due to α-tACS differs from local alpha activity that is nonetheless conserved, highlighting the adaptability of alpha oscillatory networks. In light of increasing recognition of large-scale network dysfunctions as a transdiagnostic pathophysiology of psychiatric disorders this enduring connectivity plasticity, along with the behavioral improvements, paves the way for tACS applications in clinical interventions of psychiatric "oscillopathies."

Key Words: Alpha oscillations, Resting-state, Granger causality, neuromodulation, sensory affect.

INTRODUCTION

Brain oscillations play important roles in various mental activities (Başar *et al.*, 2001; Buzsaki *et al.*, 2013). Aberrations in these oscillations have been observed in a host of neuropsychiatric disorders, potentially underpinning their pathophysiology (i.e., "oscillopathies") (Basar, 2013; Buzsaki *et al.*, 2013). Recent advances in transcranial alternating current stimulation (tACS) demonstrate that by applying weak electric fields tuned to the rhythms of endogenous oscillations through the scalp, tACS can directly interact with and thereby modify brain oscillations (Frohlich and McCormick, 2010; Thut *et al.*, 2011; Antal and Paulus, 2013; Herrmann *et al.*, 2013). This non-invasive neuromodulation technology thus holds great promise for therapeutic interventions of oscillopathies (Frohlich, 2015; Abend *et al.*, 2016). However, clinical efficacy of tACS hinges on whether its neuromodulatory effects would persist and induce lasting behavioral changes.

Alpha oscillations (8-12 Hz; posteriorly distributed) are the most prominent rhythmic activity in the awake restful human brain and are thought to be generated in the thalamo-cortical network (Hughes and Crunelli, 2005; Bollimunta *et al.*, 2008; Buzsaki *et al.*, 2013). Alpha oscillations exert largely inhibitory modulation on multiple cognitive processes, including attention and sensory perception (Palva and Palva, 2007; Bollimunta et al., 2008; Foxe and Snyder, 2011; Klimesch, 2012). For example, heightened pre-stimulus alpha power is associated with reduced perceptual sensitivity to the stimuli (Mathewson et al., 2009; Dugue et al., 2011), and attenuated alpha power is coupled with enhanced visual attention (Rajagovindan and Ding, 2011). Furthermore, via long-range, posterior→frontal projections, alpha oscillations mediate inhibitory bottom-up information flow from the sensory cortex to frontal regions to influence global neural activity (Hillebrand et al., 2016; Sadaghiani et al., 2016; Johnson et al., 2017; Tang

et al., 2017a). For instance, weak posterior→frontal alpha connectivity is associated with strong frontal gamma activity and impaired cognitive control (Clancy et al., 2017; Johnson et al., 2017).

Alpha oscillations are particularly important for inter-regional communication across large-scale resting-state networks (RSNs), especially the default mode network (DMN) and the salience network (SN) (Laufs et al., 2003; Mantini et al., 2007; Sadaghiani et al., 2010; Mo et al., 2013; Tang et al., 2017a), which are strongly implicated in emotion- and arousal-related processes (Raichle et al., 2001; Sridharan et al., 2008) and affective disorders (Sripada et al., 2012b; Peterson et al., 2014; Alvarez et al., 2015). Furthermore, akin to the reciprocal roles these two RSNs play in regulating arousal (Menon and Uddin, 2010; Young et al., 2017), alpha activity is positively correlated with DMN activity and negatively with SN activity (Sadaghiani et al., 2010; Ros et al., 2013), serving a key function in maintaining a state of restful alertness (Gath et al., 1983; Klimesch, 1999). Relatedly, neurofeedback training targeting alpha activity has been shown to modulate DMN and SN activity (Ros et al., 2013) and reduce anxiety (Hammond, 2005), while meditation training that leads to reduced anxious mood has been linked to augmentation of resting-state alpha activity and reorganization of these RSNs (Cahn and Polich, 2006; Fan et al., 2014; Tang et al., 2017b).

Consistent with growing evidence of DMN and SN dysfunctions in posttraumatic stress disorder (PTSD) (Sripada et al., 2012a), recent data from our lab revealed deficits in resting-state alpha power and posterior→frontal alpha connectivity in patients with PTSD (Clancy et al., 2017). Importantly, reduced alpha connectivity correlated with greater hypervigilance, a key hyperarousal symptom of PTSD. These findings thus implicate reduced alpha activity that translates to behavioral expressions of hypervigilance and anxious arousal, thereby presenting a concrete and spatiotemporally specific target for clinical intervention via alpha-frequency tACS

(α-tACS). That is, provided alpha enhancement via α-tACS could persist, tACS could achieve stable anxiety reduction and clinically meaningful symptom alleviation.

Empirical and computational models suggest that while tACS induces short-term effects via entrainment of local neuronal firing synchrony, long-term plastic effects would arise from strengthened oscillatory circuits via spike-timing dependent plasticity (STDP) and long-term potentiation (LTP) at the synapse (Thut *et al.*, 2011; Herrmann *et al.*, 2013; Reato *et al.*, 2013). "Plasticity-like" effects of tACS (primarily, local power enhancement outlasting the period of stimulation) have been consistently demonstrated (Neuling *et al.*, 2013; Vossen *et al.*, 2015; Kasten *et al.*, 2016), but evidence for long-term effects remains limited. A recent α-tACS study tracked alpha power change over 90 minutes post-stimulation, but in contrast to enduring LTP that typically lasts for hours to days (Abraham, 2003), alpha power enhancement persisted only for 70 minutes post-stimulation (Kasten *et al.*, 2016).

The acute but transient tACS effects could reflect the labile, early phase of LTP that is susceptible to other synaptic activities such as endogenous, corrective inputs from other neurons and changes in the extracellular environment (Abraham, 2003; Huang et al., 2017). Targeting primarily the cortex (Frohlich and McCormick, 2010; Thut *et al.*, 2011; Herrmann *et al.*, 2013), exogenous oscillatory inputs of most tACS applications rarely reach deep subcortical generators (Neuling *et al.*, 2012). Since the thalamo-cortical circuit operates as a unified generator of alpha oscillations (Hughes and Crunelli, 2005; Bollimunta *et al.*, 2008; Buzsaki *et al.*, 2013), deep thalamic drives can thwart early-phase LTP at the synapses, resetting cortical alpha oscillations after α-tACS. That said, as repeated stimulation can facilitate the induction of late-phase LTP and long-term neural plasticity (Fregni *et al.*, 2006; Reis *et al.*, 2009; Huang *et al.*, 2017), lasting

tACS effects may emerge with multi-session (vs. single-session reported thus far) tACS protocols.

Besides local cortical power increases, tACS, via the purported synaptic strengthening, is likely to induce inter-cortical connectivity plasticity. Indeed, tACS can intensify rhythmic reverberations across the abundant recurrent inter-cortical connections in the brain, thereby modifying large-scale network dynamics (Clopath *et al.*, 2010; Alagapan *et al.*, 2016; Moisa *et al.*, 2016). This account would hold especially true for α-tACS given the instrumental role of alpha oscillations in long-range interactions (Palva and Palva, 2007; Cabral-Calderin *et al.*, 2016). Importantly, while local cortical alpha oscillations can be restored by thalamic inputs, plasticity in inter-cortical connectivity, presumably relatively independent of thalamic drives, may persist over time and mediate lasting anxiety reduction.

Therefore, administering repeated sessions of high-density α-tACS targeting the occipitoparietal cortex implicated in PTSD-related alpha deficiency (Clancy *et al.*, 2017) (Figure 1), we set out to test two hypotheses. First, α-tACS could generate lasting enhancement of alpha activity, including alpha power and long-range connectivity, particularly posterior→frontal projections that dominate resting-state alpha connectivity (Engel *et al.*, 2001; Tang *et al.*, 2007; Sadaghiani *et al.*, 2012; Hillebrand *et al.*, 2016; Wang *et al.*, 2016). Second, these neural changes would be accompanied by lasting anxiety reduction, measured here by anxious arousal and negative perception of sensory stimuli (in the auditory and olfactory modalities given their sensitivity to states of anxious arousal) (Adler *et al.*, 1988; Grillon *et al.*, 1991; Krusemark *et al.*, 2013). Assessments were administered immediately and 30 minutes after α-tACS on the first and last (fourth) day, except for anxious arousal that was rated daily to track sustained and accumulating effects of α-tACS on this key outcome.

METHODS

Participants

38 healthy volunteers (18 female, 19.7 ± 2.0 years of age) participated in this study after providing written, informed consent approved by the Florida State University Institutional Review Board. No participants reported a history of neurological or psychiatric disorders and all were deemed tACS compatible (e.g. no metal implants, neurological surgery, or pregnancy). Participants were randomly assigned to two groups, an Active ($N = 21$) and a Sham control group ($N = 17$). Four participants in the Active group did not provide behavioral ratings and were thus included in the EEG analyses only. Groups did not differ in age or gender distribution (p's > .14). Participants were blind to their group assignment (see tACS procedures below).

Ratings and questionnaires

Pleasantness ratings

Auditory and olfactory stimuli were rated for perceived pleasantness on a visual analog scale presented on a computer monitor (0-100 from most unpleasant to most pleasant; 50 = neutral). Auditory stimuli included a neutral (flat tone) and two negative (screaming and vomiting) sounds, delivered through headphones for one second each. Olfactory stimuli included a neutral (acetophenone; Fisher Scientific, NH) and negative (burning rubber; ScentAir™, NC) odor, delivered through a bottle. All stimuli were presented at three intensities: weak, medium, and strong.

Subjective units of anxious arousal

Participants rated their current state of anxious arousal using a visual analog scale from 0 (not at all) to 100 (extremely), in accordance with standard ratings of Subjective Units of Distress (Wolpe and Lazarus, 1966).

tACS

tACS was administered using a Soterix Medical 4x1 high-density (HD) transcranial electrical stimulation system (New York, NY, USA). Stimulation electrodes were placed in a 4x1 montage over occipitoparietal sites, with the central electrode receiving electrical current from the four surrounding electrodes (Figure 1A). Stimulation sites were selected to maximally target the occipitoparietal area (cuneus/precuneus) where reduced alpha oscillations in patients with PTSD have been observed (Clancy *et al.*, 2017). Current flow modeling (Soterix Medical HDExplore Software, New York, USA) indicated maximal electric field intensity (0.21 V/m; with a +/- 2 mA stimulation current) in the right dorsal occipital cortex (peak voxel: 29, -91, 18; Montreal Neurological Institute coordinates; Figure 1A). Electric field intensity did not exceed 0.02 V/m in frontal regions.

Stimulation was administered with eyes open for 30 minutes using a +/- 2 mA sinusoidal current oscillating at individual participants' occipitoparietal peak alpha frequency (PAF). The PAF was identified as the peak frequency within the alpha-range (8-12 Hz) with a 0.5 Hz frequency resolution across occipitoparietal electrodes during the initial (session 1) baseline EEG recordings. Individual PAFs ranged from 8 to 11.5 Hz (M = 9.93, SD = 0.66 Hz) in the sample, equivalent between the two groups (p = .853). All participants were familiarized with tACS-induced skin sensations with a brief, 30 second pulse of random noise stimulation. During stimulation, the current ramped up to 2 mA over a span of 10 seconds. Stimulation was then discontinued in Sham participants, and reintroduced during the last 10 seconds to mitigate

awareness of experimental condition. All participants were told that they would receive stimulation, and their lack of awareness of sham stimulation was confirmed during the debriefing at the end of the study and by their responses to the Adverse Effects Questionnaire (Supplementary Material).

Experimental Design

The experiment consisted of four sessions, each separated by 24 hours (Figure 1B). During the first and last sessions, participants started with a subjective rating of anxious arousal, followed by a two-minute resting, eyes-open EEG recording and then pleasantness ratings. Participants then received 30 minutes of α-tACS, and then repeated a set of arousal ratings, resting EEG recordings, and pleasantness ratings. After a 30-minute break, participants again repeated a set of arousal ratings, resting EEG recordings, and pleasantness ratings. The second and third sessions included α-tACS, and pre-/post-stimulation ratings of anxious arousal.

EEG acquisition and analyses

EEG data were recorded from a 32-channel BrainProducts actiChamp system (1000 Hz sampling rate, 0.05 – 200 Hz online bandpass filter, referenced to the FCz channel). Electro-oculogram (EOG) was recorded using four electrodes with vertical and horizontal bipolar derivations. EEG/EOG data were downsampled to 250 Hz, high-pass (1 Hz) and notch (60 Hz) filtered, and re-referenced to the average of all EEG channels. We applied the *Fully Automated Statistical Thresholding for EEG artifact Rejection* algorithm for artifact detection and rejection (Nolan *et al.*, 2010).

Power analyses

EEG oscillation power was computed for individual channels for each epoch (1-sec) using the multitaper spectral estimation technique (Mitra and Pesaran, 1999). Alpha (8-12 Hz) power was normalized by the mean power for the global spectrum (1-50 Hz) within each epoch. Alpha power was extracted from occipitoparietal (right, middle, and left) electrodes, where they were maximally distributed (Palva and Palva, 2007; Foxe and Snyder, 2011; Klimesch, 2012).

Directed alpha-frequency connectivity (Granger causality) analyses

Alpha-frequency Granger causality (GC) analysis (Geweke, 1982; Ding *et al.*, 2006) was performed to assess posterior→frontal causal connectivity in the alpha band. Following transformation into reference-free, current source density data using the surface Laplacian algorithm (Perrin *et al.*, 1989; Nunez *et al.*, 1997; Wang *et al.*, 2016), EEG data from ipsilateral frontal-posterior pairs were submitted to bivariate autoregressive (AR) modeling, from which Granger causality spectra were derived (Ding *et al.*, 2000; Ding *et al.*, 2006). A model order of 20 (80 ms in time for a sampling rate of 250 Hz) was chosen in a two-step process: (1) Akaike Information Criterion (AIC) and (2) comparing spectral estimates obtained by the AR model and that by the Fourier-based method for data pooled across all subjects (Wang *et al.*, 2016).

Statistical analyses

Three key effects were directly relevant to our hypotheses: (1) immediate and short-term (30 minutes) effects based on within-session changes (effect of Time); (2) long-term effects based on differences between the initial and final sessions (i.e., effects lasting over 24 hours from the preceding stimulation; effect of Session); and (3) improved within-session tACS

effectiveness, i.e., whether within-session effects increased from the initial to the final session (interaction of Time-by-Session). As such, we examined interactions of Group with Time and/or Session, in that these time/session effects differed between Active and Sham groups. Wherever appropriate, we started the analysis with an omnibus ANOVA and followed significant effects with post hoc tests, which were further controlled for Type I error using the false discovery rate (FDR) criterion.

Occipitoparietal alpha power and feedforward alpha GC at each time point (*t*) were decibel-normalized relative to the initial (*i*), first-session pre-tACS, baseline values [$power_{db} = 10*log10(power_t/power_i)$; $GC_{db} = 10*log10(GC_t/GC_i)$] to control for variations in baseline activity (Cohen, 2014; Vossen *et al.*, 2015; Kasten *et al.*, 2016). Note, the groups did not differ in initial baseline alpha power or GC (p's > .19). Decibel-normalized values were submitted to omnibus rANOVAs of Site (power: left, middle, right; GC: left, right hemisphere), Session (initial, final), Time (pre, post, post-30 minutes), and Group (active, sham). Site was included to consider previously reported effects of right-hemisphere laterality of alpha power on directed attention (Corbetta and Shulman, 2011; Rajagovindan and Ding, 2011) and connectivity deficits in PTSD (Clancy et al., 2017).

Pleasantness and anxious arousal ratings at each time point (*t*) were normalized using the difference-over-sum method to the initial (*i*) baseline [$(rating_t – rating_i)/(rating_t + rating_i)$] to account for variations in baseline ratings (Cohen and Maunsell, 2010). The groups did not differ in initial baseline ratings (p's > .12). Pleasantness ratings were submitted to omnibus rANOVAs of Valence (neutral, threat), Intensity (strong, medium, weak), Session (initial, final), Time (pre, post, post-30 minutes), and Group. Ratings of anxious arousal were submitted to an omnibus rANOVA of Session (initial, second, third, final), Time (pre, post) and Group. As post-30-

minute anxious arousal ratings were acquired only in the first and last sessions and there was no difference between immediate and 30-minute ratings in either session (p's > .36), we averaged the immediate and 30-minute ratings to reflect post-stimulation ratings for each session. Individual differences in trait anxiety are known to be associated with state anxiety and so can influence anxious arousal ratings (especially over repeated measurements across the four days). Therefore, we decided *a priori* to include scores of the Behavioral Inhibition Scale (BIS, Carver & White, 1994; a measure of trait anxiety) as a covariate in the ANOVA for anxiety ratings (not for other dependent variables).

Finally, a prospective examination of anxiety reduction on subsequent days (Days 2-4) as predicted by Day 1 alpha enhancement was conducted. This analysis was restricted to Day 1 alpha changes as the predictor because EEG was not acquired on Days 2 & 3 in order to ease the burden on participants. As the key outcome measure of the study, anxious arousal ratings were collected each day, which allowed for examination of long-term effects of Day 1 alpha and/or GC enhancement on subsequent anxiety levels. Specifically, we entered baseline shifts in anxious arousal on Days 2-4 (from Day 1) into an rANCOVA with Session (Sessions 2, 3, and 4) as an independent variable and Day 1 alpha activity change (power/GC) as a covariate. Additional correlational analyses on pleasantness ratings are reported in the Supplementary Materials.

To control for potential experimenter biases and to replicate the anxiety reduction effect, we conducted a double-blind, single-session replication study (on a smaller scale), explained in greater detail in the Supplementary Materials.

RESULTS

tACS induced immediate and short-term increases in occipitoparietal alpha power

An omnibus repeated-measures Analysis of Variance (rANOVA) of Site (left, middle, right), Session (initial, final), Time (pre, post, post-30 minutes), and Group (active, sham) on posterior alpha power revealed a significant interaction of Time and Group ($F_{1.99,\ 71.79} = 3.35$, $p = .041$, $\eta_p^2 = .09$), such that, across occipitoparietal sites and the two sessions, an effect of Time arose in the Active group ($F_{1.55,\ 30.98} = 7.99$, $p = .003$, $\eta_p^2 = .29$) but not the Sham group ($p = .485$; Figure 2A). Follow-up contrasts within the Active group demonstrated power (collapsed across three posterior sites) increases both immediately ($p = .0003$) and 30 minutes ($p = .006$) after stimulation. A complementary bin-by-bin (.25 Hz each) examination across the global frequency spectrum (1-50 Hz) revealed that this significant Time-by-Group interaction appeared selectively in the targeted alpha band (8-10.5 Hz; Figure 2A yellow line), confirming the specific modulation of alpha power. However, baselines for the two sessions did not differ in either group (p's > .598), failing to indicate a long-term shift in alpha power (Figure 2B). Finally, there was no Session-by-Time-by-Group interaction ($p = .268$) such that the within-session effects did not change from the first to the last session. Overall, the results confirmed that α-tACS produced an immediate and specific increase in alpha power, lasting 30 minutes post-stimulation.

tACS induced immediate, short-term, and long-term increases in posterior→frontal alpha connectivity

A similar omnibus rANOVA of Site (right, left hemisphere), Session, Time, and Group on posterior→frontal alpha GC showed a significant four-way interaction of Site-by-Session-by-Time-by-Group ($F_{1.98,\ 71.13} = 4.18$, $p = .020$, $\eta_p^2 = .10$). A follow-up ANOVA (Session-by-Time-by-Group) in the right hemisphere revealed a Session-by-Group interaction ($F_{1,\ 36} = 7.71$, $p = .009$, $\eta_p^2 = .18$; Figure 3A), which was substantiated by a significant increase from the initial to

the final session in the Active ($F_{1, 20}$ = 12.11, p = .002, η_p^2 = .38) but not Sham (p = .487) group. Furthermore, as illustrated in Figure 3B, relative to the initial baseline, GC at all subsequent assessments was augmented in the Active group (t's < -2.28, p's < .05 FDR corrected) but not in the Sham group (p's > .190), reflecting immediate and sustained enhancement of connectivity via tACS. A bin-by-bin (.25 Hz each) survey across the global frequency spectrum showed that this Session-by-Group interaction spanned almost the entire alpha band (8.25-12 Hz) but no other frequency band, supporting specific modulation of alpha-frequency GC. Importantly, the Active group (but not the Sham group, p = .225) demonstrated a significant baseline shift from the initial to the final session ($F_{1, 20}$ = 18.46, p = .0004, η_p^2 = .48), highlighting a long-term effect of tACS. Finally, there was no Session-by-Time-by-Group interaction (p = .147) such that the within-session effects did not change with repeated stimulation.

A similar follow-up ANOVA in the left hemisphere showed an interaction of Session-by-Time-by-Group ($F_{1.99, 71.58}$ = 2.84, p = .065, η_p^2 = .07). However, this effect did not lend support to the notion of increased tACS potency over repeated stimulation. The three-way interaction was substantiated by an interaction of Session-by-Time in the Active ($F_{1.99, 39.81}$ = 3.13, p = .055, η_p^2 = .14) but not Sham (p = .363) group. Specifically, the Active group exhibited an effect of Time in the initial session ($F_{1.96, 39.28}$ = 3.87, p = .030, η_p^2 = .16) with a trend of immediate alpha GC increase (p = .065) and no change 30 minutes later (p = .504), in contrast to no effect of Time in the final session (p = .730). Finally, there was no baseline shift in the Active (vs. Sham) group (p = .367), suggesting no lasting effect of stimulation in the left hemisphere.

tACS reduced anxious arousal

An omnibus rANOVA of Session, Time, and Group on anxious arousal ratings revealed a main effect of Group ($F_{1, 32}$ = 4.64, p = .039, η_p^2 = .13): following the initial baseline, the two groups diverged in anxious arousal ratings, with the Active Group showing consistently lowered ratings relative to initial baseline (p's < .05 FDR corrected), while no change was observed in the Sham group (p's > .317; Figure 4A). Importantly, the Active group, but not Sham (p = .607) group, demonstrated sustained baseline shifts across sessions ($F_{2.60, 41.62}$ = 4.76, p = .008, η_p^2 = .23), such that relative to the initial baseline on Day 1, the anxious arousal baseline was consistently lowered on Day 2 (p = .003), Day 3 (p = .021), and Day 4 (p = .005; Figure 4A). Therefore, tACS led to a consistent and lasting reduction in anxious arousal.

Next, to test whether alpha GC enhancement could underpin sustained anxiety reduction, we examined whether the right-hemisphere GC increase on Day 1 (post-30 minutes) could predict anxiety baseline shifts on the following days (relative to the Day 1 baseline). We observed a marginal effect of GC ($F_{1,32}$ = 3.55, p = .069, ηp^2 = .10), which was further qualified by a trending Session-by-GC interaction ($F_{1.93, 62.89}$ = 2.80, p = .071, ηp^2 = .08), suggesting a temporal progression in the association between GC and anxious arousal. Follow-up regression tests for each session revealed that Day 1 GC enhancement predicted reduction in baseline anxious arousal marginally on Day 2 (sr = -.27, p = .082) and significantly on Day 3 (sr = -.41, p = .012, p < .05 FDR corrected), but not on Day 4 (sr = -.10, p = .539; Figure 4B-D).

tACS increased perceived pleasantness of sensory stimuli

Auditory Stimuli

An omnibus rANOVA (Valence, Intensity, Session, Time, and Group) on pleasantness ratings of auditory stimuli revealed a main effect of Group (Active > Sham; $F_{1, 32}$ = 4.19, p

= .049, $\eta_p^2 = .12$), which was further characterized by a Session-by-Time-by-Group interaction ($F_{1.57, 50.17} = 3.84$, $p = .038$, $\eta_p^2 = .11$; Figure 5A). However, this effect did not clearly support increased potency of tACS on affective perception. Follow-up tests for each session showed an interaction of Time-by-Group in the initial ($F_{1.70, 54.28} = 4.10$, $p = .028$, $\eta_p^2 = .11$) but not final ($p = .707$) session. That is, following the initial tACS (but not sham stimulation, $p = .644$), auditory stimuli were perceived as more pleasant ($F_{1.35, 21.59} = 4.10$, $p = .045$, $\eta_p^2 = .20$), immediately ($p = .073$) and 30 minutes ($p = .040$) after stimulation. Although no additional increases were seen after the final stimulation, final baseline ratings were significantly higher than initial baseline ratings, $t = -2.43$, $p = .027$, and remained elevated post-stimulation (immediately, $p = .053$; 30 minutes, $p = .083$), suggesting sustained increases in pleasantness at the final session. No such between- or within-session changes were seen in the Sham group (*p's* > .572). Therefore, sound pleasantness ratings paralleled anxious arousal ratings, increasing after the initial tACS and persisting through the last assessment on Day 4.

Olfactory Stimuli

A similar omnibus rANOVA on pleasantness ratings of olfactory stimuli revealed an interaction of Valence-by-Group ($F_{1, 32} = 8.51$, $p = .006$, $\eta_p^2 = .21$), which was further qualified by a Valence-by-Time-by-Group interaction ($F_{1.75, 55.95} = 5.20$, $p = .011$, $\eta_p^2 = .14$; Figure 5B). Follow-up ANOVAs revealed a Time-by-Group interaction for negative ($F_{1.82, 58.10} = 4.60$, $p = .017$, $\eta_p^2 = .13$) but not neutral ($p = .230$) odor ratings. That is, the Active group showed trending increases in pleasantness of negative odors ($F_{1.88, 30.14} = 3.15$, $p = .060$, $\eta p^2 = .16$), both immediately ($p = .050$) and, marginally, 30 minutes ($p = .066$) after stimulation, paralleled by opposing marginal decreases in pleasantness in the Sham group ($F_{1.72, 27.56} = 3.32$, $p = .058$, ηp^2

= .17). There were no Session-by-Time-by-Group interactions or baseline shifts (p's > .202) to indicate between-session or long-term effects.

DISCUSSION

Combining multi-session α-tACS and multi-wave assessment in an experimental design, we demonstrated immediate and lasting neural and behavioral effects of tACS. α-tACS not only enhanced posterior→frontal alpha-frequency connectivity (in the right hemisphere) immediately and 30 minutes post-stimulation but also shifted the connectivity baseline 24 hours after the preceding stimulation, indicating long-term plasticity. In parallel, anxious arousal decreased and perceived pleasantness of auditory stimuli increased both acutely (immediately and 30 minutes) and 24 hours after α-tACS. By contrast, local posterior alpha power showed transient (immediately and 30 minutes post-stimulation) increases only, as did perceived pleasantness of negative olfactory stimuli. That repeated stimulation failed to induce lasting alpha power enhancement underscores the conservation of local alpha oscillations, contrasting the long-range alpha connectivity that is adaptive to long-term modulation by tACS. Our double-blind, single session replication study with random noise stimulation as the control condition not only replicated the effect of anxiety reduction, but also ruled-out potential confounds of experimenter biases and random electrical stimulation (Supplementary materials).

The acute (immediate and 30-minute) enhancement of local alpha power was evident in both initial and final sessions, echoing previous reports of reliable α-tACS aftereffects (Herrmann *et al.*, 2013; Neuling *et al.*, 2013; Vossen *et al.*, 2015). Furthermore, bin-by-bin examination across the entire frequency spectrum confirmed the specific modulation in the alpha band. In addition, the effect appeared largely in the lower alpha band (8-10.5 Hz), in line with

prior findings of left-ward shifting of alpha peaks by α-tACS (Kasten *et al.*, 2016) and a general trend of higher alpha power being associated with lower alpha peaks (Smit *et al.*, 2006). Transient alpha power enhancement following a single tACS session as previously reported (Kasten *et al.*, 2016) could be attributed to the insufficient stimulation dosage to induce lasting, late LTP to support long-term plasticity. That our protocol of multiple stimulation sessions still failed to generate lasting power increases refuted the account of insufficient dosage, especially in light of lasting alpha connectivity enhancement indicating that the current dosage was sufficient for long-term plasticity. Instead, the transient alpha power increase likely reflected neural entrainment that temporally synchronized occipitoparietal cortical oscillations to exogenous stimulation (Reato *et al.*, 2013; Helfrich *et al.*, 2014). Therefore, as tACS ceased, the highly integrated thalamo-cortical alpha pacemaker (Hughes and Crunelli, 2005; Bazanova and Vernon, 2014) might allow thalamic inputs to restore cortical alpha oscillations to endogenous levels. Overall, the restoration of alpha power, despite repeated stimulation, accentuated the resistance of cortical alpha oscillators to long-term changes. This intrinsic regulation of resting-state alpha oscillations in healthy participants thus implicated a conserved, closed-loop (potentially encapsulated) system of alpha oscillators, critical for preserving neural homeostasis and mental stability (Sterling, 2014).

By contrast, alpha-frequency posterior→frontal connectivity exhibited both immediate and lasting changes, representing some of the first empirical evidence for long-term neural effects of tACS. The immediate effects replicated a recent report of immediate increases in resting-state connectivity between parietal and frontal cortices following α-tACS (Cabral-Calderin *et al.*, 2016). The lasting enhancement in alpha connectivity, in contrast to alpha power, ruled out the possibility that augmented alpha connectivity merely resulted from a potentiated

posterior alpha sender (via enhanced alpha synchrony in the occipitoparietal cortex) or a higher signal-to-noise ratio of scalp EEG at the assessment on Day 4. Rather, the evidence implicated mechanisms beyond local neuronal synchrony, in favor of the interpretation of improved efficiency in alpha projections across large-scale networks, such that with alpha power at the sender remaining constant, directed transmission of alpha oscillations to the distant receiver was intensified.

This improved efficiency in posterior→frontal alpha transmission was likely mediated by synaptic plasticity induced by tACS, via mechanisms such as STDP and LTP (Frohlich and McCormick, 2010; Reato et al., 2010; Zaehle et al., 2010; Polanía et al., 2012; Vossen et al., 2015), which is known to be conducive to enduring Hebbian synaptic plasticity and lasting increases in cortico-cortical oscillatory connectivity (Clopath et al., 2010; Siegel et al., 2012). Since such inter-cortical connectivity (driven by cortical alpha generators; (Rockland and Virga, 1989; Bollimunta et al., 2008)) operates outside the relatively encapsulated thalamo-cortical loop that constrains local cortical oscillations, its plasticity can outlast the effect of local alpha entrainment. This account concurs with the notion that tACS directly modifies interactions across proximal and distant neurons to the extent that the dynamics of macroscopic, global networks are altered (Frohlich and McCormick, 2010; Reato *et al.*, 2010). Here, given the role of alpha oscillations in long-range interactions, α-tACS would generate pronounced connectivity plasticity in long-range pathways. In particular, the posterior→frontal alpha connectivity exhibited maximal gain, akin to its dominance in resting long-range interactions and, critically, tACS stimulation at the posterior site. Connectivity in the opposite direction (frontal→posterior) failed to show acute or delayed effects (p's > .16). This highlights the importance of computing directional measures of connectivity (e.g., GC) such that directional effects would not be

obscured by non-directional measures of connectivity. In addition, to the extent that GC can be influenced by signal power, the fact that GC increases persisted through Day 4 when power had reversed to the baseline largely mitigated this concern.

Paralleling alpha posterior→frontal connectivity increases, anxious arousal decreased both immediately and 24 hours after tACS, which persisted across the four-day period, highlighting reliable and robust effects on anxiety. These anxiety effects outlasted local alpha power enhancement and so were likely underpinned by strengthened posterior→frontal alpha connectivity, which presumably modulated large-scale resting-state networks, especially the DMN and SN that are closely linked to alpha oscillations (Laufs *et al.*, 2003; Buzsáki and Draguhn, 2004; Mantini *et al.*, 2007; Sadaghiani *et al.*, 2010; Mo *et al.*, 2013; Zhan *et al.*, 2014; Tang *et al.*, 2017a) and play critical roles in emotion and arousal (Raichle *et al.*, 2001; Sridharan *et al.*, 2008). The non-clinical nature, and relatively small size (for correlational effects), of the current sample may have occluded the full observation of previously demonstrated direct links between alpha connectivity and hyperarousal in a largely clinical sample (Clancy et al., 2017). However, the fact that neural effects preceded lasting anxiety changes underscores neural plasticity that develops and consolidates over time, giving rise to enduring amelioration of anxiety and related behavioral metrics. Nevertheless, this alpha connectivity enhancement failed to predict anxiety reduction on Day 4, suggesting that neural effects of a single tACS would not sustain prolonged behavioral changes and that multiple sessions would be needed for enduring behavioral effects. Future research with daily resting-state EEG recordings and follow-up assessments (e.g., at 1 week post-treatment) would delineate detailed temporal profiles of neural changes and related behavioral impacts.

Anxiety is often associated with negative stimulus perception (Krusemark and Li, 2011; Forscher and Li, 2012; Krusemark and Li, 2012). Auditory processing is particularly sensitive to states of anxious arousal (Grillon *et al.*, 1991; Braff *et al.*, 2001). In addition, alpha oscillations are known to modulate sensory processing and perception (Palva and Palva, 2007; Foxe and Snyder, 2011; Klimesch *et al.*, 2011; Lou *et al.*, 2014) such that altered alpha activity via α-tACS could further mitigate aversive responses to auditory stimuli by modifying auditory processing. Indeed, immediate and lasting increases in perceived pleasantness of sounds (especially negative sounds) coincided with anxious arousal reduction and alpha connectivity enhancement, providing additional support to our hypotheses. Interestingly, perceived pleasantness of negative odors increased in a transient manner, while lasting increases emerged in the auditory domain. Unlike the auditory system that is neocortical and has direct and close connections with the targeted visual cortex (Falchier *et al.*, 2002; Kayser *et al.*, 2008; Novak *et al.*, 2015), the olfactory system is largely subcortical or paleocortical without direct connections with visual cortex (Carmichael *et al.*, 1994; Haberly, 2001; Krusemark *et al.*, 2013). As such, olfactory perception may be relatively impervious to alpha purtabations with the transient changes (for negative odors) presumably reflecting brief adaptation to internal state changes due to tACS, as a phenomenon of olfactory alliesthesia (Krusemark *et al.*, 2013).

Finally, we explored the possibility of intensified potency of tACS effects over repeated sessions. While there were hints of greater within-session effects on Day 4 relative to Day 1 (Figures 2 & 3), three-way interactions of Session, Time, and Group failed to support this possibility. We surmise that these null effects implicated a biological ceiling among healthy individuals, especially considering the already elevated baselines (for GC connectivity) on Day 4. Similarly, in contrast to clinical patients in a clinical trial, our participants in this proof-of-

concept study had normal levels of baseline anxious arousal (mean scores 32/100), leaving only modest room for anxiety reduction. Nonetheless, anxiety reduced by 34% after tACS on Day 1 and maintained throughout the study, resisting the natural regression to the baseline (as seen in the Sham group). That said, the possibility of tACS potency intensification among patients with reduced alpha activity and greater anxious arousal warrants investigation through double-blind clinical trials, which could provide useful dosage guidance in clinical applications.

Increasing recognition of aberrant oscillatory dynamics in the pathology of various neuropsychiatric disorders has spawned strong interest in directly modulating endogenous oscillations (Schnitzler and Gross, 2005; Uhlhaas and Singer, 2012; Basar, 2013). Meanwhile, network views of psychopathology have gained rapid traction in recent years, advancing conceptualization of mental disorders from local neural aberrations to large-scale circuit anomalies (Kaiser *et al.*, 2015; Clancy *et al.*, 2017; McTeague *et al.*, 2017). In fact, while our lab demonstrated both reduced posterior alpha power and posterior→frontal alpha connectivity in PTSD, we found that only the latter was directly coupled with symptom severity (Clancy *et al.*, 2017). Therefore, the ability of tACS to induce lasting plasticity in long-range connectivity makes this neuromodulatory technique especially advantageous for treating disorders rooted in pathological oscillatory networks. While meditation and neurofeedback can modulate resting-state networks and reduce anxiety and arousal, along with alpha power enhancement, (Hammond, 2005; Cahn and Polich, 2006; Ros *et al.*, 2013; Fan *et al.*, 2014; Tang *et al.*, 2017b), their long-term impact on alpha-frequency connectivity remain unclear. In this regard, we call for attention to oscillatory connectivity modification and network adaptation in future investigation of all forms of neuromodulation.

FIGURE CAPTIONS

Figure 1. **Stimulation setup and experimental protocol.** A) Top: tACS montage, with stimulation electrodes placed over occipitoparietal sites. Bottom: Modeled current flow of tACS, demonstrating maximal electrical field intensity over the occipitoparietal cortex. B) Participants completed 4 sessions of tACS or sham stimulation, each separated by 24 hours. tACS was administered for 30 minutes at each individual's peak alpha frequency. Eyes-open resting state EEG data and ratings of anxious arousal and perceived pleasantness of auditory and olfactory stimuli were acquired before, immediately and 30 minutes after stimulation.

Figure 2. **Changes in posterior alpha power.** A) Spectra of normalized power (collapsed across occipitoparietal electrodes) for each group at the three time points, averaged across the initial and final sessions. The yellow line at the top of the waveforms indicates frequency bins (.25 Hz each) showing a significant Time-by-Group interaction. The interaction effect appeared selectively in the lower half of the targeted alpha range (8-10.5 Hz), confirming the specific modulation of alpha power. Note, the Active group demonstrated post-stimulation increases in the alpha band from the baseline while the Sham group demonstrated equivalent alpha power before and after stimulation. B) Alpha power magnitudes, decibel-normalized to the initial baseline, for each group. C) Scalp topographical maps of normalized alpha power at the initial baseline (surrounded by a black box and associated with its own color scale), and decibel-normalized power changes at each assessment thereafter. Electrodes included in occipitoparietal sites are bolded and encircled. Error bars = SEM. *$p < .05$; **$p < .01$; *** $p < .005$. All *p*'s are relative to the initial baseline. Note, the groups did not differ in the initial baseline values (*p*'s > .19).

Figure 3. **Changes in posterior→frontal GC.** A) Spectra of right-hemisphere posterior→frontal Granger causality for each group (averaged across time for simplicity). The yellow line at the top of the waveforms indicates frequency bins (.25 Hz each) showing a significant Session-by-Group interaction, which spans almost the entire alpha band (8.25-12 Hz) but no other frequency band, confirming the specific modulation of alpha-frequency GC. B) Magnitudes of right posterior→frontal alpha GC, decibel-normalized to initial baseline, for each group. C) Scalp topographical maps of alpha GC sent from right occipitoparietal electrodes at the initial baseline (surrounded by a black box and associated with its own color scale), and decibel-normalized changes at each assessment thereafter. Sending occipitoparietal and receiving frontal electrodes are bolded and encircled. Error bars = SEM. *$p < .05$; **$p < .01$; *** $p < .005$. All p's are relative to the initial baseline. Note, the groups did not differ in the initial baseline values (p's > .19).

Figure 4. **Anxious arousal outcome.** A) Anxious arousal ratings for each group at each assessment over the four sessions/days. B-D) Day 1 (initial session) GC change at post-30 minutes predicted anxious arousal baseline shift (after controlling for individual differences in pre-study trait anxiety/BIS scores) on Day 2 (second session; B) and Day 3 (third session; C), but the association greatly declined on Day 4 (final session; D). Error bars = adjusted standard error (SEE). *$p < .05$; **$p < .01$; † $p < .1$. All p's are relative to the initial baseline. The groups did not differ in the initial baseline ($p = .718$).

Figure 5. **Pleasantness ratings of neutral and negative A) sounds and B) odors.** Neutral valence = 50. Error bars = SEM. **p* < .05; ***p* < .01; † *p* < .1. All *p*'s are relative to the initial baseline. The groups did not differ in the initial baseline ratings (*p*'s > .215).

FIGURES

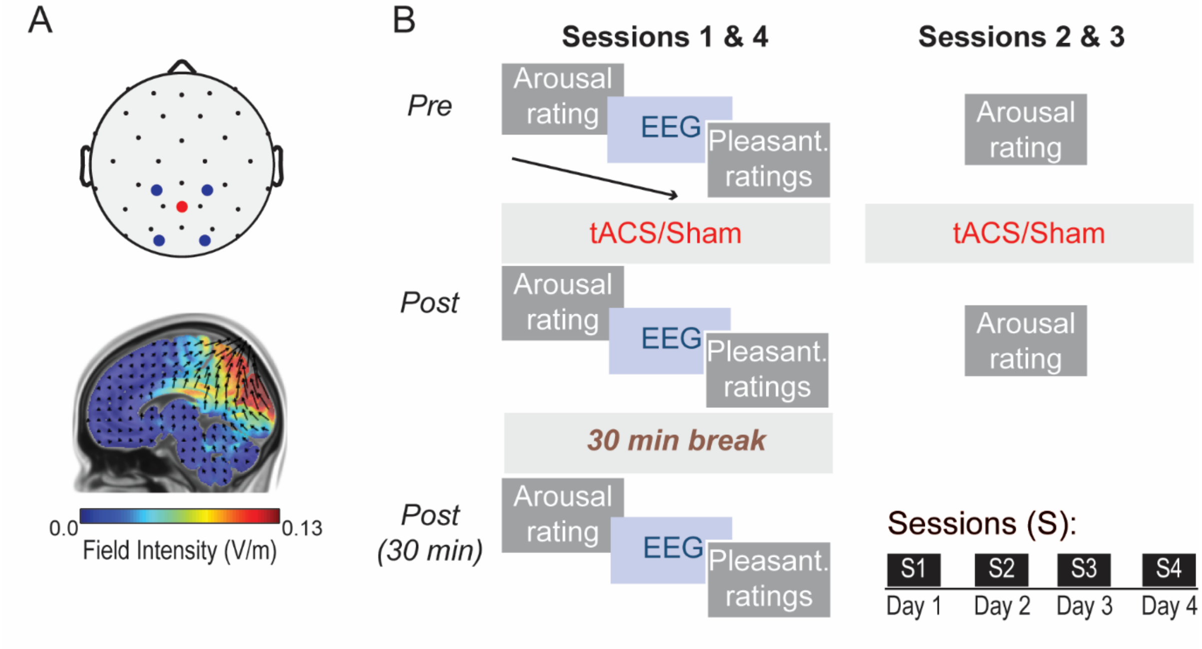

Figure 1.

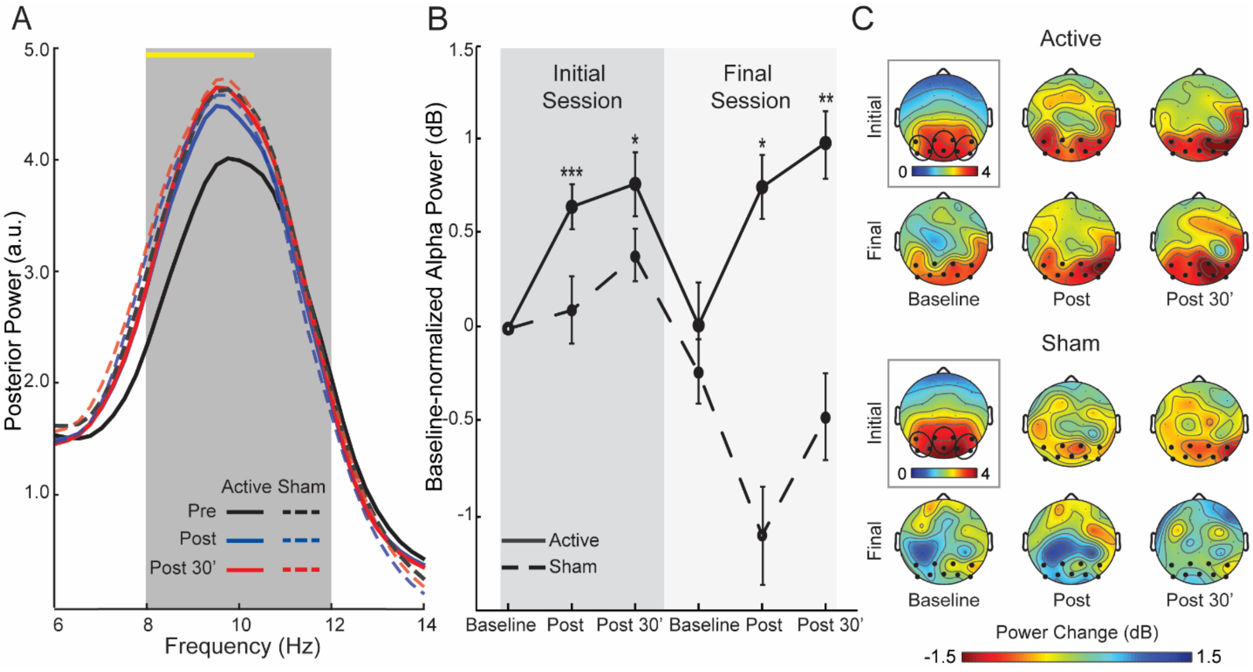

Figure 2.

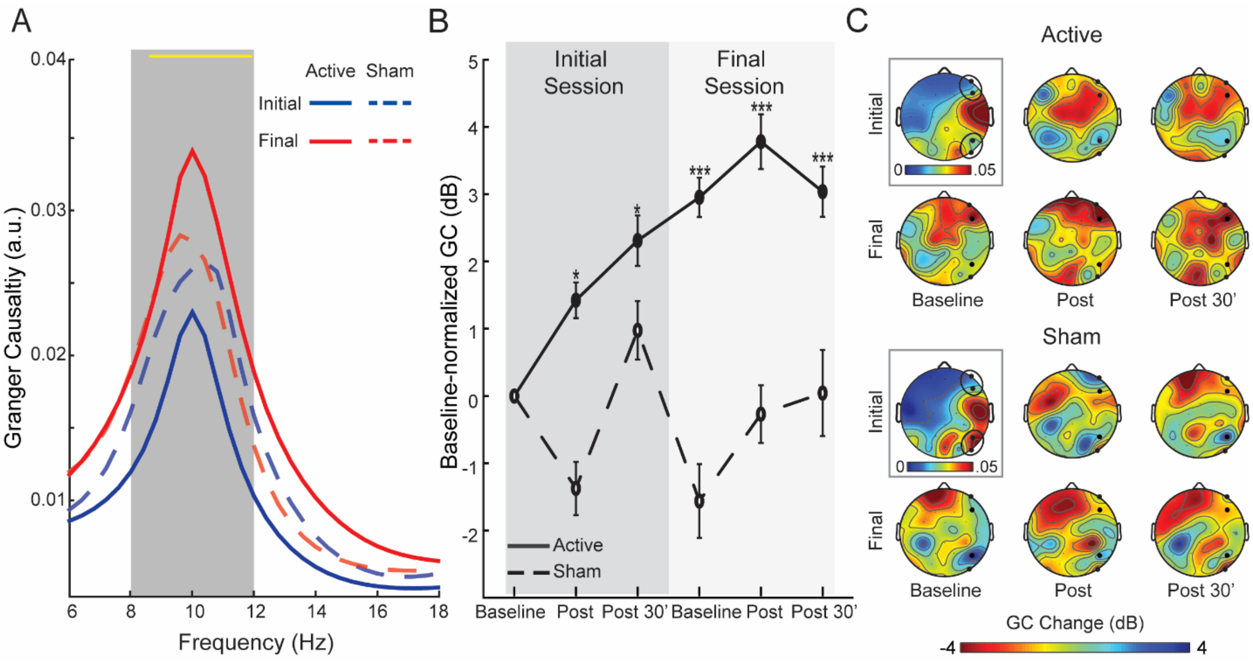

Figure 3.

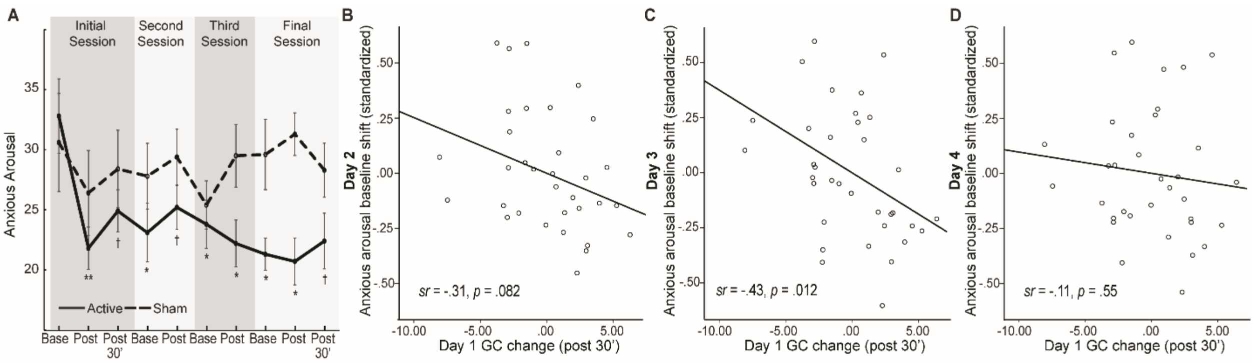

Figure 4.

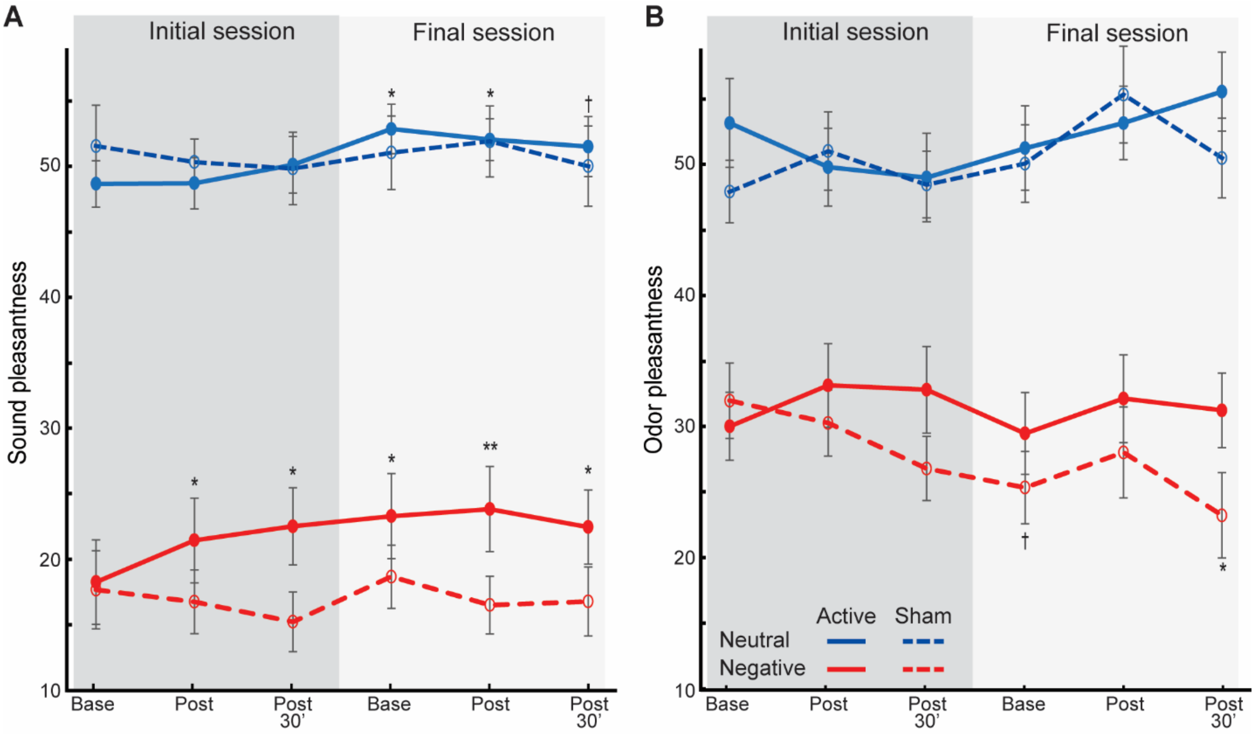
A
Initial session
Final session
Sound pleasantness
10
20
30
40
50
Base
Post
Post 30'
Base
Post
Post 30'
B
Initial session
Final session
Odor pleasantness
Active
Sham
Neutral
Negative

Figure 5.

Supplementary Materials

SUPPLEMENTARY METHODS

Sensory Stimuli

Intensity levels for presented stimuli are presented below in Supplemental Table 1. All olfactory stimuli were created using 1/3 serial dilutions from a 5% base using mineral oil.

Behavioral Inhibition Scale (BIS)

The BIS (Carver and White, 1994) consists of 7 items assessing behavioral avoidance and trait anxiety using a 4 point Likert scale to indicate how much each individual disagrees (1) or agrees (4) with each statement (e.g. "If I think something unpleasant is going to happen I usually get pretty worked up"). This trait anxiety measure was collected at the beginning of the first session and was entered into our statistical analyses as a covariate to control for individual differences in trait anxiety. Note, the groups did not differ on BIS scores (p = .22).

Adverse Effects Questionnaire (AEQ)

The AEQ (Brunoni *et al.*, 2011) consists of 10 items assessing the experience of common transcranial electrical stimulation symptoms or side effects using a 4 point Likert scale to index the severity of each symptom (1 = absent, 4 = severe) and a 5 point Likert scale to index the likelihood these sensations are related to the stimulation (1 = none, 5 = definite). Across both groups, the most common side-effects experienced were scalp tingling (52.6%) and feelings of sleepiness (47.4%). All participants endorsing scalp tingling attributed it to the stimulation, whereas 83.5% of those endorsing sleepiness thought it was related to the stimulation. Group

differences in the experience of tACS-induced symptoms or side effects emerged only in the presence of scalp burning and the ascribed likelihood that sensations of scalp pain and burning could be attributed to the tACS administration. Importantly, compared to the Active group (*M* = 1.00, *SD* = 0.00—Active participants uniformly indicated no scalp burning), the Sham group (*M* = 1.35, *SD* = .77) indicated increased severity of scalp burning, $t_{34} = -3.13$, $p = .004$. Additionally, the Sham group attributed the sensations of scalp pain (*M* = 2.53, *SD* = 1.66) and burning (*M* = 2.00, *SD* = 1.54) to the perceived stimulation more so than the Active group (pain: *M* = 1.47, *SD* = 1.17; burning: *M* = 1.00, *SD* = 0.00—Active participants uniformly denied scalp burning as a result of stimulation), *t*'s < -.220, *p*'s < .033. The groups did not differ in ratings of any other symptoms (*p*'s > .114). Therefore, these results corroborated subject report during debriefing, further confirming that the sham group was not aware of the sham stimulation.

SUPPLEMENTARY RESULTS

Double-blind Replication Study

To rule out potential experimenter biases, we conducted a double-blind, single-session replication study on a smaller scale. Additionally, we applied random noise stimulation (tRNS; 1-200 Hz) as an active control in this study to remove random electrical stimulation effects. Eighteen participants (n = 9 per group) were recruited for the replication, based on power analyses (power = .81 with n =9 and Cohen's *d* = .92; one-tailed t-test). Participants completed the same visual analog scale (VAS) anxiety rating immediately before and after 30 minutes of tACS or tRNS. The two groups did not differ in baseline, pre-stimulation anxious arousal ($t = 1.22$, $p = .239$). Given the hypothesized direction of effects (i.e. reductions in anxious arousal),

we applied one-tailed tests. Similar to the initial study, BIS scores were included as a covariate to control for individual differences in trait-level anxiety.

An rANOVA of Time (Pre vs. Post) and Group (tACS/Active, tRNS/Control) on anxious arousal revealed a Time x Group interaction ($F_{1, 15}$ = 4.29, p = .028 one tailed, ηp^2 = .22), consistent with the effect size found in the main experiment. Follow-up contrasts within each group revealed a reduction in anxious arousal in the tACS group (t = 1.96, p = .043 one-tailed), but not the tRNS group (t = -1.01, p = .172 one-tailed). Therefore, these findings replicated our initial study and ruled out possible confounds of experimenter biases and random stimulation.

Associations between changes in alpha power and posterior→frontal connectivity

We performed supplemental analyses of the relationship between changes in alpha power and GC following stimulation (collapsed across the two post-stimulation sessions), especially concerning whether one index would predict the other from Day 1 to Day 4. Day 1 alpha power increases did not predict GC changes on Day 4 (r's < .05, p's > .788). In comparison, Day 1 increases in GC marginally predicted Day 4 increases in power (r = .31, p = .055) although not the baseline shift (r = .19, p = .249), which could be related to the fact that Day 4 baseline power had returned to the Day 1 baseline. Together, these results suggest that alpha connectivity enhancement was unlikely driven by alpha power augmentation, in keeping with the notion that tACS takes effect via cortico-cortical oscillatory reverberation and synaptic plasticity (Alagapan et al., 2016).

Changes in connectivity increases were associated with greater increases in perceived pleasantness of negative sounds between sessions

As suggested by one of the reviewers, we further examined correlations in double differences [Day 4 (post – pre) – Day1 (post – pre)] between alpha activity and behavior. We observed a significant correlation between double-differences in GC and perceived pleasantness of negative sounds ($r = .38$, $p = .028$) but not in double-differences between GC and anxiety (p's > .54) or perceived pleasantness of neutral sounds ($p = .48$). No such effects were seen with double-differences in pleasantness of neutral or negative odors (p's > .10; Supplemental Table 3), either. As reported in the main text, the direct effect of Day 1 GC changes on behavior (i.e., anxiety) was observed on Days 2 & 3 but not on Day 4. The overall lack of correlation between Days 1 and 4 is consistent with the duration (up to 2 days) of long-term effects of tACS.

Increases in connectivity were not related to increases in perceived pleasantness of auditory stimuli, neither prospectively nor concurrently

In the interest of thoroughness, we evaluated whether changes in alpha power or connectivity were related to changes in perceived pleasantness of sensory stimuli. No such concurrent associations emerged (p's > .211; Supplemental Tables 4 and 5).

SUPPLEMENTAL TABLES

Supplemental Table 1. Intensities of presented sensory stimuli at weak, medium, and strong levels.

	Weak	Medium	Strong
Sound – neutral	47 dB	50 dB	55 dB
Sound – scream	47 dB	54 dB	67 dB
Sound – vomit	50 dB	58 dB	67 dB
Odor – neutral	.54%	1.65%	5%
Odor – negative	.54%	1.65%	5%

Auditory stimuli intensities are presented in decibel readings from headphones, and olfactory stimuli are presented in concentrations of pure base odorant.

Supplemental Table 2. Correlations between Day 1 concurrent and Day 4 changes in alpha power and connectivity.

	Day 1 Post-stim rGC	Day 1 Post-stim alpha	Day 4 Baseline rGC	Day 4 Post-stim rGC	Day 4 Baseline power	Day 4 Post-stim power
Day 1 Post-stim rGC		.29	.51***	.68***	.19	.31†
Day 1 Post-stim power			-.03	-.05	.20	.50***

rGC = right-hemisphere bottom-up alpha Granger causality. Power = alpha power. ***$p < .001$; † $p < .1$

Supplemental Table 3. Correlations between differences in alpha power and connectivity between Day 4 and Day 1.

	Anxiety Delta change	Neutral sound Delta change	Negative sound Delta change	Neutral odor Delta change	Negative odor Delta change
Power Delta change	-.19	-.04	.17	-.27	-.29
rGC Delta change	-.11	.13	.38*	-.04	-.05

rGC = right-hemisphere bottom-up alpha Granger causality. Power = alpha power. * $p < .05$.

Supplemental Table 4. Correlations between changes in alpha power and connectivity and changes in pleasantness ratings of auditory stimuli.

	Sess. 1 Post-stim Neutral Sound	Sess. 1 Post-stim Negative Sound	Sess. 4 Baseline Neutral Sound	Sess. 4 Baseline Negative sound	Sess. 4 Post-stim Neutral Sound	Sess. 4 Post-stim Negative Sound
Sess. 1 Post-stim power	.17	.24	-.15	-.17	.28	.19
Sess. 1 Post-stim rGC	.03	.12	.17	-.03	.18	.06
Sess. 4 Baseline power	.09	-.01	-.06	-.03	-.09	-.01
Sess. 4 Baseline rGC	.07	.24	.03	.17	.05	.15
Sess. 4 Post-stim power	.14	.04	.13	-.20	.11	.05
Sess. 4 Post-stim rGC	-.02	-.04	.08	-.03	.17	-.03

rGC = right-hemisphere bottom-up alpha Granger causality. Power = alpha power.

Supplemental Table 5. Correlations between changes in alpha power and connectivity and changes in pleasantness ratings of olfactory stimuli.

	Sess. 1 Post-stim Neutral odor	Sess. 1 Post-stim Negative odor	Sess. 4 Baseline Neutral odor	Sess. 4 Baseline Negative odor	Sess. 4 Post-stim Neutral odor	Sess. 4 Post-stim Negative odor
Sess. 1 Post-stim power	-.15	-.19	-.15	-.15	-.09	-.05
Sess. 1 Post-stim rGC	-.22	.06	-.21	-.04	-.19	-.01
Sess. 4 Baseline power	-.07	.17	-.06	-.02	-.09	-.05
Sess. 4 Baseline rGC	-.24	.19	-.17	.08	-.21	.02
Sess. 4 Post-stim power	-.09	-.01	-.06	-.10	-.18	-.16
Sess. 4 Post-stim rGC	-.15	.09	-.21	-.13	-.23	-.16

rGC = right-hemisphere bottom-up alpha Granger causality. Power = alpha power.

SUPPLEMENTAL FIGURES

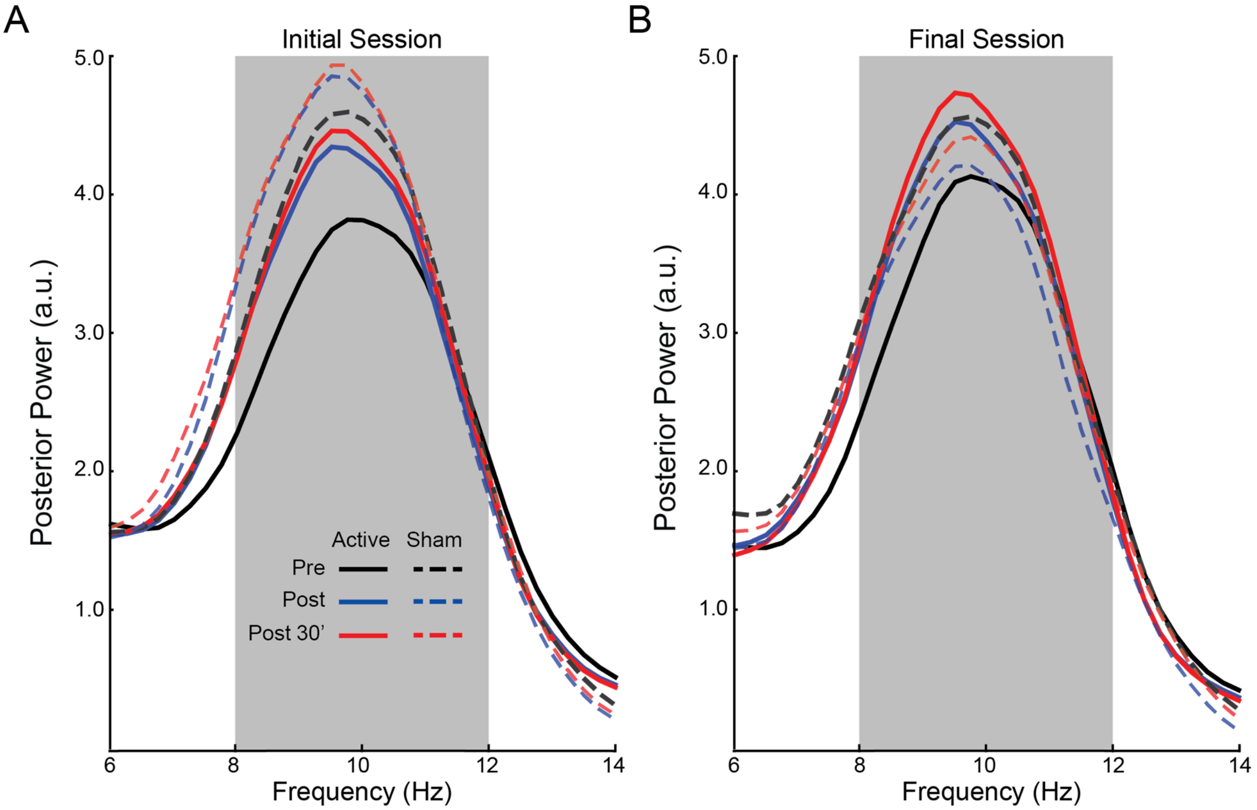

Figure S1. Raw power spectra from occipitoparietal electrodes for all time points (pre-, post-, post-30 minutes stimulation) for the A) initial and B) final sessions from the main experiment. This figure represents an expansion of Figure 2A in the main text, which was collapsed across sessions to demonstrate the significant Time-by-Condition interaction.

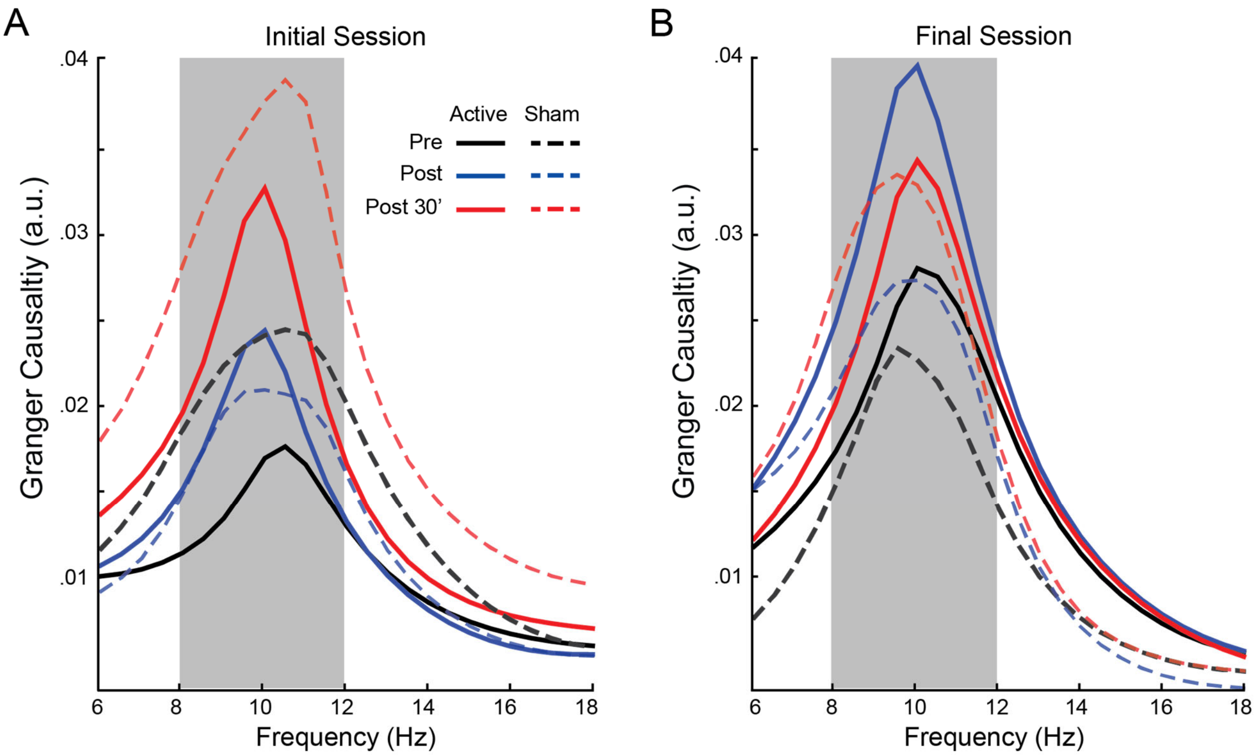

Figure S2. Raw spectra of right-hemisphere posterior→frontal Granger causality for each group for all time points (pre-, post-, post-30 minutes stimulation) for the A) initial and B) final sessions from the main experiment. This figure represents an expansion of Figure 3A in the main text, which was collapsed across time to demonstrate the significant Session-by-Condition interaction.

SUPPLEMENTARY REFERENCES

Brunoni, A. R., Amadera, J., Berbel, B., Volz, M. S., Rizzerio, B. G. & Fregni, F. (2011) 'A systematic review on reporting and assessment of adverse effects associated with transcranial direct current stimulation', *Int J Neuropsychopharmacol*, **14**(8), pp. 1133-1145.

Carver, C. S. & White, T. L. (1994) 'Behavioral-Inhibition, Behavioral Activation, and Affective Responses to Impending Reward and Punishment - the Bis Bas Scales', *Journal of Personality and Social Psychology*, **67**(2), pp. 319-333.

REFERENCES

Abend, R., Jalon, I., Gurevitch, G., Sar-El, R., Shechner, T., Pine, D. S., Hendler, T. & Bar-Haim, Y. (2016) 'Modulation of fear extinction processes using transcranial electrical stimulation', *Transl Psychiatry*, **6**(10), p. e913.
Abraham, W. C. (2003) 'How long will long-term potentiation last?', *Philos Trans R Soc Lond B Biol Sci*, **358**(1432), pp. 735-744.
Adler, L. E., Pang, K., Gerhardt, G. & Rose, G. M. (1988) 'Modulation of the gating of auditory evoked potentials by norepinephrine: pharmacological evidence obtained using a selective neurotoxin', *Biol Psychiatry*, **24**(2), pp. 179-190.
Alagapan, S., Schmidt, S. L., Lefebvre, J., Hadar, E., Shin, H. W. & Frohlich, F. (2016) 'Modulation of Cortical Oscillations by Low-Frequency Direct Cortical Stimulation Is State-Dependent', *PLoS Biol*, **14**(3), p. e1002424.
Alvarez, R. P., Kirlic, N., Misaki, M., Bodurka, J., Rhudy, J. L., Paulus, M. P. & Drevets, W. C. (2015) 'Increased anterior insula activity in anxious individuals is linked to diminished perceived control', *Transl Psychiatry*, **5**, p. e591.
Antal, A. & Paulus, W. (2013) 'Transcranial alternating current stimulation (tACS)', *Frontiers in human neuroscience*, **7**.
Basar, E. (2013) 'Brain oscillations in neuropsychiatric disease', *Dialogues Clin Neurosci*, **15**(3), pp. 291-300.
Başar, E., Başar-Eroglu, C., Karakaş, S. & Schürmann, M. (2001) 'Gamma, alpha, delta, and theta oscillations govern cognitive processes', *International journal of psychophysiology*, **39**(2), pp. 241-248.
Bazanova, O. M. & Vernon, D. (2014) 'Interpreting EEG alpha activity', *Neurosci Biobehav Rev*, **44**, pp. 94-110.
Bollimunta, A., Chen, Y., Schroeder, C. E. & Ding, M. (2008) 'Neuronal mechanisms of cortical alpha oscillations in awake-behaving macaques', *J Neurosci*, **28**(40), pp. 9976-9988.
Braff, D. L., Geyer, M. A. & Swerdlow, N. R. (2001) 'Human studies of prepulse inhibition of startle: normal subjects, patient groups, and pharmacological studies', *Psychopharmacology*, **156**(2-3), pp. 234-258.
Brunoni, A. R., Amadera, J., Berbel, B., Volz, M. S., Rizzerio, B. G. & Fregni, F. (2011) 'A systematic review on reporting and assessment of adverse effects associated with transcranial direct current stimulation', *Int J Neuropsychopharmacol*, **14**(8), pp. 1133-1145.
Buzsáki, G. & Draguhn, A. (2004) 'Neuronal oscillations in cortical networks', *science*, **304**(5679), pp. 1926-1929.
Buzsaki, G., Logothetis, N. & Singer, W. (2013) 'Scaling brain size, keeping timing: evolutionary preservation of brain rhythms', *Neuron*, **80**(3), pp. 751-764.
Cabral-Calderin, Y., Williams, K. A., Opitz, A., Dechent, P. & Wilke, M. (2016) 'Transcranial alternating current stimulation modulates spontaneous low frequency fluctuations as measured with fMRI', *Neuroimage*, **141**, pp. 88-107.
Cahn, B. R. & Polich, J. (2006) 'Meditation states and traits: EEG, ERP, and neuroimaging studies', *Psychol Bull*, **132**(2), pp. 180-211.
Carmichael, S. T., Clugnet, M. C. & Price, J. L. (1994) 'Central olfactory connections in the macaque monkey', *Journal of Comparative Neurology*, **346**(3), pp. 403-434.

Carver, C. S. & White, T. L. (1994) 'Behavioral-Inhibition, Behavioral Activation, and Affective Responses to Impending Reward and Punishment - the Bis Bas Scales', *Journal of Personality and Social Psychology*, **67**(2), pp. 319-333.
Clancy, K., Ding, M., Bernat, E., Schmidt, N. B. & Li, W. (2017) 'Restless 'rest': intrinsic sensory hyperactivity and disinhibition in post-traumatic stress disorder', *Brain*, **140**(7), pp. 2041-2050.
Clopath, C., Büsing, L., Vasilaki, E. & Gerstner, W. (2010) 'Connectivity reflects coding: a model of voltage-based STDP with homeostasis', *Nature neuroscience*, **13**(3), pp. 344-352.
Cohen, M. R. & Maunsell, J. H. (2010) 'A neuronal population measure of attention predicts behavioral performance on individual trials', *J Neurosci*, **30**(45), pp. 15241-15253.
Cohen, M. X. (2014) 'Analyzing neural time series data : theory and practice'.
Corbetta, M. & Shulman, G. L. (2011) 'Spatial neglect and attention networks', *Annu Rev Neurosci*, **34**, pp. 569-599.
Ding, M., Bressler, S. L., Yang, W. & Liang, H. (2000) 'Short-window spectral analysis of cortical event-related potentials by adaptive multivariate autoregressive modeling: data preprocessing, model validation, and variability assessment', *Biol Cybern*, **83**(1), pp. 35-45.
Ding, M., Chen, Y. & Bressler, S. L. (2006) 'Granger Causality: Basic Theory and Application to Neuroscience', *Handbook of Time Series Analysis*, Wiley-VCH Verlag GmbH & Co. KGaA.
Dugue, L., Marque, P. & VanRullen, R. (2011) 'The phase of ongoing oscillations mediates the causal relation between brain excitation and visual perception', *J Neurosci*, **31**(33), pp. 11889-11893.
Engel, A. K., Fries, P. & Singer, W. (2001) 'Dynamic predictions: oscillations and synchrony in top-down processing', *Nat Rev Neurosci*, **2**(10), pp. 704-716.
Falchier, A., Clavagnier, S., Barone, P. & Kennedy, H. (2002) 'Anatomical evidence of multimodal integration in primate striate cortex', *J Neurosci*, **22**(13), pp. 5749-5759.
Fan, Y., Tang, Y. Y., Tang, R. & Posner, M. I. (2014) 'Short term integrative meditation improves resting alpha activity and stroop performance', *Appl Psychophysiol Biofeedback*, **39**(3-4), pp. 213-217.
Forscher, E. C. & Li, W. (2012) 'Hemispheric asymmetry and visuo-olfactory integration in perceiving subthreshold (micro) fearful expressions', *J Neurosci*, **32**(6), pp. 2159-2165.
Foxe, J. J. & Snyder, A. C. (2011) 'The Role of Alpha-Band Brain Oscillations as a Sensory Suppression Mechanism during Selective Attention', *Front Psychol*, **2**, p. 154.
Fregni, F., Otachi, P. T., Do Valle, A., Boggio, P. S., Thut, G., Rigonatti, S. P., Pascual-Leone, A. & Valente, K. D. (2006) 'A randomized clinical trial of repetitive transcranial magnetic stimulation in patients with refractory epilepsy', *Ann Neurol*, **60**(4), pp. 447-455.
Frohlich, F. (2015) 'Experiments and models of cortical oscillations as a target for noninvasive brain stimulation', *Prog Brain Res*, **222**, pp. 41-73.
Frohlich, F. & McCormick, D. A. (2010) 'Endogenous electric fields may guide neocortical network activity', *Neuron*, **67**(1), pp. 129-143.
Gath, I., Lehmann, D. & Bar-On, E. (1983) 'Fuzzy clustering of EEG signal and vigilance performance', *Int J Neurosci*, **20**(3-4), pp. 303-312.
Geweke, J. (1982) 'Measurement of linear dependence and feedback between multiple time series', *Journal of the American statistical association*, **77**(378), pp. 304-313.
Grillon, C., Ameli, R., Woods, S. W., Merikangas, K. & Davis, M. (1991) 'Fear-potentiated startle in humans: Effects of anticipatory anxiety on the acoustic blink reflex', *Psychophysiology*, **28**(5), pp. 588-595.

Haberly, L. B. (2001) 'Parallel-distributed processing in olfactory cortex: new insights from morphological and physiological analysis of neuronal circuitry', *Chemical senses*, **26**(5), pp. 551-576.

Hammond, D. C. (2005) 'Neurofeedback with anxiety and affective disorders', *Child Adolesc Psychiatr Clin N Am*, **14**(1), pp. 105-123, vii.

Helfrich, R. F., Schneider, T. R., Rach, S., Trautmann-Lengsfeld, S. A., Engel, A. K. & Herrmann, C. S. (2014) 'Entrainment of brain oscillations by transcranial alternating current stimulation', *Curr Biol*, **24**(3), pp. 333-339.

Herrmann, C. S., Rach, S., Neuling, T. & Struber, D. (2013) 'Transcranial alternating current stimulation: a review of the underlying mechanisms and modulation of cognitive processes', *Front Hum Neurosci*, **7**, p. 279.

Hillebrand, A., Tewarie, P., van Dellen, E., Yu, M., Carbo, E. W., Douw, L., Gouw, A. A., van Straaten, E. C. & Stam, C. J. (2016) 'Direction of information flow in large-scale resting-state networks is frequency-dependent', *Proc Natl Acad Sci U S A*, **113**(14), pp. 3867-3872.

Huang, Y.-Z., Lu, M.-K., Antal, A., Classen, J., Nitsche, M., Ziemann, U., Ridding, M., Hamada, M., Ugawa, Y. & Jaberzadeh, S. (2017) 'Plasticity induced by non-invasive transcranial brain stimulation: a position paper', *Clinical Neurophysiology*.

Hughes, S. W. & Crunelli, V. (2005) 'Thalamic mechanisms of EEG alpha rhythms and their pathological implications', *Neuroscientist*, **11**(4), pp. 357-372.

Johnson, E. L., Dewar, C. D., Solbakk, A. K., Endestad, T., Meling, T. R. & Knight, R. T. (2017) 'Bidirectional Frontoparietal Oscillatory Systems Support Working Memory', *Curr Biol*, **27**(12), pp. 1829-1835 e1824.

Kaiser, R. H., Andrews-Hanna, J. R., Wager, T. D. & Pizzagalli, D. A. (2015) 'Large-scale network dysfunction in major depressive disorder: a meta-analysis of resting-state functional connectivity', *JAMA Psychiatry*, **72**(6), pp. 603-611.

Kasten, F. H., Dowsett, J. & Herrmann, C. S. (2016) 'Sustained Aftereffect of α-tACS Lasts Up to 70 min after Stimulation', *Frontiers in human neuroscience*, **10**.

Kayser, C., Petkov, C. I. & Logothetis, N. K. (2008) 'Visual modulation of neurons in auditory cortex', *Cereb Cortex*, **18**(7), pp. 1560-1574.

Klimesch, W. (1999) 'EEG alpha and theta oscillations reflect cognitive and memory performance: a review and analysis', *Brain Res Brain Res Rev*, **29**(2-3), pp. 169-195.

Klimesch, W. (2012) 'alpha-band oscillations, attention, and controlled access to stored information', *Trends Cogn Sci*, **16**(12), pp. 606-617.

Klimesch, W., Fellinger, R. & Freunberger, R. (2011) 'Alpha oscillations and early stages of visual encoding', *Frontiers in psychology*, **2**.

Krusemark, E. A. & Li, W. (2011) 'Do all threats work the same way? Divergent effects of fear and disgust on sensory perception and attention', *J Neurosci*, **31**(9), pp. 3429-3434.

Krusemark, E. A. & Li, W. (2012) 'Enhanced olfactory sensory perception of threat in anxiety: an event-related fMRI study', *Chemosensory perception*, **5**(1), pp. 37-45.

Krusemark, E. A., Novak, L. R., Gitelman, D. R. & Li, W. (2013) 'When the sense of smell meets emotion: anxiety-state-dependent olfactory processing and neural circuitry adaptation', *J Neurosci*, **33**(39), pp. 15324-15332.

Laufs, H., Krakow, K., Sterzer, P., Eger, E., Beyerle, A., Salek-Haddadi, A. & Kleinschmidt, A. (2003) 'Electroencephalographic signatures of attentional and cognitive default modes in spontaneous brain activity fluctuations at rest', *Proc Natl Acad Sci U S A*, **100**(19), pp. 11053-11058.

Lou, B., Li, Y., Philiastides, M. G. & Sajda, P. (2014) 'Prestimulus alpha power predicts fidelity of sensory encoding in perceptual decision making', *Neuroimage*, **87**, pp. 242-251.
Mantini, D., Perrucci, M. G., Del Gratta, C., Romani, G. L. & Corbetta, M. (2007) 'Electrophysiological signatures of resting state networks in the human brain', *Proceedings of the National Academy of Sciences*, **104**(32), pp. 13170-13175.
Mathewson, K. E., Gratton, G., Fabiani, M., Beck, D. M. & Ro, T. (2009) 'To see or not to see: prestimulus alpha phase predicts visual awareness', *J Neurosci*, **29**(9), pp. 2725-2732.
McTeague, L. M., Huemer, J., Carreon, D. M., Jiang, Y., Eickhoff, S. B. & Etkin, A. (2017) 'Identification of Common Neural Circuit Disruptions in Cognitive Control Across Psychiatric Disorders', *American Journal of Psychiatry*, pp. appi-ajp.
Menon, V. & Uddin, L. Q. (2010) 'Saliency, switching, attention and control: a network model of insula function', *Brain Struct Funct*, **214**(5-6), pp. 655-667.
Mitra, P. P. & Pesaran, B. (1999) 'Analysis of dynamic brain imaging data', *Biophys J*, **76**(2), pp. 691-708.
Mo, J., Liu, Y., Huang, H. & Ding, M. (2013) 'Coupling between visual alpha oscillations and default mode activity', *Neuroimage*, **68**, pp. 112-118.
Moisa, M., Polania, R., Grueschow, M. & Ruff, C. C. (2016) 'Brain network mechanisms underlying motor enhancement by transcranial entrainment of gamma oscillations', *Journal of Neuroscience*, **36**(47), pp. 12053-12065.
Neuling, T., Rach, S. & Herrmann, C. S. (2013) 'Orchestrating neuronal networks: sustained after-effects of transcranial alternating current stimulation depend upon brain states', *Front Hum Neurosci*, **7**, p. 161.
Neuling, T., Wagner, S., Wolters, C. H., Zaehle, T. & Herrmann, C. S. (2012) 'Finite-element model predicts current density distribution for clinical applications of tDCS and tACS', *Frontiers in psychiatry*, **3**.
Nolan, H., Whelan, R. & Reilly, R. B. (2010) 'FASTER: Fully Automated Statistical Thresholding for EEG artifact Rejection', *J Neurosci Methods*, **192**(1), pp. 152-162.
Novak, L. R., Gitelman, D. R., Schuyler, B. & Li, W. (2015) 'Olfactory-visual integration facilitates perception of subthreshold negative emotion', *Neuropsychologia*, **77**, pp. 288-297.
Nunez, P. L., Srinivasan, R., Westdorp, A. F., Wijesinghe, R. S., Tucker, D. M., Silberstein, R. B. & Cadusch, P. J. (1997) 'EEG coherency. I: Statistics, reference electrode, volume conduction, Laplacians, cortical imaging, and interpretation at multiple scales', *Electroencephalogr Clin Neurophysiol*, **103**(5), pp. 499-515.
Palva, S. & Palva, J. M. (2007) 'New vistas for alpha-frequency band oscillations', *Trends Neurosci*, **30**(4), pp. 150-158.
Perrin, F., Pernier, J., Bertrand, O. & Echallier, J. F. (1989) 'Spherical splines for scalp potential and current density mapping', *Electroencephalography and clinical neurophysiology*, **72**(2), pp. 184-187.
Peterson, A., Thome, J., Frewen, P. & Lanius, R. A. (2014) 'Resting-state neuroimaging studies: a new way of identifying differences and similarities among the anxiety disorders?', *Can J Psychiatry*, **59**(6), pp. 294-300.
Polanía, R., Nitsche, M. A., Korman, C., Batsikadze, G. & Paulus, W. (2012) 'The importance of timing in segregated theta phase-coupling for cognitive performance', *Current Biology*, **22**(14), pp. 1314-1318.
Raichle, M. E., MacLeod, A. M., Snyder, A. Z., Powers, W. J., Gusnard, D. A. & Shulman, G. L. (2001) 'A default mode of brain function', *Proc Natl Acad Sci U S A*, **98**(2), pp. 676-682.

Rajagovindan, R. & Ding, M. (2011) 'From prestimulus alpha oscillation to visual-evoked response: an inverted-U function and its attentional modulation', *J Cogn Neurosci*, **23**(6), pp. 1379-1394.
Reato, D., Rahman, A., Bikson, M. & Parra, L. C. (2010) 'Low-intensity electrical stimulation affects network dynamics by modulating population rate and spike timing', *J Neurosci*, **30**(45), pp. 15067-15079.
Reato, D., Rahman, A., Bikson, M. & Parra, L. C. (2013) 'Effects of weak transcranial alternating current stimulation on brain activity-a review of known mechanisms from animal studies', *Front Hum Neurosci*, **7**, p. 687.
Reis, J., Schambra, H. M., Cohen, L. G., Buch, E. R., Fritsch, B., Zarahn, E., Celnik, P. A. & Krakauer, J. W. (2009) 'Noninvasive cortical stimulation enhances motor skill acquisition over multiple days through an effect on consolidation', *Proc Natl Acad Sci U S A*, **106**(5), pp. 1590-1595.
Rockland, K. S. & Virga, A. (1989) 'Terminal arbors of individual "feedback" axons projecting from area V2 to V1 in the macaque monkey: a study using immunohistochemistry of anterogradely transported Phaseolus vulgaris-leucoagglutinin', *J Comp Neurol*, **285**(1), pp. 54-72.
Ros, T., Theberge, J., Frewen, P. A., Kluetsch, R., Densmore, M., Calhoun, V. D. & Lanius, R. A. (2013) 'Mind over chatter: plastic up-regulation of the fMRI salience network directly after EEG neurofeedback', *Neuroimage*, **65**, pp. 324-335.
Sadaghiani, L., Gleeson, H. B., Youde, S., Waddington, R. J., Lynch, C. D. & Sloan, A. J. (2016) 'Growth Factor Liberation and DPSC Response Following Dentine Conditioning', *J Dent Res*, **95**(11), pp. 1298-1307.
Sadaghiani, S., Scheeringa, R., Lehongre, K., Morillon, B., Giraud, A. L., D'Esposito, M. & Kleinschmidt, A. (2012) 'Alpha-band phase synchrony is related to activity in the fronto-parietal adaptive control network', *J Neurosci*, **32**(41), pp. 14305-14310.
Sadaghiani, S., Scheeringa, R., Lehongre, K., Morillon, B., Giraud, A. L. & Kleinschmidt, A. (2010) 'Intrinsic connectivity networks, alpha oscillations, and tonic alertness: a simultaneous electroencephalography/functional magnetic resonance imaging study', *J Neurosci*, **30**(30), pp. 10243-10250.
Schnitzler, A. & Gross, J. (2005) 'Normal and pathological oscillatory communication in the brain', *Nat Rev Neurosci*, **6**(4), pp. 285-296.
Siegel, M., Donner, T. H. & Engel, A. K. (2012) 'Spectral fingerprints of large-scale neuronal interactions', *Nat Rev Neurosci*, **13**(2), pp. 121-134.
Smit, C. M., Wright, M. J., Hansell, N. K., Geffen, G. M. & Martin, N. G. (2006) 'Genetic variation of individual alpha frequency (IAF) and alpha power in a large adolescent twin sample', *International Journal of Psychophysiology*, **61**(2), pp. 235-243.
Sridharan, D., Levitin, D. J. & Menon, V. (2008) 'A critical role for the right fronto-insular cortex in switching between central-executive and default-mode networks', *Proceedings of the National Academy of Sciences*, **105**(34), pp. 12569-12574.
Sripada, R. K., King, A. P., Welsh, R. C., Garfinkel, S. N., Wang, X., Sripada, C. S. & Liberzon, I. (2012a) 'Neural dysregulation in posttraumatic stress disorder: evidence for disrupted equilibrium between salience and default mode brain networks', *Psychosomatic medicine*, **74**(9), p. 904.
Sripada, R. K., King, A. P., Welsh, R. C., Garfinkel, S. N., Wang, X., Sripada, C. S. & Liberzon, I. (2012b) 'Neural dysregulation in posttraumatic stress disorder: evidence for disrupted

equilibrium between salience and default mode brain networks', *Psychosom Med*, **74**(9), pp. 904-911.
Sterling, P. (2014) 'Homeostasis vs allostasis: implications for brain function and mental disorders', *JAMA Psychiatry*, **71**(10), pp. 1192-1193.
Tang, A. C., Sutherland, M. T., Sun, P., Zhang, Y., Nakazawa, M., Korzekwa, A., Yang, Z. & Ding, M. (2007) 'Top-Down Versus Bottom-Up Processing in the Human Brain: Distinct Directional Influences Revealed by Integrating SOBI and Granger Causality', in M. E. Davies, C. J. James, S. A. Abdallah & M. D. Plumbley (eds), *Independent Component Analysis and Signal Separation: 7th International Conference, ICA 2007, London, UK, September 9-12, 2007. Proceedings*, Berlin, Heidelberg, Springer Berlin Heidelberg.
Tang, W., Liu, H., Douw, L., Kramer, M. A., Eden, U. T., Hamalainen, M. S. & Stufflebeam, S. M. (2017a) 'Dynamic connectivity modulates local activity in the core regions of the default-mode network', *Proc Natl Acad Sci U S A*, **114**(36), pp. 9713-9718.
Tang, Y. Y., Tang, Y., Tang, R. & Lewis-Peacock, J. A. (2017b) 'Brief Mental Training Reorganizes Large-Scale Brain Networks', *Front Syst Neurosci*, **11**, p. 6.
Thut, G., Schyns, P. G. & Gross, J. (2011) 'Entrainment of perceptually relevant brain oscillations by non-invasive rhythmic stimulation of the human brain', *Front Psychol*, **2**, p. 170.
Uhlhaas, P. J. & Singer, W. (2012) 'Neuronal dynamics and neuropsychiatric disorders: toward a translational paradigm for dysfunctional large-scale networks', *Neuron*, **75**(6), pp. 963-980.
Vossen, A., Gross, J. & Thut, G. (2015) 'Alpha Power Increase After Transcranial Alternating Current Stimulation at Alpha Frequency (alpha-tACS) Reflects Plastic Changes Rather Than Entrainment', *Brain Stimul*, **8**(3), pp. 499-508.
Wang, C., Rajagovindan, R., Han, S. M. & Ding, M. (2016) 'Top-Down Control of Visual Alpha Oscillations: Sources of Control Signals and Their Mechanisms of Action', *Front Hum Neurosci*, **10**, p. 15.
Wolpe, J. & Lazarus, A. A. (1966) *Behaviour Therapy Techniques; A Guide to the Treatment of Neuroses*, New York, Pergamon.
Young, C. B., Raz, G., Everaerd, D., Beckmann, C. F., Tendolkar, I., Hendler, T., Fernandez, G. & Hermans, E. J. (2017) 'Dynamic Shifts in Large-Scale Brain Network Balance As a Function of Arousal', *J Neurosci*, **37**(2), pp. 281-290.
Zaehle, T., Rach, S. & Herrmann, C. S. (2010) 'Transcranial alternating current stimulation enhances individual alpha activity in human EEG', *PLoS One*, **5**(11), p. e13766.
Zhan, Z., Xu, L., Zuo, T., Xie, D., Zhang, J., Yao, L. & Wu, X. (2014) 'The contribution of different frequency bands of fMRI data to the correlation with EEG alpha rhythm', *Brain Res*, **1543**, pp. 235-243.
